# Classification of integers based on residue classes via modern deep learning algorithms

**DOI:** 10.1016/j.patter.2023.100860

**Published:** 2023-10-12

**Authors:** Da Wu, Jingye Yang, Mian Umair Ahsan, Kai Wang

**Affiliations:** 1 Department of Mathematics, University of Pennsylvania, Philadelphia, PA 19104, USA; 2 Department of Pathology, Children’s Hospital of Philadelpha, University of Pennsylvania, Philadelphia, PA 19104, USA

**Keywords:** feature engineering, divisibility rules, machine learning, deep learning, large language models, fourier series, linear regression

## Abstract

Judging whether an integer can be divided by prime numbers such as 2 or 3 may appear trivial to human beings, but it can be less straightforward for computers. Here, we tested multiple deep learning architectures and feature engineering approaches to classifying integers based on their residues when divided by small prime numbers. We found that the ability of classification critically depends on the feature space. We also evaluated automated machine learning (AutoML) platforms from Amazon, Google, and Microsoft and found that, without appropriately engineered features, they failed on this task. Furthermore, we introduced a method that utilizes linear regression on Fourier series basis vectors and demonstrated its effectiveness. Finally, we evaluated large language models (LLMs) such as GPT-4, GPT-J, LLaMA, and Falcon, and we demonstrated their failures. In conclusion, feature engineering remains an important task to improve performance and increase interpretability of machine learning models, even in the era of AutoML and LLMs.

## Highlights

• This study underscores the enduring significance of feature engineering
• AutoML and LLMs cannot classify prime numbers without optimal features
• Fourier series regression can effectively solve this problem
• The effectiveness of integer classification hinges on the features provided

## The bigger picture

Machine learning heavily relies on feature engineering. We tested multiple deep learning architectures with different feature engineering techniques by considering a fundamental problem of classifying integers. We also tested automated machine learning (AutoML) pipelines developed by big tech companies. Our findings suggest that the quality of features provided plays a crucial role in the effectiveness of classification. Moreover, we evaluated prompt-based learning approaches using large language models (LLMs) and identified their shortcomings. As a promising alternative, we introduced a method that utilizes linear regression on Fourier series basis vectors, which proved to be effective for the general case. Therefore, feature engineering remains an important task to improve performance, increase interpretability, and reduce complexity of machine learning models, even in the era of AutoML and LLMs.

## Teaser

This research delves into the importance of feature engineering in machine learning. Feature engineering is like crafting the right tools for computers to learn and make sense of data. This paper focuses on predicting the residues of dividing a whole number by a small prime number such as 2 and 3. Even in an age of sophisticated automated machine learning tools and advanced large language models, the art of feature engineering remains crucial.

## Introduction

The task of determining residue class when dividing a given integer, such as 74 or 243,589, by a prime number, such as 2 or 3, remains an interesting and practical problem. In its simplest form, distinguishing whether an integer is odd or even is straightforward for humans. Merely examining the unit digit is sufficient: if it belongs to the set {0,2,4,6,8}, the number is even; otherwise, it is odd.

On the other hand, classifying integers based on their residues when divided by 3 poses a slightly more difficult challenge. It is well-known that an integer is divisible by 3 if and only if the sum of its numerical digits is divisible by 3. For example, the number 123 is divisible by 3 because 1+2+3=6, which is divisible by 3. Conversely, 59 is not divisible by 3 as the sum of its digits, 5+9=14, is not divisible by 3. Drawing upon this rule, a simple algorithm can be swiftly devised for humans to classify integers based on their residues modulo 3. When confronted with an arbitrary integer *n*, one can first check if it is divisible by 3. If it is, the classification is complete. If not, one can try n+1 or n−1. If either of these numbers is divisible by 3, the process stops. Otherwise, one can try n+2 or n−2. At this stage, termination is necessary as there are only three possible residue classes modulo 3.

Nevertheless, for both cases discussed above, our reliance on mathematical knowledge to design the learning algorithms is absolute. Although no algorithm can achieve satisfactory performance on all possible scenarios based on “no free lunch theorem,”^1^ automated machine learning (AutoML) has attracted significant attention and demonstrated success in many domain-specific problems.^2^^,^^3^^,^^4^ Multiple academic and commercial implementations of AutoML are now available to help users select the best-performing model for a specific problem. Furthermore, a number of large language models (LLMs) have been developed and popularized,^5^ with one well-known example being ChatGPT—a powerful chatbot based on LLMs developed by OpenAI.^6^^,^^7^ These LLMs showed special abilities that are not present in small-scale language models: for example, in addition to memorizing knowledge, LLMs exhibit reasoning abilities when they are sufficiently large.^8^^,^^9^^,^^10^^,^^11^ Despite these recent development in the machine learning space, the question of whether there exists a systematic approach for Turing machines^12^ to autonomously discern patterns from training data and effectively address the integer classification problem appears to be an intriguing and often overlooked issue. This serves as the central problem of our investigation in the current study.

## Results

### Problem setups

As mentioned above, we are interested in the problem of classifying integers based on their residues mod *p*. Due to practical considerations such as representations and memory limitations, we restrict our sample space to non-negative integers up to $2^{32}-1$. For instance, when p=2, our parameter space $X_{2}=Z\cap \left[0,2^{32}-1\right]$ and our label space $Y_{2}=\left\{0,1\right\}$. Given a set of training data $S_{2}=\left\{\left(x_{1},y_{1}\right),\ldots ,\left(x_{n},y_{n}\right),y_{i}\equiv x_{i}\;\mathrm{mod}\;2\right\}\in {\left(X_{2}\times Y_{2}\right)}^{n}$, we want to train a classifier $h_{S_{2}}:X_{2}\mapsto Y_{2}$ to predict whether an “unseen” integer is odd or even. In this case, we have a binary classification problem. For general *p*, the parameter space $X_{p}=Z\cap \left[0,2^{32}-1\right]$ and the label space $Y_{p}=\left\{0,1,\ldots ,p-1\right\}$. The training set $S_{p}=\left\{\left(x_{1},y_{1}\right)\ldots ,\left(x_{n},y_{n}\right):y_{i}\equiv x_{i}\;\mathrm{mod}\;p\right\}$ is sampled from ${\left(X_{p}\times Y_{p}\right)}^{n}\text{,}$ and we hope to build an effective classifier $h_{S_{p}}:X_{p}\mapsto Y_{p}$ to classify integers based on their residues mod *p*. This time we have a multi-class classification problem.

We primarily focus on the cases when p=2 and 3 but also extend some of the analysis to some other small prime numbers, e.g., p=7.

### Preparation of datasets

We uniformly sample integers within the range of $\left[0,2^{32}-1\right]$. The specific size of the datasets may vary in different cases and will be specified later. In addition, we also consider the following feature engineering approaches on non-negative integers:

• Binary representation: For instance, 4 is equal to [0,…,0,1,0,0], 2 is equal to [0,…,0,0,1,0], and 5 is equal to $\left[0,\ldots ,1,0,1\right]$.
• Base-three representation: For example, 3 is equal to [0,…,0,1,0], and 6 is equal to 0$\left[0,\ldots ,0,2\text{,0}\right]$.
• One-gram encoding: We separate the integer into a vector of numerical digits. For instance, 123 will become $\left[0,\ldots ,0,1,2,3\right].$
• Two-gram encoding: We group two consecutive numerical digits together (with overlap) to form our feature vector. For instance, 1234 will become $\left[\left[0,0\right],\ldots ,\left[0,0\right],\left[0,1\right],\left[1,2\right],\left[2,3\right],\left[3,4\right]\right]$.
• Three-gram encoding: We group three consecutive numerical digits together (with overlap). For instance, under three-gram encoding, 1234 will become $\left[\left[0,0,0\right],\ldots ,\left[0,0,0\right],\left[0,0,1\right],\left[0,1,2\right],\left[1,2,3\right],\left[2,3,4\right]\right]$.
• One-gram & two-gram encoding combined: It is the union of one-gram and two-gram encoding.
• One-gram & two-gram & three-gram encoding combined: It is the union of one-gram, two-gram, and three-gram encoding.

All the above feature engineering processes will be tested on both mod 2 and mod 3 cases. In addition to those mentioned above, given the problem nature of mod 3, we also try the following two feature engineering processes:

• One-gram encoding + its sum: In addition to one-gram encoding, we also add sum of all of its numerical digits. For instance, 1234 will become $\left[0,\ldots ,0,1,2,3,4,10\right]$.
• One-gram encoding + (its sum %3): In this case, 1234 will become [0,…,0,1,2,3,4,10%3]=[0,…,0,1,2,3,4,1].

Since we ran experiments in Python, we adopt conventions of Python: The […] means list and the [[…],…,[…]] means nested list (a.k.a. matrix). We also pad additional zeros or zero lists [0,…,0] on the left to make sure that each feature vector has the same dimension. By doing this, we can convert list-like objects into tensor forms so that we can put them into tensorflow neural networks.

### Results from deep neutral networks (DNN)

**Figure 1:**
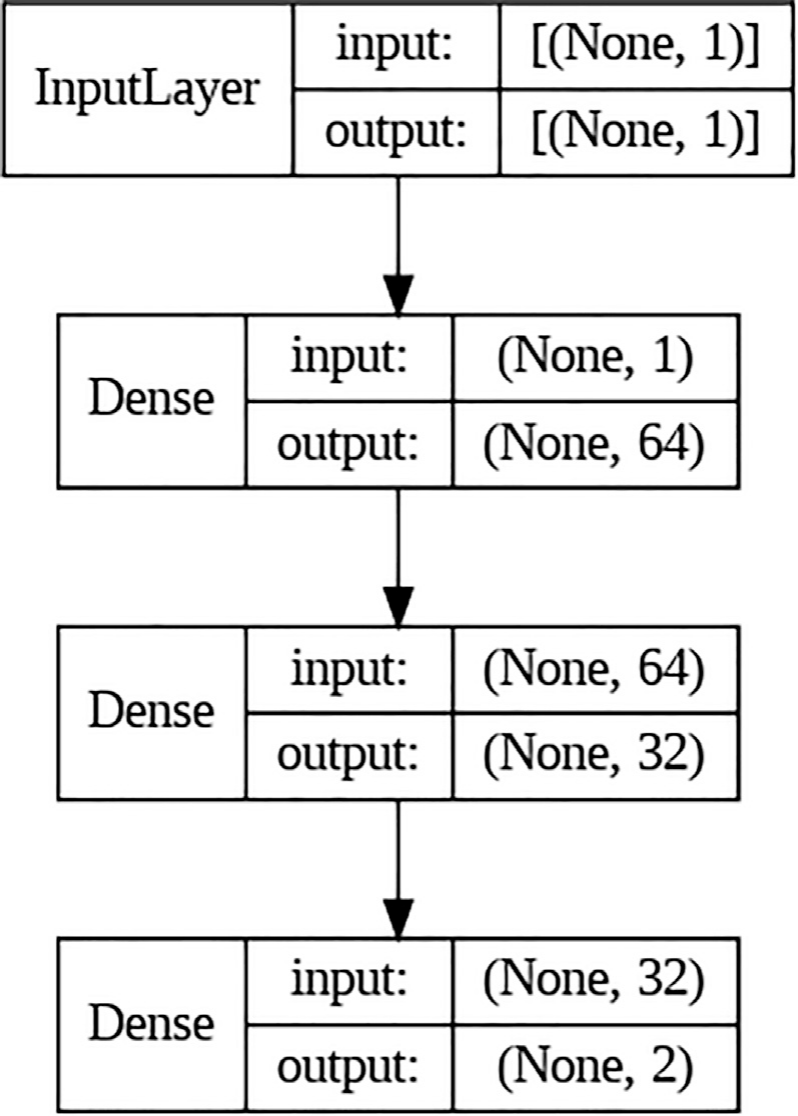
The ANN that we used for training raw data in Subsection "Results from deep neural networks (DNN)."

**Figure 2:**
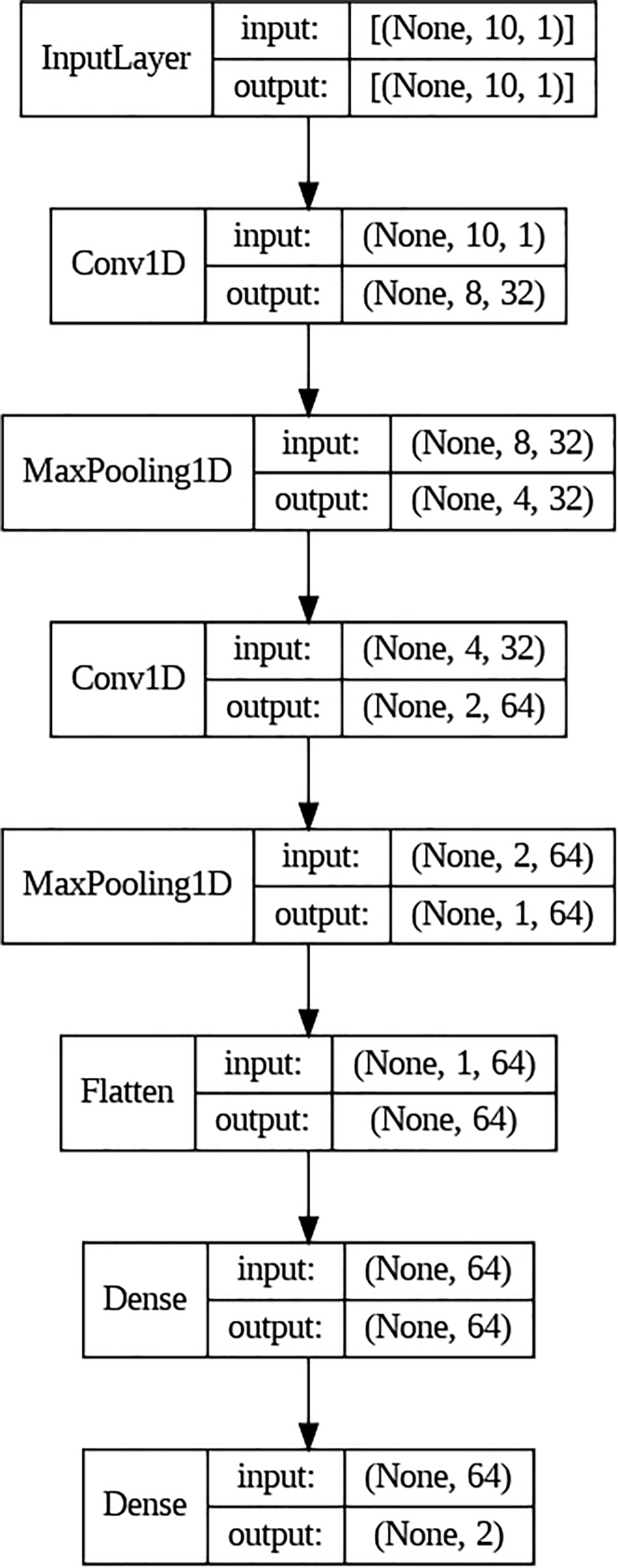
The CNN that we used for training data after one-gram encoding in Subsection "Results from deep neural networks (DNN)."

**Figure 3:**
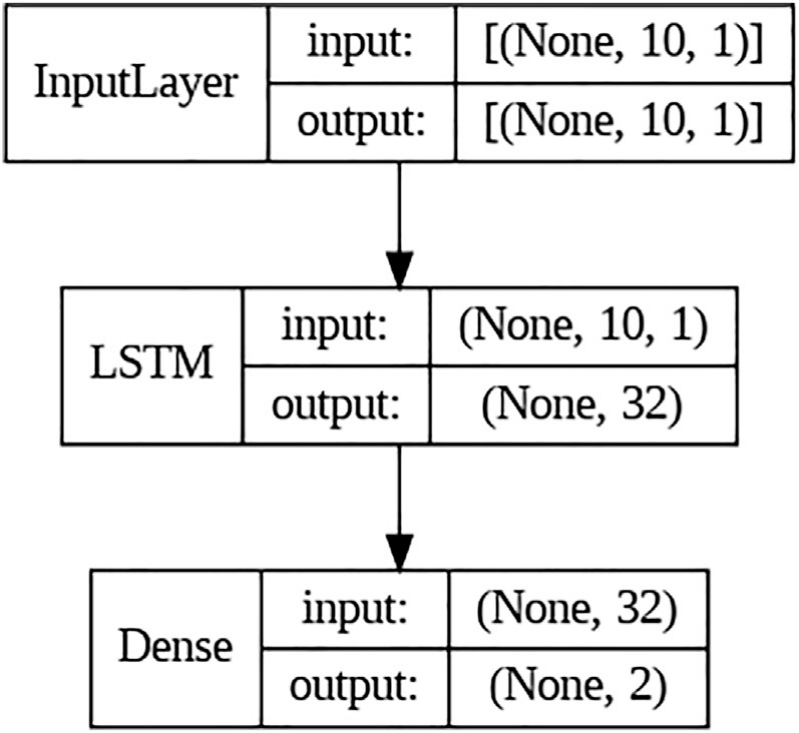
The RNN that we used for training data after one-gram encoding in Subsection "Results from deep neural networks (DNN)."

We first tested on *artificial neural networks* (ANNs). The ANN considered here has 64 neurons in the first layer and 32 neurons in the second layer. The dimensions of the input and output layer depend on the feature engineering process and the number of training labels, respectively. The activation functions are the classical ReLU functions, except that for the output layer, we use the sigmoid function. Besides the classical ANNs, we also tested on a *convolutional neural network* (CNN)^13^^,^^14^ and *recurrent neural network* (RNN).^15^^,^^16^ The CNN considered here has two 1D convolution layers, two max pooling layers, and two dense layers with ReLU activation functions. The RNN considered here has a single LSTM layer and a dense layer with sigmoid activation functions. Finally, we tested on *bidirectional encoder representations from transformers* (BERT),^17^ which is a family of masked language models introduced by Google in 2018. The ANN, CNN, and RNN architectures considered in this study are illustrated in Figures 1, 2, and 3, respectively.

We first discuss the mod 2 case. As listed in Table 1, ANN on raw data only gives an accuracy of ∼0.5. It is not surprising that after converting to binary representations, accuracy of 1.000 can be achieved since we have done mod 2 operations already during binary transformations. The one, two, and three-gram encodings (or their combined versions) do give reasonable hints to the algorithms, and they significantly improve the accuracy to ∼0.8. The BERT model in the end can also achieve an accuracy of 1.000 due to extensive pre-training processes and the state-of-the-art transformer architecture.

**Table 1:** Results on mod 2 via deep neural networks

Classification on divisibility by 2 via deep neural networks		
Algorithms (feature engineering)	Mean	Standard Deviation
Artificial neutral network (ANN)	0.501	0.003
ANN (binary representation)	1.000	0.000
ANN (base-three representation)	0.537	0.002
ANN (one-gram)	0.864	0.024
ANN (two-gram)	0.778	0.048
ANN (three-gram)	0.799	0.002
ANN (one & two-gram combined)	0.786	0.028
ANN (one & two & three-gram combined)	0.781	0.023
Convolutional neural network (one-gram)	0.921	0.042
Recurrent neural network (one-gram)	0.740	0.026
Bidirectional encoder representations from transformers (BERT) (one-gram)	1.000	0.000

Means and standard deviations are calculated with three uniformly sampled, equal-size training sets.

In the mod 3 case, it is expected that base-three representations can achieve an accuracy of 1.000 even under simple network architectures for the same reason as binary representations. The “one-gram + (its sum %3)” encoding can achieve accuracy of >0.9 in all networks tested since it essentially told algorithms everything about the mod 3 divisibility rule.

It is also interesting to see that BERT can achieve an accuracy of 1.000 from “one-gram + its sum” encoding, whereas it can only have an accuracy of ∼0.33 from “one-gram” encoding. In other words, the hint of summing up digits works well with BERT but poorly with other types of deep neural networks. BERT is the only algorithm here that can achieve an accuracy of 1.000 with “one-gram + its sum” encoding in mod 3 case. The key difference between BERT and other neural networks is that in BERT, all the possible sums of the digits (99 potential choices of sums assuming there are 10 digits maximum when we restricted input space to $2^{32}-1$) are embedded into algorithms with length 768 each, and therefore, we have 768×99 more features in BERT instead of having only one additional feature (sum of digits) in other neural networks. Due to large amounts of pre-training data and better embedding techniques, these 768×99 additional features have already been “seen and learned” by BERT, and this helps detect the recursive nature of mod 3 problem. On the contrary, in other neural networks, the sum of digits could be large and therefore completely unseen during training, which leads to inaccuracy.

We found that within Table 1 and Table 2, certain outcomes are distinctly surpassing what would be expected from mere random guessing. For instance, in Table 1, ANN (one-gram) attains an accuracy of 0.864, and ANN (two-gram) achieves an accuracy of 0.778. In all these instances, we believe that further escalating complexities of the model in a visible scale, as evidenced by our experimentation, could potentially drive these outcomes to a perfect score of 1.000. In other words, these cases enjoy the so-called *scaling property*.^18^^,^^19^ This fact can also be confirmed during our experimentation on AutoML platforms later. However, in our experiments later, different classes of models were selected and employed, all of which were tree-based algorithms with specific forms of regularization.

**Table 2:** Results on mod 3 via deep neural networks

Classification on divisibility by 3 via deep neural networks		
Algorithms (feature engineering)	Mean	Standard Deviation
Artificial neutral network (ANN)	0.334	0.002
ANN (binary representation)	0.396	0.002
ANN (base-three representation)	1.000	0.000
ANN (one-gram)	0.343	0.003
ANN (one-gram + its sum)	0.335	0.002
ANN (one-gram + (its sum %3))	1.000	0.000
ANN (two-gram)	0.334	0.002
ANN (three-gram)	0.333	0.001
ANN (one & two-gram combined)	0.332	0.004
ANN (one & two & three-gram combined)	0.334	0.007
Convolutional neural network (CNN) (one-gram)	0.340	0.002
CNN (one-gram + its sum)	0.330	0.002
CNN (one-gram + (its sum %3))	0.936	0.001
Recurrent neural network (RNN) (one-gram)	0.330	0.003
RNN (one-gram + its sum)	0.333	0.001
RNN (one-gram + (its sum %3))	0.934	0.001
Bidirectional encoder representations from transformers (BERT) (one-gram)	0.336	0.005
BERT (one-gram + its sum)	1.000	0.000
BERT (one-gram + (its sum %3))	1.000	0.000

Means and standard deviations are calculated with three uniformly sampled, equal-size training sets.

It is important to note, however, that this phenomenon is not replicated across all the other scenarios. For those cases with outcomes equivalent to random guessing (that is, an accuracy of ∼0.5 for modulo 2 and ∼0.33 for modulo 3), even though the well-regarded *Universal Approximation Theorem*^20^ theoretically assures us of the possibility to perfectly fit training data, it is impractical for individuals to identify the optimal candidates.

### Results from AutoML

We also tested on commonly used AutoML platforms developed by Google,^21^ Microsoft,^22^ and Amazon.^23^ To reduce the computing time and save computing power, we uniformly sample 30,000 non-negative integers from $\left[0,2^{32}-1\right]$.

As can be seen in Table 3 and Table 4, the pre-installed feature engineering algorithms in AutoML pipelines are not effective at all with raw data. This raises an alert that blindly throwing data into AutoML platforms without any feature engineering has a certain (sometimes very high) level of risks; although these AutoML products are extremely powerful, carefully designed, and updated constantly by ML experts, they cannot guarantee to deliver an effective model autonomously. It is crucial to apply domain knowledge to transform data before training.

**Table 3:** Results on mod 2 via AutoML

Feature engineering	Microsoft Azure ML		Google Cloud Vertex AI		Amazon Sagemaker	
	Accuracy	Model	Accuracy	Model	Accuracy	Model
No feature engineering	0.504	RandomForest	0.495	N/A	0.508	WeightedEnsemble
Binary representation	1.000	XGBoost	1.000	N/A	1.000	LightGBM
Base-three representation	0.508	RandomForest	0.506	N/A	0.514	WeightedEnsemble
One-gram	1.000	XGBoost	1.000	N/A	1.000	LightGBM
Two-gram	1.000	LightGBM	1.000	N/A	1.000	LightGBM
Three-gram	1.000	XGBoost	1.000	N/A	1.000	LightGBM
One- & two-gram	1.000	XGBoost	1.000	N/A	1.000	LightGBM
One- & two- & three-gram	1.000	RandomForest	1.000	N/A	1.000	LightGBM

For Microsoft and Amazon, we report the best possible model and their respective performance on test. The Google AutoML platform did not report the specific model and only reported testing statistics.

**Table 4:** Results on mod 3 via AutoML

Feature engineering	Microsoft Azure ML		Google Cloud Vertex AI		Amazon Sagemaker	
	Accuracy	Model	Accuracy	Model	Accuracy	Model
No feature engineering	0.357	RandomForest	0.352	N/A	0.345	WeightedEnsemble
Binary representation	0.361	RandomForest	0.342	N/A	0.345	WeightedEnsemble
Base-three representation	1.000	XGBoost	1.000	N/A	1.000	LightGBM
One-gram	0.352	RandomForest	0.352	N/A	0.348	WeightedEnsemble
One-gram + its sum	1.000	XGBoost	1.000	N/A	1.000	LightGBM
One-gram + (its sum %3)	1.000	LightGBM	1.000	N/A	1.000	LightGBM
Two-gram	0.359	RandomForest	0.353	N/A	0.348	WeightedEnsemble
Three-gram	0.346	RandomForest	0.349	N/A	0.351	WeightedEnsemble
One- & two-gram	0.348	RandomForest	0.351	N/A	0.349	WeightedEnsemble
One- & two- & three-gram	0.346	XGBoost	0.341	N/A	0.345	WeightedEnsemble

For Microsoft and Amazon, we report the best possible model and their respective performance on test. The Google AutoML platform did not report the specific model and only reported testing statistics.

In the case of mod 3, as presented in Table 4, breaking down and/or combining digits without summing them up are not effective. However, summing up all the digits in addition to one-gram encoding can help AutoML platforms deliver classifiers with an accuracy of 1.000. Note that results of AutoML are reported for the best possible model from multi-angle considerations (e.g., complexity, interpretations, etc.), and it appears that these AutoML platforms heavily prefer tree-based algorithms, even though deep learning algorithms were also considered.

### Results from Fourier series regression

Next, we proposed a method using Fourier series regressions and tested on mod 3 and mod 7 problems. In both cases, an accuracy of 1.000 can be achieved. This approach proves effective for handling all values of *p* with a minimal number of training samples, provided that the training dataset size significantly exceeds the value of *p*. To demonstrate this point, this section contains outcomes of both modulo 3 and modulo 7 problems, based on a limited dataset of only 200 samples, divided into 150 for training and validation (135 for training and 15 for validation) and 50 for testing. An accuracy of 1.000 can already be attained for any given test set. Augmenting the sample size by a factor of 10 or 100 does not exhibit any noticeable impact on accuracy and only imparts minimal influence on regression estimations.

Here, our total dataset consists of 200 uniform samples from $\left[0,2^{32}-1\right]$, among which 150 samples are used to estimate regression coefficients, and the remaining 50 ones are used for testing purposes. Fix integer p=3 or 7. Suppose $X={\left[x_{1},\ldots ,x_{n}\right]}^{T}$ is our training set, and let $Y_{p}={\left[y_{1},\ldots ,y_{n}\right]}^{T}$ be the vector of training labels, i.e., $y_{i}\equiv x_{i}\;\mathrm{mod}\;p$, where $y_{i}\in \left\{0,1\ldots ,p-1\right\}$ and p∈{3,7}. For each j=1,2,…,[p/2], let

(Equation 1) $$\sin\left(\frac{2\pi j}{p}X\right)={\left[\sin\left(\frac{2\pi j}{p}\cdot x_{1}\right),\ldots ,\sin\left(\frac{2\pi j}{p}\cdot x_{n}\right)\right]}^{T}$$

and

(Equation 2) $$\cos\left(\frac{2\pi j}{p}X\right)={\left[\cos\left(\frac{2\pi j}{p}\cdot x_{1}\right),\ldots ,\cos\left(\frac{2\pi j}{p}\cdot x_{n}\right)\right]}^{T}$$

be vectors of Fourier series basis, and we consider the following linear regression:

(Equation 3) $$Y_{p}=\gamma +\sum_{j=1}^{\left[p/2\right]}\left(\alpha_{j}\;\sin\left(\frac{2\pi j}{p}X\right)+\beta_{j}\;\cos\left(\frac{2\pi j}{p}X\right)\right)+\varepsilon \text{,}$$

where ε denotes the vector of standard Gaussian noise. All the coefficients in (Equation 3) are estimated by classical *ordinary least square* (OLS) regression.

Generally speaking, the linear regression model is not a good candidate for classification problems due to a number of reasons, one of them being that the output values are continuous instead of categorical. However, in our case, due to the large number of training samples, predicted values on the testing set are very close to integers (see Table 5 and Table 6) so that we can round them to the nearest integer if their distance is within $10^{-5}$.

**Table 5:** Values of the regression estimate $\hat{Y_{3}}\left(X\right)$ (Equation 4) at integer points

*X*	$\hat{Y_{3}}\left(X\right)$	Xmod3
0	$-5.473399511402022\cdot 10^{-14}$	0
1	1.0000002331249993	1
2	1.9999997668748912	2
3	$-5.46229728115577\cdot 10^{-14}$	0
4	1.0000002331249984	1
5	1.999999766874892	2
6	$-5.440092820663267\cdot 10^{-14}$	0
7	1.0000002331249984	1
8	1.999999766874892	2
9	$-5.4289905904170155\cdot 10^{-14}$	0

**Table 6:** Values of the regression estimate $\hat{Y_{7}}\left(X\right)$ (Equation 5) at integer points

*X*	$\hat{Y_{7}}\left(X\right)$	Xmod7
0	$3.1012281631603855\cdot 10^{-8}$	0
1	1.0000009260275013	1
2	1.9999998780188157	2
3	3.000000361534261	3
4	3.9999997004903016	4
5	5.000000184005747	5
6	5.999999135997062	6
7	$3.101228274182688\cdot 10^{-8}$	0
8	1.000000926027501	1
9	1.9999998780188162	2
10	3.000000361534261	3
11	3.999999700490302	4
12	5.000000184005747	5
13	5.999999135997064	6
14	$3.1012283852049904\cdot 10^{-8}$	0
15	1.0000009260274998	1
16	1.9999998780188164	2

#### Regression estimates of mod 3

**Figure 4:**
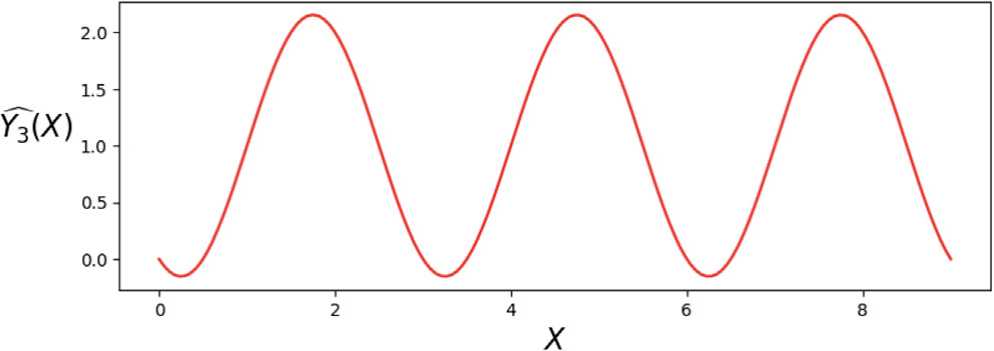
Plot of regression estimate ${\hat{Y}}_{3}\left(X\right)$ (Equation 4).

By using LinearRegression package in sklearn of Python, we have the following regression coefficients estimate:

(Equation 4) $$\hat{Y_{3}}\left(X\right)=0.9999999556293363+\left(-0.57735\right)\cdot \sin\left(\frac{2\pi }{3}X\right)+\left(-1.00000\right)\cdot \cos\left(\frac{2\pi }{3}X\right)\text{,}$$

with $R^{2}$ value being 0.9999999999999453 and all coefficients being statistically significant. The $R^{2}$ value is computed under train-validation split ratio 0.1. The plot of regression estimate (Equation 4) and its values at integer points, covering three periods, are recorded in Figure 4 and Table 5 respectively. In the mod 3 case, accuracy of 1.000 can be achieved. We also want to emphasize that the joint presence of both sines and cosines is needed, and if we, for instance, remove all the cosines and only keep the sines in (Equation 3), then $R^{2}$-value is only 0.2533, and accuracy is 0.3206 with the same dataset. On the contrary, we can also add more pairs of sine and cosine, e.g., j=1,…p−1, in our Fourier series basis (Equation 1) and (Equation 2). This will also give us a satisfying linear regression estimate (accuracy 1.000) with “less overshoot and undershoot” compared to (Equation 3). However, the regression table will give the potential warning of *multicollinearity*,^24^^,^^25^^,^^26^ indicating that too many features were added, which may cause instability of regression coefficients estimates in some large *p* cases. Therefore, our linear regression model (Equation 3) is *optimal*.

#### Regression estimates of mod 7

**Figure 5:**
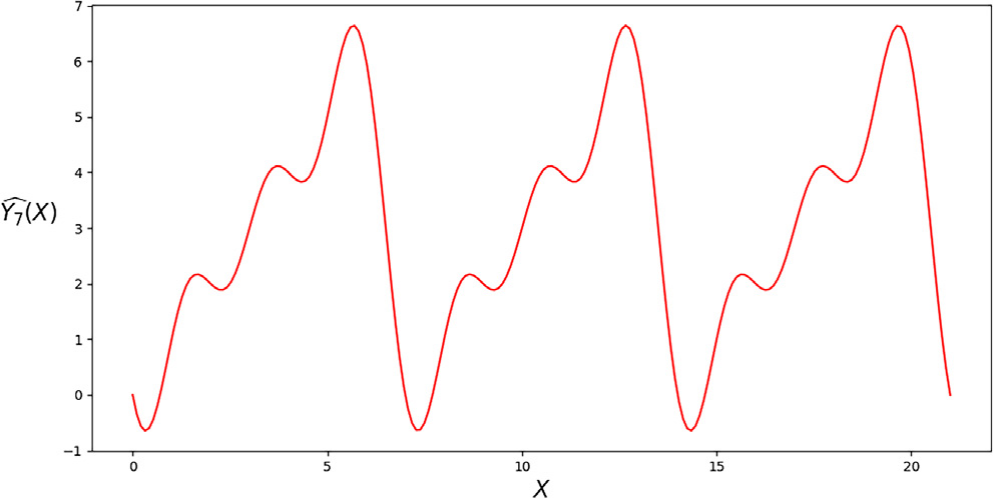
Plot of regression estimate ${\hat{Y}}_{7}\left(X\right)$ (Equation 5).

In the case of mod 7, we have the following coefficients estimates:

(Equation 5) $$\hat{Y_{7}}\left(X\right)=3.0000000310122816+\left(-2.076521\right)\cdot \sin\left(\frac{2\pi }{7}X\right)+\left(-1.000000\right)\cdot \cos\left(\frac{2\pi }{7}X\right)+\left(-0.797473\right)\cdot \sin\left(\frac{4\pi }{7}X\right)+\left(-1.000000\right)\cdot \cos\left(\frac{4\pi }{7}X\right)+\left(-0.228243\right)\cdot \sin\left(\frac{6\pi }{7}X\right)+\left(-1.000000\right)\cdot \cos\left(\frac{6\pi }{7}X\right)\text{,}$$

with $R^{2}$-value being 0.9999999999999203 and all coefficients being statistically significant. Again, accuracy of 1.000 can be achieved on the testing set. The plot of regression estimate (Equation 5) and its values at integer points, covering two periods, are recorded in Figure 5 and Table 6, respectively.

### Results from large language models

Next, we conducted tests to assess the proficiency of open-source LLMs, specifically GPT-J-6B,^27^^,^^28^ LLaMA-7B,^29^ and Falcon-40B,^30^ in their understanding of divisibility rules. Specifically, for each prime *p* up to 31, we tried the following two prompts:

• P1: “There are various mathematical rules to check if an integer is divisible by *p*, for instance”
• P2: “How to check if an integer is divisible by *p*, for instance we can”

We utilized these two prompts to better steer the open-source models toward producing mathematical answers as opposed to algorithmic ones. Additionally, we included “for instance …” since open-source models are more suitable for completing sentences rather than providing direct answers to questions.

The comprehensive responses are presented in the supplemental experimental procedures. Within this context, we manually assess the accuracy and informativeness of responses generated by those models. The elaborated outcomes are recorded in Table 7. For the “Correct” notation, we refer to the *mathematical accuracy of the provided answers*. For the “Informative” notation, we assess *whether the models effectively articulate the divisibility rule in a lucid and cohesive manner*. As an example, when the model attempts to employ the modulo operator (%)—while its mathematical accuracy is unquestionable—it ultimately lacks any informative value. The data presented in Table 7 demonstrates the inadequacy of these open-source LLMs for primes beginning at 7. Even when dealing with smaller primes, they consistently produce incorrect and uninformative replies.

**Table 7:** The proficiency of LLMs in providing both accurate mathematical solutions and informative explanations regarding divisibility rules by prime numbers up to 31

Prime *p*	GPT-J-6B				LLaMA-7B				Falcon-40B				ChatGPT-175B	
	Correct		Informative		Correct		Informative		Correct		Informative		Correct	Informative
	P1	P2	P1	P2	P1	P2	P1	P2	P1	P2	P1	P2	P	P
2	no	no	yes	no	no	yes	no	no	no	no	no	no	yes	yes
3	no	no	no	no	yes	yes	yes	yes	yes	yes	yes	no	yes	yes
5	no	no	no	no	yes	yes	yes	yes	yes	yes	no	no	yes	yes
7	yes	no	yes	no	no	no	no	no	no	no	no	no	yes	yes
11	no	no	no	no	no	no	no	no	no	no	no	no	yes	yes
13	no	no	no	no	no	no	no	no	no	no	no	no	yes	yes
17	no	no	no	no	no	no	no	no	no	no	no	no	yes	yes
19	no	no	no	no	no	no	no	no	no	no	no	no	no	yes
23	no	no	no	no	no	no	no	no	no	no	no	no	yes	no
29	no	no	no	no	no	no	no	no	no	no	no	no	yes	no
31	no	no	no	no	no	no	no	no	no	no	no	no	yes	no

The detailed responses are documented in Notes S1–S3 and Table S1–S6.

Furthermore, we assessed ChatGPT’s^31^ closed-source implementation utilizing reinforcement learning on the knowledge of divisibility rules. All outcomes can be entirely replicated, and to ensure alignment with open-source models, we also incorporate ChatGPT’s evaluations in Table 7. Specifically, we used the following prompt:

• P: “How to check if an integer is divisible by *p*?”.

Regarding ChatGPT, utilizing the aforementioned prompts (P1 and P2) is unnecessary, as it possesses a strong capability to comprehend the genuine intention behind the prompt P. The comprehensive results are furnished in Tables S1–S6. Notable enhancements are observed compared to open-source LLMs. Nevertheless, beginning at 23, ChatGPT resorts to using the modulo operator % to tackle the issue, resulting in completely uninformative answers. As a result, its adequacy for larger primes still lags behind.

Finally, we tested the latest GPT-4 with code interpreter (distinct from the aforementioned ChatGPT) on its capabilities of designing deep neural networks to address the integer divisibility problems. Note that as of now, access to GPT-4 is exclusively offered to subscribers of ChatGPT plus. We showcased its code in the supplemental experimental procedures. While correct, it does not possess the capability to formulate an efficient algorithm, which encompasses identifying appropriate feature engineering techniques and selecting the optimal ML/DL algorithms.

Hence, it can be deduced that current state-of-the-art LLMs do not possess the required capability to offer dependable and accurate information regarding divisibility rules.

## Discussion

In our current study, we conducted extensive experiments to delve into classifications of large finite integers, specifically those up to $2^{32}-1$, based on their residues when divided by small prime numbers. Our investigation involved testing various deep neural network architectures and employing diverse feature engineering approaches. The obtained results were both intuitive and straightforward to interpret.

An important observation that emerged throughout our analysis is that, regardless of complexities of network structures or specific neural network frameworks used, the performance of our classification task was highly reliant on the feature space provided to deep learning models. This discovery remained consistent not only for neural network architectures but also when evaluating AutoML pipelines.

Feature engineering is often a challenging and non-intuitive process in practical scenarios, requiring extensive trial-and-error iterations.^32^^,^^33^^,^^34^ In addition to directly engineering features on training samples, there are other avenues where domain expertise can be leveraged to enhance the quality of classifiers. Inspired by the *recurring pattern* exhibited by the modulus function, we devised a simple method that utilizes linear regression with Fourier series basis vectors to capture and understand its periodic behavior. This method exhibited exceptional performance, achieving a perfect accuracy of 1.000 for all modulus *p* problems, even when *p* is not necessarily prime. Furthermore, our proposed approach offers advantages such as minimal training size, reduced time complexity, and improved interpretability of the model, outperforming all the other state-of-the-art ML/DL models in these aspects.

To expand our evaluation further, we also examined the performance of GPT-J-6B, LLaMA-7B, and Falcon-40B concerning divisibility by primes up to 31. Regrettably, our investigation uncovered that these open-source LLMs exhibited a tendency to produce inaccurate and uninformative replies even when primes *p* are small. Additionally, we conducted tests on closed-source ChatGPT and observed a relatively improved performance compared to the aforementioned open-source LLMs. However, it is important to note that ChatGPT still demonstrated some instances of erroneous information, particularly when dealing with larger prime number *p*. For p≥23, it cannot provide any informative answers at all. Finally, we tested the latest GPT-4 with code interpreter on its capabilities of designing an effective neural network to address the mod *p* problem and found that it still lacks the competence to do so.

In conclusion, our study emphasizes the ongoing importance of feature engineering in the AutoML and LLM era. We demonstrated that performances of deep learning models are heavily reliant on carefully engineered features. Without appropriate feature engineering, it is impossible to enhance performance merely by increasing complexities of algorithms on a feasible level or enlarging sizes of training datasets; despite the renowned universal approximation theorem^20^ offering a theoretical assurance that achieving a perfect fit to the training data is possible, it is impractical for individuals to effectively discover the desired candidates in practice. Moreover, our proposed method employing linear regression on Fourier series basis vectors showcased exceptional accuracy, lower time complexity, and enhanced interpretability. Finally, we caution against relying on LLMs for divisibility by large primes, as our findings indicated that they may provide inaccurate information in these cases.

## Experimental procedures

### Resource availability

#### Lead contact

Further information and requests for data should be directed to and will be fulfilled by the lead contact, Dr. Kai Wang (wangk@chop.edu).

#### Materials availability

This study did not generate new unique materials.

### Deep neutral networks

The ANN considered in this study has 64 neurons in the first layer and 32 neurons in the second layer. The dimensions of the input and output layer depend on what feature engineering process we are using and how many training labels there are. The activation functions are the classical ReLU functions, except that for the output layer, we use the sigmoid function. Notice that this is the only ANN considered in this study, and we will not compare the results with other more complex ANNs. This consideration is due to the facts that (1) the above neural network is sufficient for us to demonstrate the importance of feature engineering, and (2) the more complex networks will be tested on the commonly used AutoML platforms.

Besides the classical ANNs, we also considered CNN and RNN. CNN is a well-known deep learning algorithm inspired by the natural vision perception mechanisms of living creatures. The modern framework of CNN was established by LeCun et al.^36^ and later improved.^37^ For recent advances in CNN and its applications, see Gu et al.^38^ and the references therein. The RNN architecture was mainly designed to overcome the issue of “limited context memory” in Bengio et al.^39^: only a fixed number of previous words can be taken into account to predict the next word. In RNN, the context length was extended to indefinite size, which can handle arbitrary context lengths. See De Mulder et al.^40^ for the review on the RNN model and its applications on the statistical language modeling. The CNN we used has two 1D convolution layers, two max pooling layers, and two dense layers with ReLU activation functions. The RNN we used has a single LSTM layer and a dense layer with sigmoid activation functions. We used 10 epochs with a batch size of 32 during training.

Finally, we tested on BERT, which is a family of masked language models introduced by Google in 2018.^17^ It is based on the transformer encoders, and it was pre-trained simultaneously on two tasks: *masked language modeling* and *next sentence prediction*. After pre-training, it can be *fine-tuned* on smaller datasets to optimize its performance on specific tasks, including text classifications, language inference, etc. See also Lin et al., Han et al., Khan et al., and Ganesh et al.^41^^,^^42^^,^^43^^,^^44^ for more discussions on the BERT model and the transformer architecture. Here we tested the BERT model on both mod 2 and 3 problems via the BertForSequenceClassification package in Python. We used 10 epochs with a batch size of 64 and a learning rate of $10^{-6}$.

### Automated machine learning

We considered three commonly used AutoML platforms: Microsoft Azure ML Studio,^22^ Google Cloud Vertex AI,^21^ and Amazon AWS Sagemaker.^23^ All of them are easily accessible online with limited free credits. One can upload the datasets in the “.csv” format and pre-specify the target value, type of tasks (classification, regression, NLP, etc.), primary metric of evaluation, etc.

### Fourier series regressions

In this section, we described our Fourier series regression method in detail. Fix an integer p≥2. Let $X={\left[x_{1},\ldots ,x_{n}\right]}^{T}$ be our training set, and let $Y_{p}={\left[y_{1},\ldots ,y_{n}\right]}^{T}$ be the vector of training labels, i.e., $y_{i}\equiv x_{i}\;\mathrm{mod}\;p\text{,}$ where $y_{i}\in \left\{0,1\ldots ,p-1\right\}$. For each $x_{i}$ in *X*, consider the following Fourier series basis:

(Equation 6) $$\left\{\sin\left(\frac{2\pi j}{p}\cdot x_{i}\right),\cos\left(\frac{2\pi j}{p}\cdot x_{i}\right),\text{where}\;j=1,2,\ldots ,\left[p/2\right]\right\}\text{.}$$

For each *j*, let

$$\sin\left(\frac{2\pi j}{p}X\right)={\left[\sin\left(\frac{2\pi j}{p}\cdot_{1}\right),\ldots ,\sin\left(\frac{2\pi j}{p}\cdot x_{n}\right)\right]}^{T}$$

and

$$\cos\left(\frac{2\pi j}{p}X\right)={\left[\cos\left(\frac{2\pi j}{p}\cdot x_{1}\right),\ldots ,\cos\left(\frac{2\pi j}{p}\cdot x_{n}\right)\right]}^{T}$$

be the vectors of Fourier series basis, and consider the following linear regression equation:

(Equation 7) $$Y_{p}=\gamma +\sum_{j=1}^{\left[p/2\right]}\left(\alpha_{j}\;\sin\left(\frac{2\pi j}{p}X\right)+\beta_{j}\;\cos\left(\frac{2\pi j}{p}X\right)\right)+\varepsilon \text{,}$$

where ε denotes the vector of standard Gaussian noise. All the coefficients in (Equation 7) are estimated by the classical OLS method. For prediction, we round the predicted values from linear regression (Equation 7) to the nearest integer if their distance is within $10^{-5}$.

### Large language models

Finally, we tested the knowledge of GPT-J-6B,^27^ LLaMA-7B,^29^ and Falcon-40B^45^ on divisibility rules of prime numbers. These models are all open-sourced and can be easily implemented using hugging face API.^30^^,^^46^^,^^47^

For demonstration purposes, we conducted tests on several scenarios where *p* took on the primes up to 31. For each prime *p*, we employed the following two prompts for each of these three models, ensuring consistency across the evaluations.

1. “How to check if an integer is divisible by *p*, for instance we can”
2. “There are various mathematical rules to check if an integer is divisible by *p*, for instance”

The maximum token lengths for GPT-J-6B, LLaMA-7B, and Falcon-40B are set to be 100,160, and 200 respectively. For ChatGPT,^31^ we used “How to check if an integer is divisible by *p*?” as the prompt. For GPT-4, we used “Can you design a deep learning algorithm to determine if a number can be divided by *p*?”

## Data Availability

Our study did not generate any new data. All the publicly available code for producing training data (uniformly sampled integers), conducting testing using various feature engineering approaches, and implementing distinct deep learning algorithms outlined in Table 1 and Table 2, as well as the Fourier series regression discussed in Section "Results from Fourier series regression," can be accessed at Reference 35.^35^

## Supplemental information

Document S1. Tables S1–S6 and supplemental experimental procedures

Document S2. Article plus supplemental information
