# Supplementary material for: Classification of integers based on residue classes via modern deep learning algorithms
Source: Patterns (N Y). 2023 Oct 12;4(12):100860. doi: 10.1016/j.patter.2023.100860 (PMC10724366; doi:10.1016/j.patter.2023.100860)
Supplement: Document S1. Tables S1–S6 and supplemental experimental procedures [file mmc1.pdf]

Patterns, Volume *4*

Supplemental information

Classification of integers based on residue classes via modern deep learning algorithms

Da Wu, Jingye Yang, Mian Umair Ahsan, and Kai Wang

SUPPLEMENTAL EXPERIMENTAL PROCEDURES

Here we document the prompts and detailed responses from LLMs to complement the contents in the Section "Results from Large Language Models (LLMs)" within main texts.

Responses from GPT-J-6B

When $p = 2$, we have the following two responses:

- **Prompt**: *There are various mathematical rules to check if an integer is divisible by 2, for instance*
  GPT-J: There are various mathematical rules to check if an integer is divisible by 2, for instance. For the numbers in your list, only the 1, 3, 9, and 17 are divisible by 2. For the others, divide by 2 to check that they work.\n\nA:\n \n This question was posted a while ago. This answer works for numbers in any base! Here's the full code that does what I posted before:\ndef divisibility(n

- **Prompt**: *How to check if an integer is divisible by 2, for instance we can*
  GPT-J: How to check if an integer is divisible by 2, for instance we can create some code that will check if a number is even or not and if it is even it will return true and if it is odd then it returns false and we can make code to check if a number in divisible by 3\n\nhow to check if an number is divisible by 2, for instance we can create some code that will check if a number is even or not and if it is even it will

When $p = 3$, we have the following two responses:

- **Prompt**: *There are various mathematical rules to check if an integer is divisible by* 3*, for instance*
  GPT-J: There are various mathematical rules to check if an integer is divisible by 3, for instance.\nAnother one to check if an integer is divisible by 5 or by 7.\n \nIn any case, these rules will be implemented differently in different programming languages.\nTo check if an integer is divisible by 3, you need to look and see if it's divisible by 3, if it is, it has to be divisible by 3. \nNot at all the same in

- **Prompt**: *How to check if an integer is divisible by* 3*, for instance we can*
  GPT-J: How to check if an integer is divisible by 3, for instance we can see that:\n\n- a number divisible by 3 has a remainder of 0 when divided by 3 is 0 or, in other words, 3 (i.e., $3\%3 = 0$)\n\n- a number divisible by 3 has a remainder of 1 when divided by 3 (i.e., $3/3 = 1$)\n\n- a number divisible by

When $p = 5$, we have the following two responses:

- **Prompt**: *There are various mathematical rules to check if an integer is divisible by* 5*, for instance*
  GPT-J: There are various mathematical rules to check if an integer is divisible by 5, for instance by multiplying every number with 5 until no integers remain that are divisible. Of course, in this specific example, you can get away with writing a loop. But for more complex rules, such as "Divisible by 5 until the number is divisible no more" or for the most general case where there might be several factors, it is more natural to use a recursion.\nIf we wanted to

- **Prompt**: *How to check if an integer is divisible by* 5*, for instance we can*
  GPT-J: How to check if an integer is divisible by 5, for instance we can do:\n»> x = 3\n»> a = 5\n»> b = len(x//a)\n»> b // 2\n2\n\nBut we can check if it is more easy using bitwise operators:\n»> x = 31\n»> a = 5\n»> x & a\n14\n\n»> x & 3\n0\n\n»> x & 5\n0\n\n»> x & 7

When $p = 7$, we have the following two responses:

- **Prompt**:*There are various mathematical rules to check if an integer is divisible by* 7*, for instance*
  GPT-J: There are various mathematical rules to check if an integer is divisible by 7, for instance this one:\nint divisibleby$7 = x\%7 == 0$;\n\nBut this one is new to me.\nint isDivisibleBy7(int x) \n if $(x! = 0\&\&(x\%7) == 0)$\n return true;\n else \n

- **Prompt**: *How to check if an integer is divisible by* 7*, for instance we can*
  GPT-J: How to check if an integer is divisible by 7, for instance we can use something like this :\nfunction ifDivisibleby7(num)\n if(num $\%7 == 0$)\n return true;\n else\n return 'The result is not divisible by 7, please check the value.';\n \n\n\nBut I plaza like to know if it is possible to use a single function to check this.

When $p = 11$, we have the following two responses:

- **Prompt**:*There are various mathematical rules to check if an integer is divisible by* 11*, for instance*
  GPT-J: There are various mathematical rules to check if an integer is divisible by 11, for instance:\nint x = 711;\nx % 11 == 0\n\nor \nx % 11 == 0 && x - x / 11!= 0\n\nbut I believe there is an easier way, using bitwise operators, which I do not understand.\n\nA:\n\nYou can use the bitwise AND (&) operator and the bitwise EQUALS to compare the two numbers.

- **Prompt**: *How to check if an integer is divisible by* 11*, for instance we can*
  GPT-J: How to check if an integer is divisible by 11, for instance we can check if 22 = 4 $*$ 11 because 2 is divisible by 11.\n\nA:\n\nThe only simple way I can think of is to do this using modular arithmetic, which is quite quick and probably not any slower than your own method.\nif x % 11 == 0:\n # x is evenly divisible by 11.\n\nA:\n\nI think the way to go

When $p = 13$, we have the following two responses:

- **Prompt**:*There are various mathematical rules to check if an integer is divisible by* 13*, for instance*
  GPT-J: There are various mathematical rules to check if an integer is divisible by 13, for instance:\n\nIf we take out the 2 from 3*4=12, we get this equation:\n\nIf we take out the zero from zero, we get:\n\nIt is not difficult to see that all the possibilities are divisible by 13.\n\nHowever, if we take out the zero from zero, we get:\n\nSince there are no numbers that are divisible by 13

- **Prompt**: *How to check if an integer is divisible by* 13*, for instance we can*
  GPT-J: How to check if an integer is divisible by 13, for instance we can check if a number is equal to 15 (for example, 2 is not equal to 15 because 14 > 14, so this is a correct answer):\n\nBut I do not know where to start and I need some help on this topic.\nI started to think about writing an algorithm for it, but I'm not sure if it's the correct way.\n\nA:\n\nI think you're correct

When $p = 17$, we have the following two responses:

- **Prompt**: *There are various mathematical rules to check if an integer is divisible by* 17*, for instance*
  GPT-J: There are various mathematical rules to check if an integer is divisible by 17, for instance the rule\nis x is divisible by 17 \nif and only if x= 2k, for some k, with k itself an integer.\nIs there a similar rule for the problem of checking if an integer is divisible by 23? I want to do it in JavaScript.\n\nA:\n\nThere are infinitely many numbers divisible by 17, but only 24 that aren't div.

- **Prompt**: *How to check if an integer is divisible by* 17*, for instance we can*
  GPT-J: How to check if an integer is divisible by 17, for instance we can do:\nvar number = 12;\n\nikiCheck(number,'17'); //returns true\n\nikiCheck(number,'1701'); //returns true\n\nikiCheck(number,'1800'); // returns false as 1800 is not divisible by 17\n\nikiCheck() prizmarija from the iki library, is a great tool to check if a number is divisible by

When $p = 19$, we have the following two responses:

- **Prompt**: *There are various mathematical rules to check if an integer is divisible by* 19*, for instance*
  GPT-J: There are various mathematical rules to check if an integer is divisible by 19, for instance:\n\nif (int value = 19;) System.out.println("Error: the value must be divisible by 19");\n\nBut what is the proper way to check a numberś divisibility by all the numbers from one to 9?\n\nA:\n\nYou could use something like that:\nprivate static Boolean isDividedByOneToNine(int number)

- **Prompt**: *How to check if an integer is divisible by* 19*, for instance we can*
  GPT-J: How to check if an integer is divisible by 19, for instance we can do this in the following way\n number = 1;\nif ((number % 19 == 0)) \n// do something\n\n\nBut this will always return false\nBut if we could check if the number is divisible by a defined integer (ex: 19), then we can do this \nnumber = 1;\nif (((number

When $p = 23$, we have the following two responses:

- **Prompt**: *There are various mathematical rules to check if an integer is divisible by* 23*, for instance*
  GPT-J: There are various mathematical rules to check if an integer is divisible by 23, for instance, and a test for division by 24.\n\nIn a program, this is done by the rules: if m is divisible by n then it divides by n.\n\nThe rule for division by 24 is called modulo division, and can be generalized to modulo r. By modulo r, an integer a is said to be a multiple of r, written $a = r * m$ as

- **Prompt**: *How to check if an integer is divisible by* 23*, for instance we can*
  GPT-J: How to check if an integer is divisible by 23, for instance we can check if a month is divisible by 4 or 5 or 6. \nI tried the following: \nif (((month.month % 4) == 0) || ((month.month % 5) == 0) || ((month.month % 6) == 0))\n System.out.println("Divisible by 4, 5 or 6");\n else \n

When $p = 29$, we have the following two responses:

- **Prompt**: *There are various mathematical rules to check if an integer is divisible by* 29*, for instance*
  GPT-J: There are various mathematical rules to check if an integer is divisible by 29, for instance, 29%3 = 0 or (I guess) 29%13 = 0, etc. But the algorithm could also be applied to a large number of factors.\n\nFor instance, if a number is factorised by a prime (say, 17), then the resulting prime factors could be applied to the original number, to check if that number is prime.\n\nFor example, 2^17*

- **Prompt**: *How to check if an integer is divisible by* 29*, for instance we can*
  GPT-J: How to check if an integer is divisible by 29, for instance we can divide it into 2 parts and ask if they match with 29?\nSo, I wonder how do I check if a given number is divisible by 29, such that (i.e.)\nif (n % 29 == 0) \n ...\n\n\nA:\n\nHow to check if an integer is divisible by 29, for instance we can divide it into 2 parts

When $p = 31$, we have the following two responses:

- **Prompt**: *There are various mathematical rules to check if an integer is divisible by* 31*, for instance*
  GPT-J: There are various mathematical rules to check if an integer is divisible by 31, for instance the following piece of code will display the output as 1,4,1,4,1,4,1,4,1,...\n divisible31 = 31;\nnumber = 1;\nwhile (number<=10^6)\n\n print number % divisible31.

- **Prompt**: *How to check if an integer is divisible by* 31*, for instance we can*
  GPT-J: How to check if an integer is divisible by 31, for instance we can check the value 4 and the result should be true.\n\n\n\nAs it has been said in another post, we are going to use a bitmask.\n\nA bitmask is a number where each bit is represented with a "0" or a "1". The sum of all the bits is always going to be the total number.\n\nFor example:\n\n4 is

Responses from Falcon-40B

When $p = 2$, we have the following two responses:

- **Prompt**: *There are various mathematical rules to check if an integer is divisible by 2, for instance*
  Falcon-40B: There are various mathematical rules to check if an integer is divisible by 2, for instance if the sum of the digits are even then it is divisible by 2 but the sum of the digits are odd doesn't mean it's not divisible by 2, in fact 1 is divisible by 2. So, how can you check whether an integer is divisible by 2? Here is another method of checking if an integer is divisible by 2, if you take the integer and replace all the digits with its equivalent binary number and check whether the binary number is divisible by 2, you will find that the original number is divisible by 2 or not.
  Example:
  - Is 23 divisible by 2? Yes/No
  - Is 45 divisible by 2? Yes/No
  - What is the binary representation $45 = 1011 = 1(2^0) + 1$

- **Prompt**: *How to check if an integer is divisible by 2, for instance we can*
  Falcon-40B: How to check if an integer is divisible by 2, for instance we can check by checking the last bit of the integer.
  Example: 10 is not divisible by 2, since the last bit is not a 2. But 12 is divisible by 2 since the last bit is 2. So we have to check the last bit and then check the last bit. We can do this by converting the integer to its binary representation (in a string) and then checking the last character, if it is equal to '1' then this number is divisible by 2. If this number does not have an even number of digits, then we need to add zeros to it. For this problem, we have added the number of leading zeros to each element. Then we can check that this binary string is divisible by 10 (by checking its parity). So, if it is divisible by 10, then we

When $p = 3$, we have the following two responses:

- **Prompt**: *There are various mathematical rules to check if an integer is divisible by* 3*, for instance*
  Falcon-40B: There are various mathematical rules to check if an integer is divisible by 3, for instance if the sum of its digits is divisible by 3.
  For 2, 3, 5, and 7 it is easy as these are prime numbers.
  For others :
  - check if the digits add up to a single digit number that is divisible by 3.
  - check if the numbers is divisible by 9 as 9 and multiples of 9 are also divisible by 3.
  This is the first time i am writing in this forum. Please correct me if i am wrong

- **Prompt**: *How to check if an integer is divisible by* 3*, for instance we can*
  Falcon-40B: How to check if an integer is divisible by 3, for instance we can use remainder operator in java. For example, if we want to check if integer num is divisible by 3, we can write following code: int num = 12; int mod; mod = num $\%3$; System.out.println(num+" is divisible by 3? "+(mod==0)); Above program would print following result: 12 is divisible by 3? true

When $p = 5$, we have the following two responses:

- **Prompt**: *There are various mathematical rules to check if an integer is divisible by* 5*, for instance*
  Falcon-40B: There are various mathematical rules to check if an integer is divisible by 5, for instance. If the unit's place of the integer is a multiple of 5 or ends in a 0 or 5, you know the integer is a multiple of five too. The rule I will be explaining in this article is how to check for the divisibility of an integer by 7. There is one simple rule for checking it and one other rule, which is more complicated but can help to make the checking easier. Divisibility by 7 The simple rule is that an integer is divisible by 7 if the sum of its digits is a multiple of 7. Examples - The number 1, which is the smallest integer which is divisible by 7, has a unit place (1) that ends with a 0. (0*1) + (1*1) =2,

- **Prompt**: *How to check if an integer is divisible by* 5*, for instance we can*
  Falcon-40B: How to check if an integer is divisible by 5, for instance we can use this formula to check if an integer N is divisible by 5.
  N is divisible by 5 if and only if (N & 1) == 1
  For instance,
  N% 2 == 0 if (N & 1) == 0 <=> N%5 == 0 if (N & 1) == 1
  The code to check if an integer is divisible by 5 is
  int isdivisibleby5(int N) return (N & 1) == 1;
  If you find this post on "Check if divisible by 5".

When $p = 7$, we have the following two responses:

- **Prompt**: *There are various mathematical rules to check if an integer is divisible by* 7*, for instance*
  Falcon-40B: There are various mathematical rules to check if an integer is divisible by 7, for instance:
  - 7 x 2 = 14, the sum of the digits is divisible by 7, (14 / 7 = 2)
  - 7 x 6 = 42, the sum of the digits is divisible by 7 (42 / 7 = 6)
  - 7 x 7 = 49, the sum of the digits is divisible by 7 (49 / 7 = 7)
  So you can check divisibility by 7 by multiplying a candidate number by 7 and check if the result is divisible by 7. This is called the 'trial division test'.
  For larger integers it is quicker to check if the remainder of the number divided by 7 is a multiple of 7. In the case it is a multiple of

- **Prompt**: *How to check if an integer is divisible by* 7*, for instance we can*
  Falcon-40B: How to check if an integer is divisible by 7, for instance we can use modulo operator in Java. We will get remainder by dividing one number by another. If the remainder is 0 then it is divisible by 7. We are using the $\%$ operator here. Java Program to check if number is divisible by 7 import java.util.Scanner; public class Main public static void main(String[] args) int num, remainder; Scanner scanner = new Scanner(System.in); System.out.println("Enter any number."); num = scanner.nextInt(); System.out.printf("7 is a factor of $\%$d.\n", num); remainder = num $\%$ 7; if(remainder == 0) System.out.println(num + " is a multiple of 7."); else System.out.println(num + " is

When $p = 11$, we have the following two responses:

- **Prompt**: *There are various mathematical rules to check if an integer is divisible by* 11*, for instance*
  Falcon-40B: There are various mathematical rules to check if an integer is divisible by 11, for instance, if the sum of its digits is divisible by 11. This problem is different. The input is given as a (potentially huge) integer n. Your function needs to check the divisibility by 11 of that integer. If the integer is not divisible by 11, the function should return 0, otherwise it needs to return 1. The function should not throw exceptions of any kind. Examples:
  input: 10 output: 1 input: -10 output: 0 input: 1111 output: 1
  Note that the input value could be of any size. If the input value is too big to store in an Int or Long, your function can return false, or throw an exception, or do anything else that indicates failure. However, you do need to test for the divisibility of the values 11 and

- **Prompt**: *How to check if an integer is divisible by* 11*, for instance we can*
  Falcon-40B: How to check if an integer is divisible by 11, for instance we can use this trick to check if any given year is a leap year or not.

The idea is based on the fact that the sum of the digits of a number is a multiple of 11 if and only if the number itself is a multiple of 11.
For Example:
Year 1976 has sum of digits 2 7 1 9 = 21
21 = 2*11, So it's a Leap year
How it works?
- Take the given number (integer).
- Sum all its digits.
- If the sum is a multiple of 11, Then it is a leap year, otherwise not.
Example:
#include <iostream> #include <cmath> using namespace std; bool isLeap(int n) int s = 0; while (n

When $p = 13$, we have the following two responses:

- **Prompt**: *There are various mathematical rules to check if an integer is divisible by* 13*, for instance*
  Falcon-40B: There are various mathematical rules to check if an integer is divisible by 13, for instance, if the last digit of an integer is divisible by 2 then the whole number would be divisible by 13. Here is the logic for that:
  - If a number is divisible by 13, the digit sum of the number should be divisible by 2 and not by 3 or 9
  - If a number is not divisible by 13, the sum of the digits of the number should also be divisible by 2, 3 and 9.

- **Prompt**: *How to check if an integer is divisible by* 13*, for instance we can*
  Falcon-40B: How to check if an integer is divisible by 13, for instance we can use a simple trick. If the integer number is divisible by 5 and divisible by 2 then it is divisible by 13. So lets see some simple C++ code. #include <iostream> using namespace std; void checkDivBy13(int n) $/*$ If a number is a multiple of 2 or 5, it is also a multiple of 10. */ int div10 = (n % 10) == 0; int div5 = (n % 5) == 0; if (div10 || div5) cout « n « " is divisible by 13" « endl; void main() checkDivBy13(1); checkDivBy13(2); checkDivBy13(3); checkDiv

When $p = 17$, we have the following two responses:

- **Prompt**: *There are various mathematical rules to check if an integer is divisible by* 17*, for instance*
  Falcon-40B: There are various mathematical rules to check if an integer is divisible by 17, for instance by using the remainder after division by 17, or using the divisibility by 17 rule for odd and even integers. Here is the detail for how to check if a number is divisible by 17.
  1. Integer is Divisible by 17 Rule for Even Integers To find out if an even integer is divisible by 17, the last digit must be 7.
  For even numbers: 2x + 5
  - If the last digit is 7, then the number is divisible by 17.
  - If the last digit is not 7, then the number is not divisible by 17.
  2. Integer is Divisible by 17 Rule for Odd Integers To find out if an odd integer is divisible by 17, the last 3 digits must be divisible by 17.

- **Prompt**: *How to check if an integer is divisible by* 17*, for instance we can*
  Falcon-40B: How to check if an integer is divisible by 17, for instance we can check if 17 is a prime number, is the number divisble by 2, or any number divisble by 3,4,... This program checks if a given integer n is divisible by both 17 and 9. This code is contributed by the user jatin. C Output:
  Enter an integer to check whether its prime: 17 Prime Number.
  Enter an integer to check whether its prime: 18 Non Prime Number.
  Please write comments if you find anything incorrect, or you want to share more information about the topic discussed above.

When $p = 19$, we have the following two responses:

- **Prompt**: *There are various mathematical rules to check if an integer is divisible by* 19*, for instance*
  Falcon-40B: There are various mathematical rules to check if an integer is divisible by 19, for instance:
  - 19 / 1 = 19 is divisible by 19 because 19 × 1/1 = 19
  - 19 / 2 = 9 and 9/2 = 4 remainder 1
  - 19 / 19 = 1 and 1/19 = 0 remainder 1
  - 19 / 38 = 0 and 38/19 = 2 remainder 0
  - 19 / 57 = 0 and 57/19 = 3 remainder 0
  - 19 / 76 = 0 and 76/19 = 4 remainder 0
  - 19 / 95 = 0 and 95/19 = 5 remainder 0

- **Prompt**: *How to check if an integer is divisible by* 19*, for instance we can*
  Falcon-40B: How to check if an integer is divisible by 19, for instance we can use % (modulus operator) and divide by 19, then check if the remainder is 0 (zero), if yes then the number is divisible by 19. Example: The following is how to check if the number 19 is divisible by 19 using modulus and logical AND operator. int isDivisibleBy19(int n)
  return n % 19 == 0;

  The following is how to check for the divisibility of 19 using the division sign (/) and logical AND (&&). int isDivisibleBy19(int n)
  return n % 19 == 0 && n!= 0;

  You can find more information on this topic at:

When $p = 23$, we have the following two responses:

- **Prompt**: *There are various mathematical rules to check if an integer is divisible by* 23*, for instance*
  Falcon-40B: There are various mathematical rules to check if an integer is divisible by 23, for instance: - (23*23) % 23 == 0
  - (23*23*23) % 23 == 0
  - (23*23*23*23) % 23 == 0
  -... But I found a simple rule to check if the number is divisible by 23, and I wonder if it could be proven : 1 + 2 + 3 +... + N = N(N+1)/2 is divisible by 23. I tried to look at the proof in the other thread (the one linked at the beginning) but I didn't understand it.

- **Prompt**: *How to check if an integer is divisible by* 23*, for instance we can*
  Falcon-40B: How to check if an integer is divisible by 23, for instance we can check 12345 is divisible by 23 then we have to check the unit digits of 456, the unit digits of 456 are 6, then we have to check if the unit digits of 6 are divisible by 23, so 6 divided by 23 is 0. 456 is divisible by 23. We can find whether an integer is divisible by 23 or not by using the Modulo (The program below will check if an integer is divisible by 23 or not. #include <stdio.h> int main() int number, remainder, divisor = 23; printf("Enter an integer: "); scanf("%d", &number); remainder = number % divisor; if(remainder == 0) printf("The given integer is

When $p = 29$, we have the following two responses:

- **Prompt**: *There are various mathematical rules to check if an integer is divisible by* 29*, for instance*
  Falcon-40B: There are various mathematical rules to check if an integer is divisible by 29, for instance: - If you add the digits of the number, the sum must be divisible by 3. If not, add 1 to this sum. - If you divide this number by 8, it must have a remainder of 1, 2, 3, 4, 5 or 6. - The sum of the numbers in all rows of a Pythagorean triplet with 29 as a base, must be divisible by 29. If the integer is not divisible by 29, then it must be divisible by one or multiple of 5, because 5 is also a factor. So, you must first check if the integer is divisible by 5. If the integer is not divisible by both 3 and 5, it is certainly not divisible by 29. So, you

- **Prompt**: *How to check if an integer is divisible by* 29*, for instance we can*
  Falcon-40B: How to check if an integer is divisible by 29, for instance we can check if 1 is divisible by 29 or not.
  If an integer is divisible by 29, it would have either 0 or 29 as a remainder. So in our case, we will do: 1 % 29 = 0
  or
  1 % 29 = 29
  This method is called modulo (or modulus) and is very useful and used often in computer science. It is also known as remainder.
  If the remainder is 0, then the integer is divisible by 29. If it is a positive non-zero then the integer is not divisible by 29.
  In this case,
  - 1 % 29 = 0
  - 2 % 29 = 2
  - 3 % 29 = 3

When $p = 31$, we have the following two responses:

- **Prompt**: *There are various mathematical rules to check if an integer is divisible by* 31*, for instance*
  Falcon-40B: There are various mathematical rules to check if an integer is divisible by 31, for instance, the integer is divisible by 31 if the last digit is divisible by 3 or if the last two digits are divisible by 6. Here we will discuss some

of the simple methods to check if an integer is evenly divisible by 31 or not. Check Divisibility by 3: An integer can be evenly divisible by 31 only if its last digit is divisible by 3 or if all of the digits are multiples of 3, for example, 12345 and 123456789. Therefore, if the last digit of any number is not a multiple of 3 or all the digits are not multiple of 3, we can say that the number is not evenly divisible by 31. Therefore, if the last digit of the number is a multiple of 3 or all the digits are divisible by 3, then the

- **Prompt**: *How to check if an integer is divisible by* 31*, for instance we can*
  Falcon-40B: How to check if an integer is divisible by 31, for instance we can find out if a number is divisible by 31 by dividing 31 with that number. But if the number is a large one, it is time consuming to find out the result. In this article we are going to show two easy methods to help you check if a number is divisible by 31. One is manual way, another is by using an online tool. The manual method is to check each digit of number from right to left. If a digit is greater than or equal to 5, we have to add 1 to the quotient. If the quotient is not divisible by 31, the number is not divisible by 31.
  Here is an example: The number is 1023. First check if the last digit 3 is >=5, it is greater, we add 1 to the quotient and get 6/10

Responses from LLaMA-7B

When $p = 2$, we have the following two responses:

- **Prompt**: *There are various mathematical rules to check if an integer is divisible by 2, for instance*
  LLaMA: There are various mathematical rules to check if an integer is divisible by 2, for instance 2, 4, 6, 8, 10, 12, 14, 16, 18, 20, 22, 24, 26, 28, 30, 32, 34, 36, 38, 40, 42, 44, 46, 48, 50, 52, 54, 56, 58, 60, 62, 64, 66, 68, 70, 72, 74, 76, 78, 80, 82,
- **Prompt**: *How to check if an integer is divisible by 2, for instance we can*
  LLaMA: How to check if an integer is divisible by 2, for instance we can check if 10 is divisible by 2 by dividing 10 by 2 and checking if the result is an integer.\nIf the result is an integer then the number is divisible by 2, if the result is not an integer then the number is not divisible by 2.\nIn the above example, 10 is divisible by 2 because 10/2 = 5 and 5 is an integer.\nIn the above example, 11 is not divisible by 2 because 11/2 = 5.5 and 5.5 is not an integer.\nIn the above example, 10 is divisible by 2 because $10\%2 = 0$ and 0 is

When $p = 3$, we have the following two responses:

- **Prompt**: *There are various mathematical rules to check if an integer is divisible by* 3*, for instance*
  LLaMA: There are various mathematical rules to check if an integer is divisible by 3, for instance 3n+1, 3n+2, 3n+0, 3n-1, 3n-2, 3n-0.\nBut I have found a simple rule to check if an integer is divisible by 3.\nIf the sum of the digits of an integer is divisible by 3, then the integer is divisible by 3.\nFor example, 123456789 is divisible by 3 because 1+2+3+4+5+6+7+8+9=45 and 45 is divisible by 3.\nIf the sum of the digits of an integer is not divisible by 3, then the integer is not divisible
- **Prompt**: *How to check if an integer is divisible by* 3*, for instance we can*
  LLaMA: How to check if an integer is divisible by 3, for instance we can check if 12 is divisible by 3 by dividing 12 by 3 and checking if the remainder is 0.\nWe can also check if an integer is divisible by 3 by checking if the sum of the digits of the integer is divisible by 3.\nFor instance, 12 is divisible by 3 because 1 + 2 = 3 and 3 is divisible by 3.\n123 is divisible by 3 because 1 + 2 + 3 = 6 and 6 is divisible by 3.\n1234 is divisible by 3 because 1 + 2 + 3 + 4 = 10 and 10 is divisible by

When $p = 5$, we have the following two responses:

- **Prompt**: *There are various mathematical rules to check if an integer is divisible by* 5*, for instance*
  LLaMA: There are various mathematical rules to check if an integer is divisible by 5, for instance, if the number is divisible by 5, then the last digit must be 0, 5, or 0.\nThe number 12345 is divisible by 5 because the last digit is 5.\nThe number 123456 is not divisible by 5 because the last digit is 6.\nThe number 1234567 is not divisible by 5 because the last digit is 7.\nThe number 12345678 is not divisible by 5 because the last digit is 8.\nThe number 12345678
- **Prompt**: *How to check if an integer is divisible by* 5*, for instance we can*
  LLaMA: How to check if an integer is divisible by 5, for instance we can check if 12 is divisible by 5 by doing 12 % 5 = 2.\nHow to check if an integer is divisible by 10, for instance we can check if 12 is divisible by 10 by doing 12 % 10 = 2.\nHow to check if an integer is divisible by 15, for instance we can check if 12 is divisible by 15 by doing 12 % 15 = 2.\nHow to check if an integer is divisible by 20, for instance we can check if 12 is divisible by 20

When $p = 7$, we have the following two responses:

- **Prompt**: *There are various mathematical rules to check if an integer is divisible by* 7*, for instance*
  LLaMA: There are various mathematical rules to check if an integer is divisible by 7, for instance 7 divides n if and only if the sum of the digits of n is divisible by 7.\nThe following program uses this rule to check if a number is divisible by 7.\n// Check if a number is divisible by 7.\n// Sum of the digits of the number.\n// Check if the sum is divisible by 7.\n// Print the result of the test.\n// The number is divisible by 7.\n// The number is not divisible by 7.\nThe following program uses the same rule to check if a number is divisible by 7.\n// Check if a number is divisible by 7 using the sum of the digits.\n// The number is not divisible by 7.

- **Prompt**: *How to check if an integer is divisible by* 7*, for instance we can*
  LLaMA: How to check if an integer is divisible by 7, for instance we can check if 123456789 is divisible by 7.\nThe first step is to check if the last digit of the number is divisible by 7.\nIf the last digit is divisible by 7, then we can check if the number formed by the first n-1 digits is divisible by 7.\nIf the number formed by the first n-1 digits is divisible by 7, then the number is divisible by 7.\nIf the number formed by the first n-1 digits is not divisible by 7, then the number is not divisible by 7.\nIf the last digit is not divisible by 7, then the number is not divisible by 7.\nThe.

When $p = 11$, we have the following two responses:

- **Prompt**: *There are various mathematical rules to check if an integer is divisible by* 11*, for instance*
  LLaMA: There are various mathematical rules to check if an integer is divisible by 11, for instance, if the last digit is 1, 3, 7 or 9, then the number is divisible by 11.\nIf the last digit is 2, 4, 6 or 8, then the number is not divisible by 11.\nIf the last digit is 0, 5 or 6, then the number is divisible by 11.\nIf the last digit is 1, 3, 7 or 9, then the number is divisible by 11.\nIf the last digit is 2, 4, 6 or 8, then the number is not divisible

- **Prompt**: *How to check if an integer is divisible by* 11*, for instance we can*
  LLaMA: How to check if an integer is divisible by 11, for instance we can check if 11 is divisible by 11.\nThe following code will check if an integer is divisible by 11.\nThe following code will check if an integer is divisible by 11. The code will return true if the integer is divisible by 11.\nThe following code will check if an integer is divisible by 11. The code will return false if the integer is not divisible by 11.\nThe following code will check if an integer is divisible by 11. The code will return true if the integer is divisible by 11. The code will return false if the integer is not divis

When $p = 13$, we have the following two responses:

- **Prompt**: *There are various mathematical rules to check if an integer is divisible by* 13*, for instance*
  LLaMA: There are various mathematical rules to check if an integer is divisible by 13, for instance, if the number is divisible by 13, then the sum of the digits of the number must be divisible by 13.\nThe sum of the digits of a number is the sum of the digits of the number.\nThe sum of the digits of a number is the sum of the digits of the number. The sum of the digits of a number is the sum of the digits of the number.\nThe sum of the digits of a number is the sum of the digits of the number. The sum of the digits of a number is the sum of the digits of the number. The sum of the digits of a number is the sum of the digits of the

- **Prompt**: *How to check if an integer is divisible by* 13*, for instance we can*
  LLaMA: How to check if an integer is divisible by 13, for instance we can check if 123456789 is divisible by 13.\nThe first step is to check if the number is divisible by 10.\nIf the number is divisible by 10, then it is divisible by 13.\nIf the number is not divisible by 10, then it is not divisible by 13.\nThe number 123456789 is divisible by 10, so it is divisible by 13.\nThe number 123456789 is not divisible by 10, so it is not

When $p = 17$, we have the following two responses:

- **Prompt**: *There are various mathematical rules to check if an integer is divisible by* 17*, for instance*
  LLaMA: There are various mathematical rules to check if an integer is divisible by 17, for instance 17 is a prime number, so it is divisible by 1 and 17.\n17 is divisible by 17, because 17 = 17 x 1.\n17 is divisible by 17, because 17 = 17 x 1. 17 is divisible by 17, because 17 = 17 x 1. 17 is divisible by 17, because 17 = 17 x 1. 17 is divisible by 17, because 17 = 17 x 1. 17 is divisible by 17, because 17 = 17 x 1. 1

- **Prompt**: *How to check if an integer is divisible by* 17*, for instance we can*
  LLaMA: How to check if an integer is divisible by 17, for instance we can check if 17 divides 1000.\nWe can check if 17 divides 1000 by using the modulo operator.\nThe modulo operator is denoted by $\%$.\nThe modulo operator returns the remainder of the division of two numbers.\nFor example, 1000 $\%$ 17 = 15.\nSo, 17 divides 1000.\n1000 $\%$ 17 = 15\n15 $\%$ 17 = 15\n15 $\%$ 17 = 15 and so on.\nSo, 17 divides 15.\n15 $\%$ 17 = 15 and so on.\nSo,

When $p = 19$, we have the following two responses:

- **Prompt**: *There are various mathematical rules to check if an integer is divisible by* 19*, for instance*
  LLaMA: There are various mathematical rules to check if an integer is divisible by 19, for instance, if the integer is divisible by 19, then the sum of the digits of the integer must be divisible by 19.\nThe sum of the digits of an integer is the sum of the digits of the integer.\nThe sum of the digits of an integer is the sum of the digits of the integer. The sum of the digits of an integer is the sum of the digits of the integer.\nThe sum of the digits of an integer is the sum of the digits of the integer. The sum of the digits of an integer is the sum of the digits of the integer. The sum of the digits of an integer is the sum of the digits of the

- **Prompt**: *How to check if an integer is divisible by* 19*, for instance we can*
  LLaMA: How to check if an integer is divisible by 19, for instance we can check if 19 is divisible by 19.\nThe following code will check if an integer is divisible by 19.\nThe following code will check if an integer is divisible by 19. The code will return true if the integer is divisible by 19.\nThe following code will check if an integer is divisible by 19. The code will return false if the integer is not divisible by 19.\nThe following code will check if an integer is divisible by 19. The code will return true if the integer is divisible by 19. The code will return false if the integer is not divis

When $p = 23$, we have the following two responses:

- **Prompt**: *There are various mathematical rules to check if an integer is divisible by* 23*, for instance*
  LLaMA: There are various mathematical rules to check if an integer is divisible by 23, for instance, if the number is divisible by 23, then the sum of the digits of the number must be divisible by 23.\nThe sum of the digits of a number is the sum of the digits of the number.\nThe sum of the digits of a number is the sum of the digits of the number. The sum of the digits of a number is the sum of the digits of the number.\nThe sum of the digits of a number is the sum of the digits of the number. The sum of the digits of a number is the sum of the digits of the number. The sum of the digits of a number is the sum of the digits of the

- **Prompt**: *How to check if an integer is divisible by* 23*, for instance we can*
  LLaMA: How to check if an integer is divisible by 23, for instance we can check if 123456789 is divisible by 23.\nThe first step is to check if the number is divisible by 3.\nIf the number is divisible by 3, then it is divisible by 23.\nIf the number is not divisible by 3, then it is not divisible by 23.\nIf the number is divisible by 3, then it is divisible by 23. If the number is not divisible by 3, then it is not divisible by 23.\nThe number is divisible by 3.\nThe number is not divisible

When $p = 29$, we have the following two responses:

- **Prompt**: *There are various mathematical rules to check if an integer is divisible by* 29*, for instance*
  LLaMA: There are various mathematical rules to check if an integer is divisible by 29, for instance, if the number is even, then it is divisible by 29. If the number is odd, then it is not divisible by 29.\nIf the number is divisible by 29, then the last digit of the number is 0, 2, 4, 6, 8 or 9.\nIf the number is not divisible by 29, then the last digit of the number is 1, 3, 5, 7 or 0.\nIf the number is divisible by 29, then the sum of the digits of the number is divisible by 29.\nIf

- **Prompt**: *How to check if an integer is divisible by* 29*, for instance we can*
  LLaMA: How to check if an integer is divisible by 29, for instance we can check if 12 is divisible by 29.\nThe first step is to check if the number is divisible by 2.\nIf the number is divisible by 2, then we can check if it is divisible by 29.\nIf the number is not divisible by 2, then we can check if it is divisible by 29.\nIf the number is divisible by 2, then we can check if it is divisible by 29. If the number is not divisible by 2, then we can check if it is divisible by 29.\nIf the number is divisible by 2

When $p = 31$, we have the following two responses:

- **Prompt**: *There are various mathematical rules to check if an integer is divisible by* 31*, for instance*
  LLaMA: There are various mathematical rules to check if an integer is divisible by 31, for instance, if the number is divisible by 31, then the sum of the digits of the number must be divisible by 31.\nThe number 31 is a prime number.\nThe number 31 is a composite number.\nThe number 31 is a Fibonacci number.\n The number 31 is a Harshad number.\nThe number 31 is a Sophie Germain prime.\n The number 31 is a Chen prime.\nThe number 31 is a safe prime.\nThe number 31 is a happy number.\nThe number 31 is a centered square number.\n

- **Prompt**: *How to check if an integer is divisible by* 31*, for instance we can*
  LLaMA: How to check if an integer is divisible by 31, for instance we can check if 123456789 is divisible by 31.\nThe first step is to check if the number is divisible by 3.\nIf the number is divisible by 3, then it is divisible by 31.\nIf the number is not divisible by 3, then it is not divisible by 31.\nIf the number is divisible by 3, then it is divisible by 31. If the number is not divisible by 3, then it is not divisible by 31.\nIf the number is divisible by 3, then it is divisible by

Responses from GPT-4 with code interpreter

- **Prompt:** Can you design a deep learning algorithm to determine if a number can be divided by 17?
  GPT-4: Certainly! But before we delve into a deep learning solution, it's important to note that traditional algorithms can efficiently determine whether a number is divisible by 17. The division operation or using the divisibility rule for 17 can solve this problem in a straightforward manner.

  However, if you're interested in a deep learning approach for educational purposes or to see how a neural network might handle this task, we can attempt that. Here's a basic outline of a deep learning model to solve this problem:

  1. **Dataset Generation**: Generate a dataset containing numbers and their labels: 1 if divisible by 17, 0 otherwise. - For instance:

     17 -> 1,
     34 -> 1,
     16 -> 0,
     35 -> 0,
     ...

  2. **Data Representation**: Represent numbers in a binary format so they can be input to the neural network.
  3. **Neural Network Model**: You can use a simple feed-forward neural network (like MLP) for this task. A deep architecture might be overkill, but the purpose here is experimentation.
  4. **Training**: Use the dataset from step 1 to train the neural network. The model will learn to recognize patterns in the binary representation of numbers to determine divisibility.

  Here's a simple model using TensorFlow/Keras:

```
import numpy as np
from tensorflow import keras
from keras.models import Sequential
from keras.layers import Dense, Input
from keras.utils import to_categorical

# Data generation
samples = 100000
X = np.array(range(samples))
y = np.where(X % 17 == 0, 1, 0)

# Convert numbers to binary format
max_length = int(np.ceil(np.log2(samples)))
X_binary =
np.array([list(format(x, '0{}b'.format(max_length))) for x in X],dtype=np.uint8)

# Split data into train and test sets
split = int(0.8 * samples)
X_train, X_test = X_binary[:split], X_binary[split:]
y_train, y_test = y[:split], y[split:]

# Neural network model
model = Sequential([
    Input(shape=(max_length,)),
    Dense(128,activation='relu'),
    Dense(64,activation='relu'),
    Dense(32,activation='relu'),
    Dense(1,activation='sigmoid')
])

model.compile(optimizer='adam',loss='binary_crossentropy',metrics=['accuracy'])
model.fit(X_train,y_train,epochs=10,batch_size=32,validation_data=(X_test,y_test))
```

```
# Evaluation
loss, accuracy = model.evaluate(X_test, y_test)
print(f"Accuracy:{accuracy*100:.2f}%")
```

Remember, this is a fun experiment to see if deep learning can learn the pattern, but in practice, using traditional algorithms is much more efficient for this particular problem.

SUPPLEMENTAL ITEMS

Prime N	Solution by ChatGPT on divisibility rule by N
2	To check if an integer is divisible by 2, you can use a very simple rule: check the last digit of the integer. If the last digit is an even number (0, 2, 4, 6, or 8), the integer is divisible by 2. If the last digit is an odd number, the integer is not divisible by 2. For example:<br>1. The integer 246 is divisible by 2 because the last digit is 6 (an even number).<br>2. The integer 357 is not divisible by 2 because the last digit is 7 (an odd number). **[Correct]**
3	To check if an integer is divisible by 3, you can use a simple rule based on the sum of its digits. If the sum of the digits of the integer is divisible by 3, then the integer itself is divisible by 3.<br>Here's the method:<br>1. Add up all the digits of the integer.<br>2. If the sum of the digits is divisible by 3 (including if it's 0), then the original integer is also divisible by 3.<br>Let's illustrate this method with an example: Let's check if the integer 351 is divisible by 3: Sum of digits: 3 + 5 + 1 = 9. Since 9 is divisible by 3 (3 * 3 = 9), the original integer (351) is also divisible by 3. **[Correct]**
5	To check if an integer is divisible by 5, you can use a simple rule: check the last digit of the integer. If the last digit is either 0 or 5, the integer is divisible by 5. If the last digit is not 0 or 5, the integer is not divisible by 5. For example: The integer 240 is divisible by 5 because the last digit is 0. **[Correct]**
7	To check if an integer is divisible by 7, you can use the following method:<br>1. Take the last digit of the integer.<br>2. Multiply this last digit by 2.<br>3. Subtract the result from step 2 from the remaining part of the integer (excluding the last digit).<br>4. If the resulting number is divisible by 7 (including if it's 0), then the original integer is also divisible by 7.<br>Let's illustrate this method with an example: Let's check if the integer 154 is divisible by 7:<br>1. Last digit: 4<br>2. Multiply last digit by 2: 4 * 2 = 8<br>3. Remaining part of the integer after removing the last digit: 15<br>4. Subtract the result from step 2 from the remaining part: 15 - 8 = 7<br>Since 7 is divisible by 7 (7 * 1 = 7), the original integer (154) is also divisible by 7. **[Correct]**

Table S1. Response given by ChatGPT on the question "*How to check if an integer can be divided by* N" for $N = 2, 3, 5$ and 7. The bold face texts "**[Correct]**" or "**[Wrong]**" indicate whether the response from ChatGPT is a mathematically correct statement or not. This is related to section "Results from Large Language Models (LLMs)" in the main texts. The rest Table S2,S3,S4,S5,S6 follow the exact same spirit.

Prime N	Solution by ChatGPT on divisibility by N
11	To check if an integer is divisible by 11, you can use the following method:<br>1. Starting from the rightmost digit of the integer, alternately add and subtract the digits.<br>2. If the result is divisible by 11 (including if it's 0), then the original integer is also divisible by 11.<br>Let's illustrate this method with an example:<br>Let's check if the integer 363 is divisible by 11:<br>1. Starting from the rightmost digit: 3<br>2. Move to the next digit and subtract it: 3 - 6 = -3<br>3. Move to the next digit and add it: -3 + 3 = 0<br>Since the result is 0, which is divisible by 11 (11 * 0 = 0), the original integer (363) is also divisible by 11.<br>Let's try another example with the integer 154:<br>1. Starting from the rightmost digit: 4<br>2. Move to the next digit and subtract it: 4 - 5 = -1<br>Since the result is not divisible by 11, the original integer (154) is not divisible by 11.<br>This method is a simple and efficient way to check if an integer is divisible by 11 without performing the full division. **[Correct]**
13	To check if an integer is divisible by 13, you can use the following method:<br>1. Take the last digit of the integer.<br>2. Multiply this last digit by 4.<br>3. Add the result from step 2 to the remaining part of the integer after removing the last digit.<br>4. If the resulting number is divisible by 13 (including if it's 0), then the original integer is also divisible by 13.<br>Let's illustrate this method with an example:<br>Let's check if the integer 156 is divisible by 13:<br>1. Last digit: 6<br>2. Multiply last digit by 4: 6 * 4 = 24<br>3. Remaining part of the integer after removing the last digit: 15<br>4. Add the result from step 2 to the remaining part: 15 + 24 = 39<br>Since 39 is divisible by 13 (13 * 3 = 39), the original integer (156) is also divisible by 13.<br>Let's try another example with the integer 289:<br>1. Last digit: 9<br>2. Multiply last digit by 4: 9 * 4 = 36<br>3. Remaining part of the integer after removing the last digit: 28<br>4. Add the result from step 2 to the remaining part: 28 + 36 = 64<br>Since 64 is not divisible by 13, the original integer (289) is not divisible by 13.<br>This method is a simple and efficient way to check if an integer is divisible by 13 without performing the full division. **[Correct]**

Table S2. Response given by ChatGPT on the question "*How to check if an integer can be divided by* N" for $N = 11$ and 13.

Prime N	Solution by ChatGPT on divisibility by N
17	To check if an integer is divisible by 17, you can use the following method:<br>1. Take the last digit of the integer and multiply it by 5.<br>2. Subtract the result from step 1 from the remaining part of the integer (excluding the last digit).<br>3. If the resulting number is divisible by 17 (including if it's 0), then the original integer is also divisible by 17.<br>Let's illustrate this method with an example: Let's check if the integer 272 is divisible by 17:<br>1. Last digit: 2<br>2. Multiply last digit by 5: 2 * 5 = 10<br>3. Remaining part of the integer after removing the last digit: 27<br>4. Subtract the result from step 2 from the remaining part: 27 - 10 = 17<br>Since 17 is divisible by 17 (17 * 1 = 17), the original integer (272) is also divisible by 17. Let's try another example with the integer 357:<br>1. Last digit: 7<br>2. Multiply last digit by 5: 7 * 5 = 35<br>3. Remaining part of the integer after removing the last digit: 35<br>4. Subtract the result from step 2 from the remaining part: 35 - 35 = 0<br>Since 0 is divisible by 17, the original integer (357) is also divisible by 17. **[Correct]**
19	To check if an integer is divisible by 19, you can use the following method:<br>1. Take the last digit of the integer.<br>2. Multiply this last digit by 2.<br>3. Subtract the result from step 2 from the remaining part of the integer (excluding the last digit).<br>4. If the resulting number is divisible by 19 (including if it's 0), then the original integer is also divisible by 19.<br>Let's illustrate this method with an example: Let's check if the integer 1148 is divisible by 19:<br>1. Last digit: 8<br>2. Multiply last digit by 2: 8 * 2 = 16<br>3. Remaining part of the integer after removing the last digit: 114<br>4. Subtract the result from step 2 from the remaining part: 114 - 16 = 98<br>Since 98 is divisible by 19 (19 * 5 = 95), the original integer (1148) is also divisible by 19. Let's try another example with the integer 256:<br>1. Last digit: 6<br>2. Multiply last digit by 2: 6 * 2 = 12<br>3. Remaining part of the integer after removing the last digit: 25<br>4. Subtract the result from step 2 from the remaining part: 25 - 12 = 13<br>Since 13 is not divisible by 19, the original integer (256) is not divisible by 19. **[Wrong]**

Table S3. Response given by ChatGPT on the question "*How to check if an integer can be divided by* N" for $N = 17$ and 19. For $N = 19$, the correct answer is that one need to *add* (not minus) the two times the last digit to the remaining part of the integer.

<table>
<tr><th>Prime
N</th><th>Solution by ChatGPT on divisibility by N</th></tr>
<tr><td>23</td><td>

To check if an integer can be divided by 23, you can use the modulo operation (%), which gives you the remainder when one number is divided by another. If the remainder is 0, then the integer is divisible by 23. Here's how you can do it in various programming languages:

1. Python:

```
num = int(input("Enter an integer: "))
if num % 23 == 0:
    print(f"{num} is divisible by 23.")
else:
    print(f"{num} is not divisible by 23.")
```

2. JavaScript:

```
const num = parseInt(prompt("Enter an integer: "));

if (num % 23 === 0) {
    console.log(‘${num} is divisible by 23.‘);
} else {
    console.log(‘${num} is not divisible by 23.‘);
}
```

3. C++:

```
#include <iostream>
using namespace std;

int main() {
    int num;
    cout << "Enter an integer: ";
    cin >> num;

    if (num % 23 == 0) {
        cout << num << " is divisible by 23." << endl;
    } else {
        cout << num << " is not divisible by 23." << endl;
    }

    return 0;
}
```

As with the previous example, replace the input mechanism with the one suitable for your program. The key point is using the modulo operator (%) to check if the remainder is zero when divided by 29. **[Correct]**

</td></tr>
</table>

Table S4. Response given by ChatGPT on the question "*How to check if an integer can be divided by* 23".

Prime N	Solution by ChatGPT on divisibility by N
29	To check if an integer can be divided by 29, you can follow a similar approach as before and use the modulo operation ($\%$). If the remainder is 0, then the integer is divisible by 29. Here's how you can do it in various programming languages:<br><br>1. Python:<br>`num = int(input("Enter an integer: "))`<br>`if num % 29 == 0:`<br>`    print(f"{num} is divisible by 29.")`<br>`else:`<br>`    print(f"{num} is not divisible by 29.")`<br><br>2. JavaScript:<br>`const num = parseInt(prompt("Enter an integer: "));`<br><br>`if (num % 29 === 0) {`<br>`    console.log(‘${num} is divisible by 29.‘);`<br>`} else {`<br>`    console.log(‘${num} is not divisible by 29.‘);`<br>`}`<br><br>3. C++:<br>`#include <iostream>`<br>`using namespace std;`<br><br>`int main() {`<br>`    int num;`<br>`    cout << "Enter an integer: ";`<br>`    cin >> num;`<br><br>`    if (num % 29 == 0) {`<br>`        cout << num << " is divisible by 29." << endl;`<br>`    } else {`<br>`        cout << num << " is not divisible by 29." << endl;`<br>`    }`<br><br>`    return 0;`<br>`}`<br><br>Replace the input mechanism with the one suitable for your program. The key point is using the modulo operator ($\%$) to check if the remainder is zero when divided by 23. **[Correct]**

Table S5. Response given by ChatGPT on the question "*How to check if an integer can be divided by* 29".

<table>
<tr><th>Prime N</th><th>Solution by ChatGPT on divisibility by N</th></tr>
<tr><td>31</td><td>

To check if an integer is divisible by 31, you can apply the modulo operation (%). If the remainder is 0, then the integer is divisible by 31. Here's how you can do this in different programming languages:

1. Python:

```
num = int(input("Enter an integer: "))
if num % 31 == 0:
    print(f"{num} is divisible by 31.")
else:
    print(f"{num} is not divisible by 31.")
```

2. JavaScript:

```
const num = parseInt(prompt("Enter an integer: "));

if (num % 31 === 0) {
    console.log(`${num} is divisible by 31.`);
} else {
    console.log(`${num} is not divisible by 31.`);
}
```

3. C++:

```
#include <iostream>
using namespace std;

int main() {
    int num;
    cout << "Enter an integer: ";
    cin >> num;

    if (num % 31 == 0) {
        cout << num << " is divisible by 31." << endl;
    } else {
        cout << num << " is not divisible by 31." << endl;
    }

    return 0;
}
```

Replace the input mechanism with the appropriate method for your program. The essential concept here is to use the modulo operator (%) to verify if the remainder is zero when the integer is divided by 31. **[Correct]**

</td></tr>
</table>

Table S6. Response given by ChatGPT on the question "*How to check if an integer can be divided by* 31".
