# Supplementary material for: Classification of integers based on residue classes via modern deep learning algorithms
Source: Patterns (N Y). 2023 Oct 12;4(12):100860. doi: 10.1016/j.patter.2023.100860 (PMC10724366; doi:10.1016/j.patter.2023.100860)
Supplement: Document S2. Article plus supplemental information [file mmc2.pdf]

Article

Patterns

Classification of integers based on residue classes via modern deep learning algorithms

Highlights

- This study underscores the enduring significance of feature engineering
- AutoML and LLMs cannot classify prime numbers without optimal features
- Fourier series regression can effectively solve this problem
- The effectiveness of integer classification hinges on the features provided

Authors

Da Wu, Jingye Yang,
Mian Umair Ahsan, Kai Wang

Correspondence

wangk@chop.edu

In brief

This research delves into the importance of feature engineering in machine learning. Feature engineering is like crafting the right tools for computers to learn and make sense of data. This paper focuses on predicting the residues of dividing a whole number by a small prime number such as 2 and 3. Even in an age of sophisticated automated machine learning tools and advanced large language models, the art of feature engineering remains crucial.

Wu et al., 2023, Patterns *4*, 100860
December 8, 2023 © 2023 The Author(s).
https://doi.org/10.1016/j.patter.2023.100860

CellPress

Patterns

CellPress
OPEN ACCESS

Article

Classification of integers based on residue classes via modern deep learning algorithms

Da Wu,[1] Jingye Yang,[1] Mian Umair Ahsan,[2] and Kai Wang[2,3,*]
[1]Department of Mathematics, University of Pennsylvania, Philadelphia, PA 19104, USA
[2]Department of Pathology, Children's Hospital of Philadelpha, University of Pennsylvania, Philadelphia, PA 19104, USA
[3]Lead contact
*Correspondence: wangk@chop.edu
https://doi.org/10.1016/j.patter.2023.100860

THE BIGGER PICTURE Machine learning heavily relies on feature engineering. We tested multiple deep learning architectures with different feature engineering techniques by considering a fundamental problem of classifying integers. We also tested automated machine learning (AutoML) pipelines developed by big tech companies. Our findings suggest that the quality of features provided plays a crucial role in the effectiveness of classification. Moreover, we evaluated prompt-based learning approaches using large language models (LLMs) and identified their shortcomings. As a promising alternative, we introduced a method that utilizes linear regression on Fourier series basis vectors, which proved to be effective for the general case. Therefore, feature engineering remains an important task to improve performance, increase interpretability, and reduce complexity of machine learning models, even in the era of AutoML and LLMs.

1 2 3 4 5 **Proof-of-Concept:** Data science output has been formulated, implemented, and tested for one domain/problem

SUMMARY

Judging whether an integer can be divided by prime numbers such as 2 or 3 may appear trivial to human beings, but it can be less straightforward for computers. Here, we tested multiple deep learning architectures and feature engineering approaches to classifying integers based on their residues when divided by small prime numbers. We found that the ability of classification critically depends on the feature space. We also evaluated automated machine learning (AutoML) platforms from Amazon, Google, and Microsoft and found that, without appropriately engineered features, they failed on this task. Furthermore, we introduced a method that utilizes linear regression on Fourier series basis vectors and demonstrated its effectiveness. Finally, we evaluated large language models (LLMs) such as GPT-4, GPT-J, LLaMA, and Falcon, and we demonstrated their failures. In conclusion, feature engineering remains an important task to improve performance and increase interpretability of machine learning models, even in the era of AutoML and LLMs.

INTRODUCTION

The task of determining residue class when dividing a given integer, such as 74 or 243,589, by a prime number, such as 2 or 3, remains an interesting and practical problem. In its simplest form, distinguishing whether an integer is odd or even is straightforward for humans. Merely examining the unit digit is sufficient: if it belongs to the set $\{0,2,4,6,8\}$, the number is even; otherwise, it is odd.

On the other hand, classifying integers based on their residues when divided by 3 poses a slightly more difficult challenge. It is well-known that an integer is divisible by 3 if and only if the sum of its numerical digits is divisible by 3. For example, the number 123 is divisible by 3 because $1+2+3 = 6$, which is divisible by 3. Conversely, 59 is not divisible by 3 as the sum of its digits, $5+9 = 14$, is not divisible by 3. Drawing upon this rule, a simple algorithm can be swiftly devised for humans to classify integers based on their residues modulo 3. When confronted with an arbitrary integer n, one can first check if it is divisible by 3. If it is, the classification is complete. If not, one can try $n+1$ or $n-1$. If either of these numbers is divisible by 3, the process stops. Otherwise, one can try $n+2$ or $n-2$. At this stage, termination is necessary as there are only three possible residue classes modulo 3.

This is an open access article under the CC BY license (http://creativecommons.org/licenses/by/4.0/).

Nevertheless, for both cases discussed above, our reliance on mathematical knowledge to design the learning algorithms is absolute. Although no algorithm can achieve satisfactory performance on all possible scenarios based on "no free lunch theorem,"[1] automated machine learning (AutoML) has attracted significant attention and demonstrated success in many domain-specific problems.[2–4] Multiple academic and commercial implementations of AutoML are now available to help users select the best-performing model for a specific problem. Furthermore, a number of large language models (LLMs) have been developed and popularized,[5] with one well-known example being ChatGPT—a powerful chatbot based on LLMs developed by OpenAI.[6,7] These LLMs showed special abilities that are not present in small-scale language models: for example, in addition to memorizing knowledge, LLMs exhibit reasoning abilities when they are sufficiently large.[8–11] Despite these recent development in the machine learning space, the question of whether there exists a systematic approach for Turing machines[12] to autonomously discern patterns from training data and effectively address the integer classification problem appears to be an intriguing and often overlooked issue. This serves as the central problem of our investigation in the current study.

RESULTS

Problem setups

As mentioned above, we are interested in the problem of classifying integers based on their residues mod p. Due to practical considerations such as representations and memory limitations, we restrict our sample space to non-negative integers up to $2^{32}-1$. For instance, when $p = 2$, our parameter space $\mathcal{X}_2 = \mathbb{Z}\cap[0, 2^{32}-1]$ and our label space $\mathcal{Y}_2 = \{0,1\}$. Given a set of training data $S_2 = \{(x_1, y_1), \ldots, (x_n, y_n), y_i \equiv x_i \bmod 2\} \in (\mathcal{X}_2\times\mathcal{Y}_2)^n$, we want to train a classifier $h_{S_2}: \mathcal{X}_2 \mapsto \mathcal{Y}_2$ to predict whether an "unseen" integer is odd or even. In this case, we have a binary classification problem. For general p, the parameter space $\mathcal{X}_p = \mathbb{Z}\cap[0, 2^{32}-1]$ and the label space $\mathcal{Y}_p = \{0, 1, \ldots, p-1\}$. The training set $S_p = \{(x_1, y_1)\ldots,(x_n, y_n): y_i \equiv x_i \bmod p\}$ is sampled from $(\mathcal{X}_p\times\mathcal{Y}_p)^n$, and we hope to build an effective classifier $h_{S_p}: \mathcal{X}_p \mapsto \mathcal{Y}_p$ to classify integers based on their residues mod p. This time we have a multi-class classification problem.

We primarily focus on the cases when $p = 2$ and 3 but also extend some of the analysis to some other small prime numbers, e.g., $p = 7$.

Preparation of datasets

We uniformly sample integers within the range of $[0, 2^{32}-1]$. The specific size of the datasets may vary in different cases and will be specified later. In addition, we also consider the following feature engineering approaches on non-negative integers:

- Binary representation: For instance, 4 is equal to $[0, \ldots, 0, 1, 0, 0]$, 2 is equal to $[0, \ldots, 0, 0, 1, 0]$, and 5 is equal to $[0, \ldots, 1, 0, 1]$.
- Base-three representation: For example, 3 is equal to $[0, \ldots, 0, 1, 0]$, and 6 is equal to $0[0, \ldots, 0, 2, 0]$.
- One-gram encoding: We separate the integer into a vector of numerical digits. For instance, 123 will become $[0, \ldots, 0, 1, 2, 3]$.
- Two-gram encoding: We group two consecutive numerical digits together (with overlap) to form our feature vector. For instance, 1234 will become $[[0,0], \ldots, [0,0], [0,1], [1,2], [2,3], [3,4]]$.
- Three-gram encoding: We group three consecutive numerical digits together (with overlap). For instance, under three-gram encoding, 1234 will become $[[0,0,0], \ldots, [0,0,0], [0,0,1], [0,1,2], [1,2,3], [2,3,4]]$.
- One-gram & two-gram encoding combined: It is the union of one-gram and two-gram encoding.
- One-gram & two-gram & three-gram encoding combined: It is the union of one-gram, two-gram, and three-gram encoding.

All the above feature engineering processes will be tested on both mod 2 and mod 3 cases. In addition to those mentioned above, given the problem nature of mod 3, we also try the following two feature engineering processes:

- One-gram encoding + its sum: In addition to one-gram encoding, we also add sum of all of its numerical digits. For instance, 1234 will become $[0, \ldots, 0, 1, 2, 3, 4, 10]$.
- One-gram encoding + (its sum %3): In this case, 1234 will become $[0, \ldots, 0, 1, 2, 3, 4, 10\%3] = [0, \ldots, 0, 1, 2, 3, 4, 1]$.

Since we ran experiments in Python, we adopt conventions of Python: The $[\ldots]$ means list and the $[[\ldots], \ldots, [\ldots]]$ means nested list (a.k.a. matrix). We also pad additional zeros or zero lists $[0, \ldots, 0]$ on the left to make sure that each feature vector has the same dimension. By doing this, we can convert list-like objects into tensor forms so that we can put them into tensorflow neural networks.

Results from deep neutral networks (DNN)

We first tested on *artificial neural networks* (ANNs). The ANN considered here has 64 neurons in the first layer and 32 neurons in the second layer. The dimensions of the input and output layer depend on the feature engineering process and the number of training labels, respectively. The activation functions are the classical ReLU functions, except that for the output layer, we use the sigmoid function. Besides the classical ANNs, we also tested on a *convolutional neural network* (CNN)[13,14] and *recurrent neural network* (RNN).[15,16] The CNN considered here has two 1D convolution layers, two max pooling layers, and two dense layers with ReLU activation functions. The RNN considered here has a single LSTM layer and a dense layer with sigmoid activation functions. Finally, we tested on *bidirectional encoder representations from transformers* (BERT),[17] which is a family of masked language models introduced by Google in 2018. The ANN, CNN, and RNN architectures considered in this study are illustrated in Figures 1, 2, and 3, respectively.

We first discuss the mod 2 case. As listed in Table 1, ANN on raw data only gives an accuracy of ~ 0.5. It is not surprising that after converting to binary representations, accuracy of 1.000 can be achieved since we have done mod 2 operations already during binary transformations. The one, two, and three-gram encodings (or their combined versions) do give reasonable hints to the algorithms, and they significantly improve the accuracy to ~ 0.8. The BERT model in the end can also achieve an accuracy of 1.000 due to extensive pre-training processes and the state-of-the-art transformer architecture.

CellPress
OPEN ACCESS

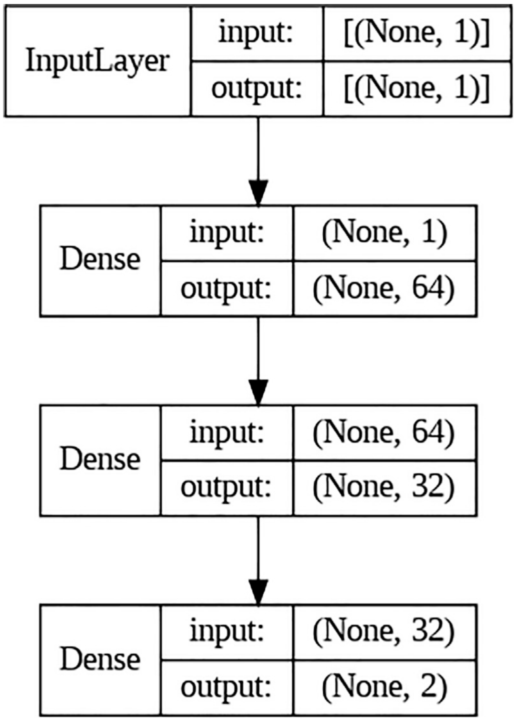

Figure 1. The ANN that we used for training raw data in Subsection "Results from deep neural networks (DNN)."

In the mod 3 case, it is expected that base-three representations can achieve an accuracy of 1.000 even under simple network architectures for the same reason as binary representations. The "one-gram + (its sum %3)" encoding can achieve accuracy of >0.9 in all networks tested since it essentially told algorithms everything about the mod 3 divisibility rule.

It is also interesting to see that BERT can achieve an accuracy of 1.000 from "one-gram + its sum" encoding, whereas it can only have an accuracy of ~ 0.33 from "one-gram" encoding. In other words, the hint of summing up digits works well with BERT but poorly with other types of deep neural networks. BERT is the only algorithm here that can achieve an accuracy of 1.000 with "one-gram + its sum" encoding in mod 3 case. The key difference between BERT and other neural networks is that in BERT, all the possible sums of the digits (99 potential choices of sums assuming there are 10 digits maximum when we restricted input space to $2^{32}-1$) are embedded into algorithms with length 768 each, and therefore, we have 768×99 more features in BERT instead of having only one additional feature (sum of digits) in other neural networks. Due to large amounts of pre-training data and better embedding techniques, these 768×99 additional features have already been "seen and learned" by BERT, and this helps detect the recursive nature of mod 3 problem. On the contrary, in other neural networks, the sum of digits could be large and therefore completely unseen during training, which leads to inaccuracy.

We found that within Table 1 and Table 2, certain outcomes are distinctly surpassing what would be expected from mere random guessing. For instance, in Table 1, ANN (one-gram) attains an accuracy of 0.864, and ANN (two-gram) achieves an accuracy of 0.778. In all these instances, we believe that further

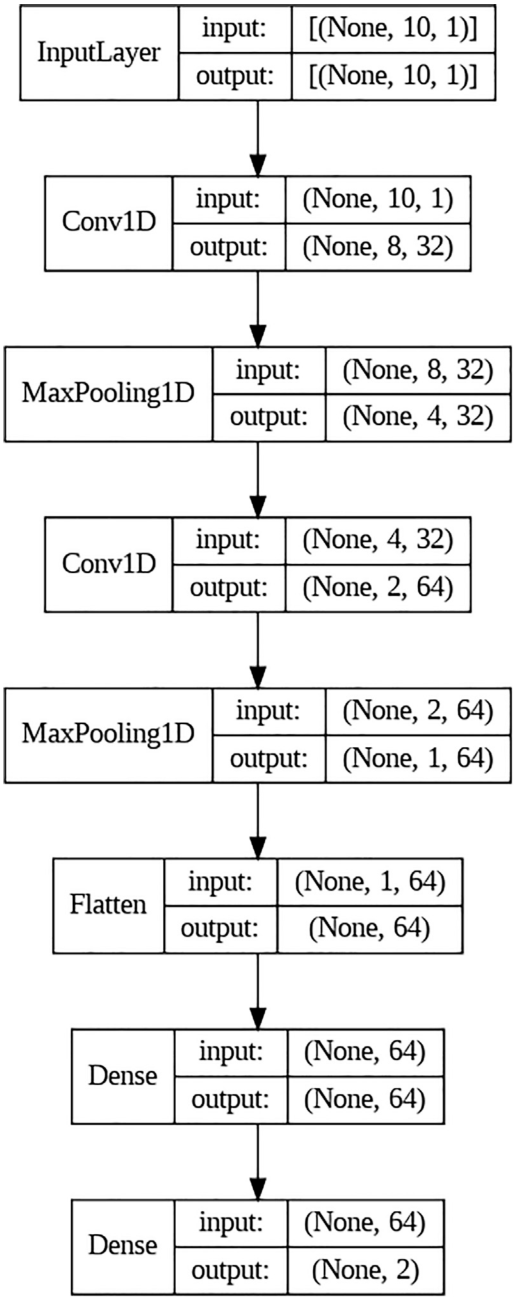

Figure 2. The CNN that we used for training data after one-gram encoding in Subsection "Results from deep neural networks (DNN)."

escalating complexities of the model in a visible scale, as evidenced by our experimentation, could potentially drive these outcomes to a perfect score of 1.000. In other words, these cases enjoy the so-called *scaling property*.[18,19] This fact can also be confirmed during our experimentation on AutoML platforms later. However, in our experiments later, different classes of models were selected and employed, all of which were tree-based algorithms with specific forms of regularization.

It is important to note, however, that this phenomenon is not replicated across all the other scenarios. For those cases with

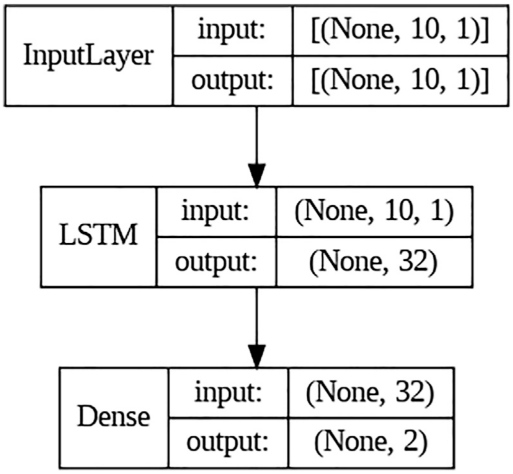

Figure 3. The RNN that we used for training data after one-gram encoding in Subsection "Results from deep neural networks (DNN)."

outcomes equivalent to random guessing (that is, an accuracy of ~ 0.5 for modulo 2 and ~ 0.33 for modulo 3), even though the well-regarded *Universal Approximation Theorem*[20] theoretically assures us of the possibility to perfectly fit training data, it is impractical for individuals to identify the optimal candidates.

Results from AutoML

We also tested on commonly used AutoML platforms developed by Google,[21] Microsoft,[22] and Amazon.[23] To reduce the computing time and save computing power, we uniformly sample 30, 000 non-negative integers from $[0, 2^{32} - 1]$.

As can be seen in Table 3 and Table 4, the pre-installed feature engineering algorithms in AutoML pipelines are not effective at all with raw data. This raises an alert that blindly throwing data into AutoML platforms without any feature engineering has a certain (sometimes very high) level of risks; although these AutoML products are extremely powerful, carefully designed, and updated constantly by ML experts, they cannot guarantee to deliver an effective model autonomously. It is crucial to apply domain knowledge to transform data before training.

In the case of mod 3, as presented in Table 4, breaking down and/or combining digits without summing them up are not effective. However, summing up all the digits in addition to one-gram encoding can help AutoML platforms deliver classifiers with an accuracy of 1.000. Note that results of AutoML are reported for the best possible model from multi-angle considerations (e.g., complexity, interpretations, etc.), and it appears that these AutoML platforms heavily prefer tree-based algorithms, even though deep learning algorithms were also considered.

Results from Fourier series regression

Next, we proposed a method using Fourier series regressions and tested on mod 3 and mod 7 problems. In both cases, an accuracy of 1.000 can be achieved. This approach proves effective for handling all values of p with a minimal number of training samples, provided that the training dataset size significantly exceeds the value of p. To demonstrate this point, this section contains outcomes of both modulo 3 and modulo 7 problems, based on a limited dataset of only 200 samples, divided into 150 for training and validation (135 for training and 15 for validation) and 50 for testing. An accuracy of 1.000 can already be attained for any given test set. Augmenting the sample size by a factor of 10 or 100 does not exhibit any noticeable impact on accuracy and only imparts minimal influence on regression estimations.

Here, our total dataset consists of 200 uniform samples from $[0, 2^{32} - 1]$, among which 150 samples are used to estimate regression coefficients, and the remaining 50 ones are used for testing purposes. Fix integer $p = 3$ or 7. Suppose $X = [x_1, \ldots, x_n]^T$ is our training set, and let $Y_p = [y_1, \ldots, y_n]^T$ be the vector of training labels, i.e., $y_i \equiv x_i \bmod p$, where $y_i \in \{0, 1 \ldots, p-1\}$ and $p \in \{3, 7\}$. For each $j = 1, 2, \ldots, \lfloor p/2 \rfloor$, let

$$\sin\left(\frac{2\pi j}{p}X\right) = \left[\sin\left(\frac{2\pi j}{p}\cdot x_1\right), \ldots, \sin\left(\frac{2\pi j}{p}\cdot x_n\right)\right]^T \quad \text{(Equation 1)}$$

and

$$\cos\left(\frac{2\pi j}{p}X\right) = \left[\cos\left(\frac{2\pi j}{p}\cdot x_1\right), \ldots, \cos\left(\frac{2\pi j}{p}\cdot x_n\right)\right]^T \quad \text{(Equation 2)}$$

be vectors of Fourier series basis, and we consider the following linear regression:

$$Y_p = \gamma + \sum_{j=1}^{\lfloor p/2 \rfloor}\left(\alpha_j \sin\left(\frac{2\pi j}{p}X\right) + \beta_j \cos\left(\frac{2\pi j}{p}X\right)\right) + \varepsilon, \quad \text{(Equation 3)}$$

where ε denotes the vector of standard Gaussian noise. All the coefficients in (Equation 3) are estimated by classical *ordinary least square* (OLS) regression.

Generally speaking, the linear regression model is not a good candidate for classification problems due to a number of reasons, one of them being that the output values are continuous instead of categorical. However, in our case, due to the large number of training samples, predicted values on the testing set are very close to integers (see Table 5 and Table 6) so that we can round them to the nearest integer if their distance is within 10^{-5}.

Regression estimates of mod 3

By using `LinearRegression` package in `sklearn` of Python, we have the following regression coefficients estimate:

$$\widehat{Y_3}(X) = 0.9999999556293363 + (-0.57735)\cdot\sin\left(\frac{2\pi}{3}X\right) + (-1.00000)\cdot\cos\left(\frac{2\pi}{3}X\right), \quad \text{(Equation 4)}$$

with R^2 value being 0.9999999999999453 and all coefficients being statistically significant. The R^2 value is computed under train-validation split ratio 0.1. The plot of regression estimate (Equation 4) and its values at integer points, covering three periods, are recorded in Figure 4 and Table 5 respectively. In the mod 3 case, accuracy of 1.000 can be achieved. We also want to emphasize that the joint presence of both sines and cosines

Table 1. Results on mod 2 via deep neural networks

Classification on divisibility by 2 via deep neural networks		
Algorithms (feature engineering)	Mean	Standard Deviation
Artificial neutral network (ANN)	0.501	0.003
ANN (binary representation)	1.000	0.000
ANN (base-three representation)	0.537	0.002
ANN (one-gram)	0.864	0.024
ANN (two-gram)	0.778	0.048
ANN (three-gram)	0.799	0.002
ANN (one & two-gram combined)	0.786	0.028
ANN (one & two & three-gram combined)	0.781	0.023
Convolutional neural network (one-gram)	0.921	0.042
Recurrent neural network (one-gram)	0.740	0.026
Bidirectional encoder representations from transformers (BERT) (one-gram)	1.000	0.000

Means and standard deviations are calculated with three uniformly sampled, equal-size training sets.

is needed, and if we, for instance, remove all the cosines and only keep the sines in (Equation 3), then R^2-value is only 0.2533, and accuracy is 0.3206 with the same dataset. On the contrary, we can also add more pairs of sine and cosine, e.g., $j = 1,\ldots p - 1$, in our Fourier series basis (Equation 1) and (Equation 2). This will also give us a satisfying linear regression estimate (accuracy 1.000) with "less overshoot and undershoot" compared to (Equation 3). However, the regression table will give the potential warning of *multicollinearity*,[24–26] indicating that too many features were added, which may cause instability of regression coefficients estimates in some large p cases. Therefore, our linear regression model (Equation 3) is *optimal*.

Regression estimates of mod 7

In the case of mod 7, we have the following coefficients estimates:

$$\begin{aligned}\widehat{Y_7}(X) \;=\; & 3.0000000310122816 + (-2.076521)\cdot\sin\left(\frac{2\pi}{7}X\right) \\ & + (-1.000000)\cdot\cos\left(\frac{2\pi}{7}X\right) \\ & + (-0.797473)\cdot\sin\left(\frac{4\pi}{7}X\right) + (-1.000000)\cdot\cos\left(\frac{4\pi}{7}X\right) \\ & + (-0.228243)\cdot\sin\left(\frac{6\pi}{7}X\right) + (-1.000000)\cdot\cos\left(\frac{6\pi}{7}X\right),\end{aligned}$$

(Equation 5)

with R^2-value being 0.9999999999999203 and all coefficients being statistically significant. Again, accuracy of 1.000 can be achieved on the testing set. The plot of regression estimate (Equation 5) and its values at integer points, covering two periods, are recorded in Figure 5 and Table 6, respectively.

Results from large language models

Next, we conducted tests to assess the proficiency of open-source LLMs, specifically GPT-J-6B,[27,28] LLaMA-7B,[29] and Falcon-40B,[30] in their understanding of divisibility rules. Specifically, for each prime p up to 31, we tried the following two prompts:

- P1: "There are various mathematical rules to check if an integer is divisible by p, for instance"
- P2: "How to check if an integer is divisible by p, for instance we can"

We utilized these two prompts to better steer the open-source models toward producing mathematical answers as opposed to algorithmic ones. Additionally, we included "for instance …" since open-source models are more suitable for completing sentences rather than providing direct answers to questions.

The comprehensive responses are presented in the supplemental experimental procedures. Within this context, we manually assess the accuracy and informativeness of responses generated by those models. The elaborated outcomes are recorded in Table 7. For the "Correct" notation, we refer to the *mathematical accuracy of the provided answers*. For the "Informative" notation, we assess *whether the models effectively articulate the divisibility rule in a lucid and cohesive manner*. As an example, when the model attempts to employ the modulo operator (%)—while its mathematical accuracy is unquestionable—it ultimately lacks any informative value. The data presented in Table 7 demonstrates the inadequacy of these open-source LLMs for primes beginning at 7. Even when dealing with smaller primes, they consistently produce incorrect and uninformative replies.

Furthermore, we assessed ChatGPT's[31] closed-source implementation utilizing reinforcement learning on the knowledge of divisibility rules. All outcomes can be entirely replicated, and to ensure alignment with open-source models, we also incorporate ChatGPT's evaluations in Table 7. Specifically, we used the following prompt:

- P: "How to check if an integer is divisible by p?".

Regarding ChatGPT, utilizing the aforementioned prompts (P1 and P2) is unnecessary, as it possesses a strong capability to comprehend the genuine intention behind the prompt P. The comprehensive results are furnished in Tables S1–S6. Notable enhancements are observed compared to open-source LLMs. Nevertheless, beginning at 23, ChatGPT resorts to using the modulo operator % to tackle the issue, resulting in completely

Table 2. Results on mod 3 via deep neural networks

Classification on divisibility by 3 via deep neural networks		
Algorithms (feature engineering)	Mean	Standard Deviation
Artificial neutral network (ANN)	0.334	0.002
ANN (binary representation)	0.396	0.002
ANN (base-three representation)	1.000	0.000
ANN (one-gram)	0.343	0.003
ANN (one-gram + its sum)	0.335	0.002
ANN (one-gram + (its sum %3))	1.000	0.000
ANN (two-gram)	0.334	0.002
ANN (three-gram)	0.333	0.001
ANN (one & two-gram combined)	0.332	0.004
ANN (one & two & three-gram combined)	0.334	0.007
Convolutional neural network (CNN) (one-gram)	0.340	0.002
CNN (one-gram + its sum)	0.330	0.002
CNN (one-gram + (its sum %3))	0.936	0.001
Recurrent neural network (RNN) (one-gram)	0.330	0.003
RNN (one-gram + its sum)	0.333	0.001
RNN (one-gram + (its sum %3))	0.934	0.001
Bidirectional encoder representations from transformers (BERT) (one-gram)	0.336	0.005
BERT (one-gram + its sum)	1.000	0.000
BERT (one-gram + (its sum %3))	1.000	0.000

Means and standard deviations are calculated with three uniformly sampled, equal-size training sets.

uninformative answers. As a result, its adequacy for larger primes still lags behind.

Finally, we tested the latest GPT-4 with code interpreter (distinct from the aforementioned ChatGPT) on its capabilities of designing deep neural networks to address the integer divisibility problems. Note that as of now, access to GPT-4 is exclusively offered to subscribers of ChatGPT plus. We showcased its code in the supplemental experimental procedures. While correct, it does not possess the capability to formulate an efficient algorithm, which encompasses identifying appropriate feature engineering techniques and selecting the optimal ML/DL algorithms.

Hence, it can be deduced that current state-of-the-art LLMs do not possess the required capability to offer dependable and accurate information regarding divisibility rules.

DISCUSSION

In our current study, we conducted extensive experiments to delve into classifications of large finite integers, specifically those up to $2^{32} - 1$, based on their residues when divided by small prime numbers. Our investigation involved testing various deep neural network architectures and employing diverse feature engineering approaches. The obtained results were both intuitive and straightforward to interpret.

An important observation that emerged throughout our analysis is that, regardless of complexities of network structures or specific neural network frameworks used, the performance of our classification task was highly reliant on the feature space provided to deep learning models. This discovery remained consistent not only for neural network architectures but also when evaluating AutoML pipelines.

Feature engineering is often a challenging and non-intuitive process in practical scenarios, requiring extensive trial-and-error iterations.[32–34] In addition to directly engineering features on training samples, there are other avenues where domain expertise can be leveraged to enhance the quality of classifiers. Inspired by the *recurring pattern* exhibited by the modulus function, we devised a simple method that utilizes linear regression with Fourier series basis vectors to capture and understand its periodic behavior. This method exhibited exceptional performance, achieving a perfect accuracy of 1.000 for all modulus p problems, even when p is not necessarily prime. Furthermore, our proposed approach offers advantages such as minimal training size, reduced time complexity, and improved interpretability of the model, outperforming all the other state-of-the-art ML/DL models in these aspects.

To expand our evaluation further, we also examined the performance of GPT-J-6B, LLaMA-7B, and Falcon-40B concerning divisibility by primes up to 31. Regrettably, our investigation uncovered that these open-source LLMs exhibited a tendency to produce inaccurate and uninformative replies even when primes p are small. Additionally, we conducted tests on closed-source ChatGPT and observed a relatively improved performance compared to the aforementioned open-source LLMs. However, it is important to note that ChatGPT still demonstrated some instances of erroneous information, particularly when dealing with larger prime number p. For $p \geq 23$, it cannot provide any informative answers at all. Finally, we tested the latest GPT-4 with code interpreter on its capabilities of designing an effective

Table 3. Results on mod 2 via AutoML

	Microsoft Azure ML		Google Cloud Vertex AI		Amazon Sagemaker	
Feature engineering	Accuracy	Model	Accuracy	Model	Accuracy	Model
No feature engineering	0.504	RandomForest	0.495	N/A	0.508	WeightedEnsemble
Binary representation	1.000	XGBoost	1.000	N/A	1.000	LightGBM
Base-three representation	0.508	RandomForest	0.506	N/A	0.514	WeightedEnsemble
One-gram	1.000	XGBoost	1.000	N/A	1.000	LightGBM
Two-gram	1.000	LightGBM	1.000	N/A	1.000	LightGBM
Three-gram	1.000	XGBoost	1.000	N/A	1.000	LightGBM
One- & two-gram	1.000	XGBoost	1.000	N/A	1.000	LightGBM
One- & two- & three-gram	1.000	RandomForest	1.000	N/A	1.000	LightGBM

For Microsoft and Amazon, we report the best possible model and their respective performance on test. The Google AutoML platform did not report the specific model and only reported testing statistics.

neural network to address the mod p problem and found that it still lacks the competence to do so.

In conclusion, our study emphasizes the ongoing importance of feature engineering in the AutoML and LLM era. We demonstrated that performances of deep learning models are heavily reliant on carefully engineered features. Without appropriate feature engineering, it is impossible to enhance performance merely by increasing complexities of algorithms on a feasible level or enlarging sizes of training datasets; despite the renowned universal approximation theorem[20] offering a theoretical assurance that achieving a perfect fit to the training data is possible, it is impractical for individuals to effectively discover the desired candidates in practice. Moreover, our proposed method employing linear regression on Fourier series basis vectors showcased exceptional accuracy, lower time complexity, and enhanced interpretability. Finally, we caution against relying on LLMs for divisibility by large primes, as our findings indicated that they may provide inaccurate information in these cases.

EXPERIMENTAL PROCEDURES

Resource availability

Lead contact

Further information and requests for data should be directed to and will be fulfilled by the lead contact, Dr. Kai Wang (wangk@chop.edu).

Materials availability

This study did not generate new unique materials.

Data and code availability

Our study did not generate any new data. All the publicly available code for producing training data (uniformly sampled integers), conducting testing using various feature engineering approaches, and implementing distinct deep learning algorithms outlined in Table 1 and Table 2, as well as the Fourier series regression discussed in Section "Results from Fourier series regression," can be accessed at Reference 35.[35]

Deep neutral networks

The ANN considered in this study has 64 neurons in the first layer and 32 neurons in the second layer. The dimensions of the input and output layer depend on what feature engineering process we are using and how many training labels there are. The activation functions are the classical ReLU functions, except that for the output layer, we use the sigmoid function. Notice that this is the only ANN considered in this study, and we will not compare the results with other more complex ANNs. This consideration is due to the facts that (1) the above neural network is sufficient for us to demonstrate the importance of feature engineering, and (2) the more complex networks will be tested on the commonly used AutoML platforms.

Besides the classical ANNs, we also considered CNN and RNN. CNN is a well-known deep learning algorithm inspired by the natural vision perception mechanisms of living creatures. The modern framework of CNN was established by LeCun et al.[36] and later improved.[37] For recent advances in CNN and its applications, see Gu et al.[38] and the references therein. The RNN architecture was mainly designed to overcome the issue of "limited context memory" in Bengio et al.[39]: only a fixed number of previous words can be taken into

Table 4. Results on mod 3 via AutoML

	Microsoft Azure ML		Google Cloud Vertex AI		Amazon Sagemaker	
Feature engineering	Accuracy	Model	Accuracy	Model	Accuracy	Model
No feature engineering	0.357	RandomForest	0.352	N/A	0.345	WeightedEnsemble
Binary representation	0.361	RandomForest	0.342	N/A	0.345	WeightedEnsemble
Base-three representation	1.000	XGBoost	1.000	N/A	1.000	LightGBM
One-gram	0.352	RandomForest	0.352	N/A	0.348	WeightedEnsemble
One-gram + its sum	1.000	XGBoost	1.000	N/A	1.000	LightGBM
One-gram + (its sum %3)	1.000	LightGBM	1.000	N/A	1.000	LightGBM
Two-gram	0.359	RandomForest	0.353	N/A	0.348	WeightedEnsemble
Three-gram	0.346	RandomForest	0.349	N/A	0.351	WeightedEnsemble
One- & two-gram	0.348	RandomForest	0.351	N/A	0.349	WeightedEnsemble
One- & two- & three-gram	0.346	XGBoost	0.341	N/A	0.345	WeightedEnsemble

For Microsoft and Amazon, we report the best possible model and their respective performance on test. The Google AutoML platform did not report the specific model and only reported testing statistics.

Table 5. Values of the regression estimate $\widehat{Y_3}(X)$ (Equation 4) at integer points

X	$\widehat{Y_3}(X)$	X mod 3
0	$-5.473399511402022 \cdot 10^{-14}$	0
1	1.0000002331249993	1
2	1.9999997668748912	2
3	$-5.46229728115577 \cdot 10^{-14}$	0
4	1.0000002331249984	1
5	1.999999766874892	2
6	$-5.440092820663267 \cdot 10^{-14}$	0
7	1.0000002331249984	1
8	1.999999766874892	2
9	$-5.4289905904170155 \cdot 10^{-14}$	0

account to predict the next word. In RNN, the context length was extended to indefinite size, which can handle arbitrary context lengths. See De Mulder et al.[40] for the review on the RNN model and its applications on the statistical language modeling. The CNN we used has two 1D convolution layers, two max pooling layers, and two dense layers with ReLU activation functions. The RNN we used has a single LSTM layer and a dense layer with sigmoid activation functions. We used 10 epochs with a batch size of 32 during training.

Finally, we tested on BERT, which is a family of masked language models introduced by Google in 2018.[17] It is based on the transformer encoders, and it was pre-trained simultaneously on two tasks: *masked language modeling* and *next sentence prediction*. After pre-training, it can be *fine-tuned* on smaller datasets to optimize its performance on specific tasks, including text classifications, language inference, etc. See also Lin et al., Han et al., Khan et al., and Ganesh et al.[41–44] for more discussions on the BERT model and the transformer architecture. Here we tested the BERT model on both mod 2 and 3 problems via the `BertForSequenceClassification` package in Python. We used 10 epochs with a batch size of 64 and a learning rate of 10^{-6}.

Automated machine learning

We considered three commonly used AutoML platforms: Microsoft Azure ML Studio,[22] Google Cloud Vertex AI,[21] and Amazon AWS Sagemaker.[23] All of

Table 6. Values of the regression estimate $\widehat{Y_7}(X)$ (Equation 5) at integer points

X	$\widehat{Y_7}(X)$	X mod 7
0	$3.1012281631603855 \cdot 10^{-8}$	0
1	1.0000009260275013	1
2	1.9999998780188157	2
3	3.000000361534261	3
4	3.9999997004903016	4
5	5.000000184005747	5
6	5.999999135997062	6
7	$3.101228274182688 \cdot 10^{-8}$	0
8	1.000000926027501	1
9	1.9999998780188162	2
10	3.000000361534261	3
11	3.999999700490302	4
12	5.000000184005747	5
13	5.999999135997064	6
14	$3.1012283852049904 \cdot 10^{-8}$	0
15	1.0000009260274998	1
16	1.9999998780188164	2

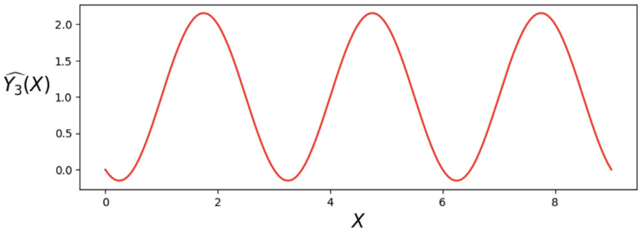

Figure 4. Plot of regression estimate $\widehat{Y}_3(X)$ (Equation 4).

them are easily accessible online with limited free credits. One can upload the datasets in the ".csv" format and pre-specify the target value, type of tasks (classification, regression, NLP, etc.), primary metric of evaluation, etc.

Fourier series regressions

In this section, we described our Fourier series regression method in detail. Fix an integer $p \geq 2$. Let $X = [x_1, \ldots, x_n]^T$ be our training set, and let $Y_p = [y_1, \ldots, y_n]^T$ be the vector of training labels, i.e., $y_i \equiv x_i \bmod p$, where $y_i \in \{0, 1 \ldots, p - 1\}$. For each x_i in X, consider the following Fourier series basis:

$$\left\{ \sin\left(\frac{2\pi j}{p} \cdot x_i\right), \cos\left(\frac{2\pi j}{p} \cdot x_i\right), \text{where } j = 1, 2, \ldots, [p/2] \right\}. \quad \text{(Equation 6)}$$

For each j, let

$$\sin\left(\frac{2\pi j}{p} X\right) = \left[\sin\left(\frac{2\pi j}{p} \cdot_1\right), \ldots, \sin\left(\frac{2\pi j}{p} \cdot x_n\right)\right]^T$$

and

$$\cos\left(\frac{2\pi j}{p} X\right) = \left[\cos\left(\frac{2\pi j}{p} \cdot x_1\right), \ldots, \cos\left(\frac{2\pi j}{p} \cdot x_n\right)\right]^T$$

be the vectors of Fourier series basis, and consider the following linear regression equation:

$$Y_p = \gamma + \sum_{j=1}^{[p/2]} \left(\alpha_j \sin\left(\frac{2\pi j}{p} X\right) + \beta_j \cos\left(\frac{2\pi j}{p} X\right) \right) + \varepsilon, \quad \text{(Equation 7)}$$

where ε denotes the vector of standard Gaussian noise. All the coefficients in (Equation 7) are estimated by the classical OLS method. For prediction, we round the predicted values from linear regression (Equation 7) to the nearest integer if their distance is within 10^{-5}.

Large language models

Finally, we tested the knowledge of GPT-J-6B,[27] LLaMA-7B,[29] and Falcon-40B[45] on divisibility rules of prime numbers. These models are all open-sourced and can be easily implemented using hugging face API.[30,46,47]

For demonstration purposes, we conducted tests on several scenarios where p took on the primes up to 31. For each prime p, we employed the

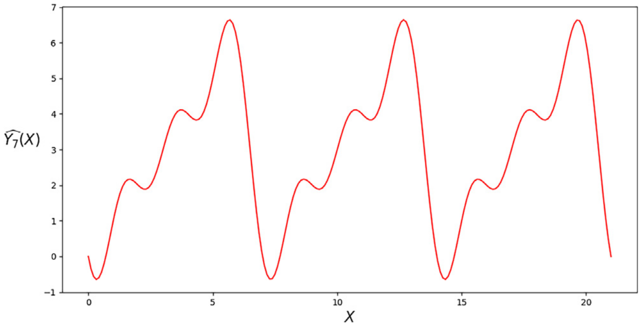

Figure 5. Plot of regression estimate $\widehat{Y}_7(X)$ (Equation 5).

CellPress
OPEN ACCESS

Table 7. The proficiency of LLMs in providing both accurate mathematical solutions and informative explanations regarding divisibility rules by prime numbers up to 31

	GPT-J-6B				LLaMA-7B				Falcon-40B				ChatGPT-175B	
	Correct		Informative		Correct		Informative		Correct		Informative		Correct	Informative
Prime *p*	P1	P2	P1	P2	P1	P2	P1	P2	P1	P2	P1	P2	P	P
2	no	no	yes	no	no	yes	no	no	no	no	no	no	yes	yes
3	no	no	no	no	yes	yes	yes	yes	yes	yes	yes	no	yes	yes
5	no	no	no	no	yes	yes	yes	yes	yes	yes	no	no	yes	yes
7	yes	no	yes	no	no	no	no	no	no	no	no	no	yes	yes
11	no	no	no	no	no	no	no	no	no	no	no	no	yes	yes
13	no	no	no	no	no	no	no	no	no	no	no	no	yes	yes
17	no	no	no	no	no	no	no	no	no	no	no	no	yes	yes
19	no	no	no	no	no	no	no	no	no	no	no	no	no	yes
23	no	no	no	no	no	no	no	no	no	no	no	no	yes	no
29	no	no	no	no	no	no	no	no	no	no	no	no	yes	no
31	no	no	no	no	no	no	no	no	no	no	no	no	yes	no

The detailed responses are documented in Notes S1–S3 and Table S1–S6.

following two prompts for each of these three models, ensuring consistency across the evaluations.

1. "How to check if an integer is divisible by *p*, for instance we can"
2. "There are various mathematical rules to check if an integer is divisible by *p*, for instance"

The maximum token lengths for GPT-J-6B, LLaMA-7B, and Falcon-40B are set to be $100, 160,$ and 200 respectively. For ChatGPT,[31] we used "How to check if an integer is divisible by *p*?" as the prompt. For GPT-4, we used "Can you design a deep learning algorithm to determine if a number can be divided by *p*?"

SUPPLEMENTAL INFORMATION

Supplemental information can be found online at https://doi.org/10.1016/j.patter.2023.100860.

ACKNOWLEDGMENTS

This project originated from a discussion between Umair Ahsan (CHOP), Qian Liu (UNLV), and Kai Wang (CHOP/Penn) during the peak of COVID-19. We are grateful to Dr. Xin Sun (Penn), Dr. Herman Gluck (Penn), and Penn Data Driven Discovery Initiative for their support of pursuing this project. We thank Wang lab members for helpful discussions and comments on the methods and results. The study and open-access charge are supported by the CHOP Research Institute.

AUTHOR CONTRIBUTIONS

Conceptualization, D.W. and K.W.; methodology, D.W., J.Y., M.U.A., and K.W.; investigation, D.W., J.Y., M.U.A., and K.W.; writing—original draft, D.W. and K.W.; writing—review and editing, D.W., J.Y., and K.W.; funding acquisition, K.W.; supervision, K.W.

DECLARATION OF INTERESTS

The authors declare that they have no known competing financial interests or personal relationships that could have appeared to influence the work reported in this paper.

Received: June 18, 2023
Revised: August 11, 2023
Accepted: September 20, 2023
Published: October 12, 2023

REFERENCES

1. Wolpert, D.H., and Macready, W.G. (1997). No free lunch theorems for optimization. IEEE Trans. Evol. Comput. *1*, 67–82.
2. Karmaker ("Santu"), S.K., Hassan, M.M., Smith, M.J., Xu, L., Zhai, C., and Veeramachaneni, K. (2021). AutoML to Date and Beyond: Challenges and Opportunities. ACM Comput. Surv. *54*, 1–36. https://doi.org/10.1145/3470918.
3. He, X., Zhao, K., and Chu, X. (2021). AutoML: A survey of the state-of-the-art. Knowl. Base Syst. *212*, 106622. https://doi.org/10.1016/j.knosys.2020.106622.
4. Truong, A., Walters, A., Goodsitt, J., Hines, K., Bruss, C., and Farivar, R. (2019). Towards Automated Machine Learning: Evaluation and Comparison of AutoML Approaches and Tools. In 2019 IEEE 31st International Conference on Tools with Artificial Intelligence (ICTAI), Los Alamitos, CA, USA, 1471–1479 (IEEE Computer Society). https://doi.org/10.1109/ICTAI.2019.00209.
5. Zhao, W.X., Zhou, K., Li, J., Tang, T., Wang, X., Hou, Y., Min, Y., Zhang, B., Zhang, J., Dong, Z., et al. (2023). A survey of large language models. Preprint at arXiv. https://doi.org/10.48550/arXiv.2303.18223.
6. Brown, T., Mann, B., Ryder, N., Subbiah, M., Kaplan, J.D., Dhariwal, P., Neelakantan, A., Shyam, P., Sastry, G., Askell, A., et al. (2020). Language models are few-shot learners. Adv. Neural Inf. Process. Syst. *33*, 1877–1901.
7. Ouyang, L., Wu, J., Jiang, X., Almeida, D., Wainwright, C., Mishkin, P., Zhang, C., Agarwal, S., Slama, K., Ray, A., et al. (2022). Training language models to follow instructions with human feedback. Adv. Neural Inf. Process. Syst. *35*, 27730–27744.
8. Huang, J., and Chang, K.C.-C. (2022). Towards reasoning in large language models: A survey. Preprint at arXiv. https://doi.org/10.48550/arXiv.2212.10403.
9. Saparov, A., and He, H. (2022). Language models are greedy reasoners: A systematic formal analysis of chain-of-thought. Preprint at arXiv. https://doi.org/10.48550/arXiv.2210.01240.
10. Li, Y., Lin, Z., Zhang, S., Fu, Q., Chen, B., Lou, J.-G., and Chen, W. (2022). On the advance of making language models better reasoners. Preprint at arXiv. https://doi.org/10.48550/arXiv.2206.02336.
11. Creswell, A., Shanahan, M., and Higgins, I. (2022). Selection-inference: Exploiting large language models for interpretable logical reasoning. Preprint at arXiv. https://doi.org/10.48550/arXiv.2205.09712.
12. Turing, A.M. (2009). Computing Machinery and Intelligence (Springer).

13. Li, Z., Liu, F., Yang, W., Peng, S., and Zhou, J. (2021). A survey of convolutional neural networks: analysis, applications, and prospects. IEEE Transact. Neural Networks Learn. Syst. https://doi.org/10.1109/TNNLS.2021.3084827.
14. Alzubaidi, L., Zhang, J., Humaidi, A.J., Al-Dujaili, A., Duan, Y., Al-Shamma, O., Santamaría, J., Fadhel, M.A., Al-Amidie, M., and Farhan, L. (2021). Review of deep learning: Concepts, CNN architectures, challenges, applications, future directions. J. Big Data *8*, 53–74. https://doi.org/10.1186/s40537-021-00444-8.
15. Sherstinsky, A. (2020). Fundamentals of recurrent neural network (RNN) and long short-term memory (LSTM) network. Phys. Nonlinear Phenom. *404*, 132306. https://doi.org/10.1016/j.physd.2019.132306.
16. Yu, Y., Si, X., Hu, C., and Zhang, J. (2019). A Review of Recurrent Neural Networks: LSTM Cells and Network Architectures. Neural Comput. *31*, 1235–1270. https://doi.org/10.1162/neco_a_01199.
17. Devlin, J., Chang, M., Lee, K., and Toutanova, K. (2019). BERT: Pre-training of Deep Bidirectional Transformers for Language Understanding. In Proceedings of the 2019 Conference of the North American Chapter of the Association for Computational Linguistics: Human Language Technologies, NAACL-HLT 2019, *Volume 1*, J. Burstein, C. Doran, and T. Solorio, eds. *(Association for Computational Linguistics), pp. 4171–4186. (Long and Short Papers),. https://doi.org/10.18653/v1/n19-1423*.
18. Cohen, A.-S., Cont, R., Rossier, A., and Xu, R. (2021). Scaling properties of deep residual networks. In International Conference on Machine Learning (PMLR), pp. 2039–2048.
19. Xu, Y., and Zhang, H. (2021). Convergence of deep ReLU networks. Preprint at arXiv. https://doi.org/10.48550/arXiv.2107.12530.
20. Hornik, K., Stinchcombe, M., and White, H. (1989). Multilayer feedforward networks are universal approximators. Neural Network. *2*, 359–366.
21. Google Cloud AutoML (2023). https://cloud.google.com/automl.
22. Microsoft Azure AutoML. (2023). https://azure.microsoft.com/en-us/products/machine-learning/automatedml/.
23. Amazon Sagemaker AutoML. (2023). https://aws.amazon.com/sagemaker/autopilot/.
24. Alin, A. (2010). Multicollinearity. WIREs Computational Stats. *2*, 370–374.
25. Mansfield, E.R., and Helms, B.P. (1982). Detecting multicollinearity. Am. Statistician *36*, 158–160. https://doi.org/10.1080/00031305.1982.10482818.
26. Daoud, J.I. (2017). Multicollinearity and regression analysis. In J. Phys. Conf., *Volume 949* (IOP Publishing), 012009. https://doi.org/10.1088/1742-6596/949/1/012009.
27. Wang, B. (2021). Mesh-Transformer-JAX: Model-Parallel Implementation of Transformer Language Model with JAX. https://github.com/kingoflolz/mesh-transformer-jax.
28. Wang, B., and Komatsuzaki, A. (2021). GPT-J-6B: A 6 Billion Parameter Autoregressive Language Model. https://github.com/kingoflolz/mesh-transformer-jax.
29. Touvron, H., Lavril, T., Izacard, G., Martinet, X., Lachaux, M.-A., Lacroix, T., Rozière, B., Goyal, N., Hambro, E., Azhar, F., et al. (2023). LLaMA: Open and Efficient Foundation Language Models.
30. Huggingface Model Description. (2023). https://huggingface.co/tiiuae/falcon-40b.
31. ChatGPT by OpenAI. (2023). https://openai.com/blog/chatgpt.
32. Heaton, J. (2016). An empirical analysis of feature engineering for predictive modeling. SoutheastCon 2016, *1–6* (IEEE). https://doi.org/10.1109/SECON.2016.7506650.
33. Ozdemir, S., and Susarla, D. (2018). Feature Engineering Made Easy: Identify Unique Features from Your Dataset in Order to Build Powerful Machine Learning Systems (Packt Publishing Ltd).
34. Hermann, K., and Lampinen, A. (2020). What shapes feature representations? exploring datasets, architectures, and training. Adv. Neural Inf. Process. Syst. *33*, 9995–10006.
35. Wu, D. (2023). WGLab/Integer_divsibility: v1.0.1. https://doi.org/10.5281/zenodo.8327655.
36. Lecun, Y., Boser, B., Denker, J., Henderson, D., Howard, R., Hubbard, W., and Jackel, L. (1990). Handwritten digit recognition with a back-propagation network. In Advances in Neural Information Processing Systems (NIPS 1989), *Volume 2*, D. Touretzky, ed. *(Morgan Kaufmann)*.
37. LeCun, Y., Bottou, L., Bengio, Y., and Haffner, P. (1998). Gradient-based learning applied to document recognition. Proc. IEEE *86*, 2278–2324. https://doi.org/10.1109/5.726791.
38. Gu, J., Wang, Z., Kuen, J., Ma, L., Shahroudy, A., Shuai, B., Liu, T., Wang, X., Wang, G., Cai, J., and Chen, T. (2018). Recent Advances in Convolutional Neural Networks. Pattern Recogn. *77*, 354–377. https://doi.org/10.1016/j.patcog.2017.10.013.
39. Bengio, Y., Ducharme, R., Vincent, P., and Janvin, C. (2003). A Neural Probabilistic Language Model. J. Mach. Learn. Res.
40. De Mulder, W., Bethard, S., and Moens, M.-F. (2015). A Survey on the Application of Recurrent Neural Networks to Statistical Language Modeling. Comput. Speech Lang *30*, 61–98. https://doi.org/10.1016/j.csl.2014.09.005.
41. Lin, T., Wang, Y., Liu, X., and Qiu, X. (2022). A survey of transformers. AI Open *3*, 111–132. https://doi.org/10.1016/j.aiopen.2022.10.001.
42. Han, K., Wang, Y., Chen, H., Chen, X., Guo, J., Liu, Z., Tang, Y., Xiao, A., Xu, C., Xu, Y., et al. (2023). A Survey on Vision Transformer. IEEE Trans. Pattern Anal. Mach. Intell. *45*, 87–110. https://doi.org/10.1109/TPAMI.2022.3152247.
43. Khan, S., Naseer, M., Hayat, M., Waqas Zamir, S., Shahbaz Khan, F., and Shah, M. (2021). Transformers in Vision: A Survey. Preprint at arXiv. https://doi.org/10.48550/arXiv.2101.01169.
44. Ganesh, P., Chen, Y., Lou, X., Khan, M.A., Yang, Y., Sajjad, H., Nakov, P., Chen, D., and Winslett, M. (2021). Compressing Large-Scale Transformer-Based Models: A Case Study on BERT. Trans. Assoc. Comput. Linguist. *9*, 1061–1080. https://doi.org/10.1162/tacl_a_00413.
45. Falcon-40B by TII. (2023). https://falconllm.tii.ae.
46. GPT-J Huggingface Model Description. (2023). https://huggingface.co/tiiuae/falcon-40b.
47. LLaMA Huggingface Model Description. (2023). https://huggingface.co/docs/transformers/main/model_doc/llama.

Patterns, Volume *4*

Supplemental information

Classification of integers based on residue classes via modern deep learning algorithms

Da Wu, Jingye Yang, Mian Umair Ahsan, and Kai Wang

SUPPLEMENTAL EXPERIMENTAL PROCEDURES

Here we document the prompts and detailed responses from LLMs to complement the contents in the Section "Results from Large Language Models (LLMs)" within main texts.

Responses from GPT-J-6B

When $p = 2$, we have the following two responses:

- **Prompt**: *There are various mathematical rules to check if an integer is divisible by 2, for instance*
  GPT-J: There are various mathematical rules to check if an integer is divisible by 2, for instance. For the numbers in your list, only the 1, 3, 9, and 17 are divisible by 2. For the others, divide by 2 to check that they work.\n\nA:\n \n This question was posted a while ago. This answer works for numbers in any base! Here's the full code that does what I posted before:\ndef divisibility(n

- **Prompt**: *How to check if an integer is divisible by 2, for instance we can*
  GPT-J: How to check if an integer is divisible by 2, for instance we can create some code that will check if a number is even or not and if it is even it will return true and if it is odd then it returns false and we can make code to check if a number in divisible by 3\n\nhow to check if an number is divisible by 2, for instance we can create some code that will check if a number is even or not and if it is even it will

When $p = 3$, we have the following two responses:

- **Prompt**: *There are various mathematical rules to check if an integer is divisible by* 3*, for instance*
  GPT-J: There are various mathematical rules to check if an integer is divisible by 3, for instance.\nAnother one to check if an integer is divisible by 5 or by 7.\n \nIn any case, these rules will be implemented differently in different programming languages.\nTo check if an integer is divisible by 3, you need to look and see if it's divisible by 3, if it is, it has to be divisible by 3. \nNot at all the same in

- **Prompt**: *How to check if an integer is divisible by* 3*, for instance we can*
  GPT-J: How to check if an integer is divisible by 3, for instance we can see that:\n\n- a number divisible by 3 has a remainder of 0 when divided by 3 is 0 or, in other words, 3 (i.e., $3\%3 = 0$)\n\n- a number divisible by 3 has a remainder of 1 when divided by 3 (i.e., $3/3 = 1$)\n\n- a number divisible by

When $p = 5$, we have the following two responses:

- **Prompt**: *There are various mathematical rules to check if an integer is divisible by* 5*, for instance*
  GPT-J: There are various mathematical rules to check if an integer is divisible by 5, for instance by multiplying every number with 5 until no integers remain that are divisible. Of course, in this specific example, you can get away with writing a loop. But for more complex rules, such as "Divisible by 5 until the number is divisible no more" or for the most general case where there might be several factors, it is more natural to use a recursion.\nIf we wanted to

- **Prompt**: *How to check if an integer is divisible by* 5*, for instance we can*
  GPT-J: How to check if an integer is divisible by 5, for instance we can do:\n»> x = 3\n»> a = 5\n»> b = len(x//a)\n»> b // 2\n2\n\nBut we can check if it is more easy using bitwise operators:\n»> x = 31\n»> a = 5\n»> x & a\n14\n\n»> x & 3\n0\n\n»> x & 5\n0\n\n»> x & 7

When $p = 7$, we have the following two responses:

- **Prompt**:*There are various mathematical rules to check if an integer is divisible by* 7*, for instance*
  GPT-J: There are various mathematical rules to check if an integer is divisible by 7, for instance this one:\nint divisibleby7 $= x\%7 == 0$;\n\nBut this one is new to me.\nint isDivisibleBy7(int x) \n if $(x! = 0\&\&(x\%7) == 0)$\n return true;\n else \n

- **Prompt**: *How to check if an integer is divisible by* 7*, for instance we can*
  GPT-J: How to check if an integer is divisible by 7, for instance we can use something like this :\nfunction ifDivisibleby7(num)\n if(num $\%7 == 0$)\n return true;\n else\n return 'The result is not divisible by 7, please check the value.';\n \n\n\nBut I plaza like to know if it is possible to use a single function to check this.

When $p = 11$, we have the following two responses:

- **Prompt**:*There are various mathematical rules to check if an integer is divisible by* 11*, for instance*
  GPT-J: There are various mathematical rules to check if an integer is divisible by 11, for instance:\nint x = 711;\nx % 11 == 0\n\nor \nx % 11 == 0 && x - x / 11!= 0\n\nbut I believe there is an easier way, using bitwise operators, which I do not understand.\n\nA:\n\nYou can use the bitwise AND (&) operator and the bitwise EQUALS to compare the two numbers.

- **Prompt**: *How to check if an integer is divisible by* 11*, for instance we can*
  GPT-J: How to check if an integer is divisible by 11, for instance we can check if 22 = 4 $*$ 11 because 2 is divisible by 11.\n\nA:\n\nThe only simple way I can think of is to do this using modular arithmetic, which is quite quick and probably not any slower than your own method.\nif x % 11 == 0:\n # x is evenly divisible by 11.\n\nA:\n\nI think the way to go

When $p = 13$, we have the following two responses:

- **Prompt**:*There are various mathematical rules to check if an integer is divisible by* 13*, for instance*
  GPT-J: There are various mathematical rules to check if an integer is divisible by 13, for instance:\n\nIf we take out the 2 from 3*4=12, we get this equation:\n\nIf we take out the zero from zero, we get:\n\nIt is not difficult to see that all the possibilities are divisible by 13.\n\nHowever, if we take out the zero from zero, we get:\n\nSince there are no numbers that are divisible by 13

- **Prompt**: *How to check if an integer is divisible by* 13*, for instance we can*
  GPT-J: How to check if an integer is divisible by 13, for instance we can check if a number is equal to 15 (for example, 2 is not equal to 15 because 14 > 14, so this is a correct answer):\n\nBut I do not know where to start and I need some help on this topic.\nI started to think about writing an algorithm for it, but I'm not sure if it's the correct way.\n\nA:\n\nI think you're correct

When $p = 17$, we have the following two responses:

- **Prompt**: *There are various mathematical rules to check if an integer is divisible by* 17*, for instance*
  GPT-J: There are various mathematical rules to check if an integer is divisible by 17, for instance the rule\nis x is divisible by 17 \nif and only if x= 2k, for some k, with k itself an integer.\nIs there a similar rule for the problem of checking if an integer is divisible by 23? I want to do it in JavaScript.\n\nA:\n\nThere are infinitely many numbers divisible by 17, but only 24 that aren't div.

- **Prompt**: *How to check if an integer is divisible by* 17*, for instance we can*
  GPT-J: How to check if an integer is divisible by 17, for instance we can do:\nvar number = 12;\n\nikiCheck(number,'17'); //returns true\n\nikiCheck(number,'1701'); //returns true\n\nikiCheck(number,'1800'); // returns false as 1800 is not divisible by 17\n\nikiCheck() prizmarija from the iki library, is a great tool to check if a number is divisible by

When $p = 19$, we have the following two responses:

- **Prompt**: *There are various mathematical rules to check if an integer is divisible by* 19*, for instance*
  GPT-J: There are various mathematical rules to check if an integer is divisible by 19, for instance:\n\nif (int value = 19;) System.out.println("Error: the value must be divisible by 19");\n\nBut what is the proper way to check a numberś divisibility by all the numbers from one to 9?\n\nA:\n\nYou could use something like that:\nprivate static Boolean isDividedByOneToNine(int number)

- **Prompt**: *How to check if an integer is divisible by* 19*, for instance we can*
  GPT-J: How to check if an integer is divisible by 19, for instance we can do this in the following way\n number = 1;\nif ((number % 19 == 0)) \n// do something\n\n\nBut this will always return false\nBut if we could check if the number is divisible by a defined integer (ex: 19), then we can do this \nnumber = 1;\nif (((number

When $p = 23$, we have the following two responses:

- **Prompt**: *There are various mathematical rules to check if an integer is divisible by* 23*, for instance*
  GPT-J: There are various mathematical rules to check if an integer is divisible by 23, for instance, and a test for division by 24.\n\nIn a program, this is done by the rules: if m is divisible by n then it divides by n.\n\nThe rule for division by 24 is called modulo division, and can be generalized to modulo r. By modulo r, an integer a is said to be a multiple of r, written $a = r * m$ as

- **Prompt**: *How to check if an integer is divisible by* 23*, for instance we can*
  GPT-J: How to check if an integer is divisible by 23, for instance we can check if a month is divisible by 4 or 5 or 6. \nI tried the following: \nif (((month.month % 4) == 0) || ((month.month % 5) == 0) || ((month.month % 6) == 0))\n System.out.println("Divisible by 4, 5 or 6");\n else \n

When $p = 29$, we have the following two responses:

- **Prompt**: *There are various mathematical rules to check if an integer is divisible by* 29*, for instance*
  GPT-J: There are various mathematical rules to check if an integer is divisible by 29, for instance, 29%3 = 0 or (I guess) 29%13 = 0, etc. But the algorithm could also be applied to a large number of factors.\n\nFor instance, if a number is factorised by a prime (say, 17), then the resulting prime factors could be applied to the original number, to check if that number is prime.\n\nFor example, 2^17*

- **Prompt**: *How to check if an integer is divisible by* 29*, for instance we can*
  GPT-J: How to check if an integer is divisible by 29, for instance we can divide it into 2 parts and ask if they match with 29?\nSo, I wonder how do I check if a given number is divisible by 29, such that (i.e.)\nif (n % 29 == 0) \n ...\n\n\nA:\n\nHow to check if an integer is divisible by 29, for instance we can divide it into 2 parts

When $p = 31$, we have the following two responses:

- **Prompt**: *There are various mathematical rules to check if an integer is divisible by* 31*, for instance*
  GPT-J: There are various mathematical rules to check if an integer is divisible by 31, for instance the following piece of code will display the output as 1,4,1,4,1,4,1,4,1,...\n divisible31 = 31;\nnumber = 1;\nwhile (number<=10^6)\n\n print number % divisible31.

- **Prompt**: *How to check if an integer is divisible by* 31*, for instance we can*
  GPT-J: How to check if an integer is divisible by 31, for instance we can check the value 4 and the result should be true.\n\n\n\nAs it has been said in another post, we are going to use a bitmask.\n\nA bitmask is a number where each bit is represented with a "0" or a "1". The sum of all the bits is always going to be the total number.\n\nFor example:\n\n4 is

Responses from Falcon-40B

When $p = 2$, we have the following two responses:

- **Prompt**: *There are various mathematical rules to check if an integer is divisible by 2, for instance*
  Falcon-40B: There are various mathematical rules to check if an integer is divisible by 2, for instance if the sum of the digits are even then it is divisible by 2 but the sum of the digits are odd doesn't mean it's not divisible by 2, in fact 1 is divisible by 2. So, how can you check whether an integer is divisible by 2? Here is another method of checking if an integer is divisible by 2, if you take the integer and replace all the digits with its equivalent binary number and check whether the binary number is divisible by 2, you will find that the original number is divisible by 2 or not.
  Example:
  - Is 23 divisible by 2? Yes/No
  - Is 45 divisible by 2? Yes/No
  - What is the binary representation $45 = 1011 = 1(2^0) + 1$

- **Prompt**: *How to check if an integer is divisible by 2, for instance we can*
  Falcon-40B: How to check if an integer is divisible by 2, for instance we can check by checking the last bit of the integer.
  Example: 10 is not divisible by 2, since the last bit is not a 2. But 12 is divisible by 2 since the last bit is 2. So we have to check the last bit and then check the last bit. We can do this by converting the integer to its binary representation (in a string) and then checking the last character, if it is equal to '1' then this number is divisible by 2. If this number does not have an even number of digits, then we need to add zeros to it. For this problem, we have added the number of leading zeros to each element. Then we can check that this binary string is divisible by 10 (by checking its parity). So, if it is divisible by 10, then we

When $p = 3$, we have the following two responses:

- **Prompt**: *There are various mathematical rules to check if an integer is divisible by* 3*, for instance*
  Falcon-40B: There are various mathematical rules to check if an integer is divisible by 3, for instance if the sum of its digits is divisible by 3.
  For 2, 3, 5, and 7 it is easy as these are prime numbers.
  For others :
  - check if the digits add up to a single digit number that is divisible by 3.
  - check if the numbers is divisible by 9 as 9 and multiples of 9 are also divisible by 3.
  This is the first time i am writing in this forum. Please correct me if i am wrong

- **Prompt**: *How to check if an integer is divisible by* 3*, for instance we can*
  Falcon-40B: How to check if an integer is divisible by 3, for instance we can use remainder operator in java. For example, if we want to check if integer num is divisible by 3, we can write following code: int num = 12; int mod; mod = num $\%3$; System.out.println(num+" is divisible by 3? "+(mod==0)); Above program would print following result: 12 is divisible by 3? true

When $p = 5$, we have the following two responses:

- **Prompt**: *There are various mathematical rules to check if an integer is divisible by* 5*, for instance*
  Falcon-40B: There are various mathematical rules to check if an integer is divisible by 5, for instance. If the unit's place of the integer is a multiple of 5 or ends in a 0 or 5, you know the integer is a multiple of five too. The rule I will be explaining in this article is how to check for the divisibility of an integer by 7. There is one simple rule for checking it and one other rule, which is more complicated but can help to make the checking easier. Divisibility by 7 The simple rule is that an integer is divisible by 7 if the sum of its digits is a multiple of 7. Examples - The number 1, which is the smallest integer which is divisible by 7, has a unit place (1) that ends with a 0. (0*1) + (1*1) =2,

- **Prompt**: *How to check if an integer is divisible by* 5*, for instance we can*
  Falcon-40B: How to check if an integer is divisible by 5, for instance we can use this formula to check if an integer N is divisible by 5.
  N is divisible by 5 if and only if (N & 1) == 1
  For instance,
  N% 2 == 0 if (N & 1) == 0 <=> N%5 == 0 if (N & 1) == 1
  The code to check if an integer is divisible by 5 is
  int isdivisibleby5(int N) return (N & 1) == 1;
  If you find this post on "Check if divisible by 5".

When $p = 7$, we have the following two responses:

- **Prompt**: *There are various mathematical rules to check if an integer is divisible by* 7*, for instance*
  Falcon-40B: There are various mathematical rules to check if an integer is divisible by 7, for instance:
  - 7 x 2 = 14, the sum of the digits is divisible by 7, (14 / 7 = 2)
  - 7 x 6 = 42, the sum of the digits is divisible by 7 (42 / 7 = 6)
  - 7 x 7 = 49, the sum of the digits is divisible by 7 (49 / 7 = 7)
  So you can check divisibility by 7 by multiplying a candidate number by 7 and check if the result is divisible by 7. This is called the 'trial division test'.
  For larger integers it is quicker to check if the remainder of the number divided by 7 is a multiple of 7. In the case it is a multiple of

- **Prompt**: *How to check if an integer is divisible by* 7*, for instance we can*
  Falcon-40B: How to check if an integer is divisible by 7, for instance we can use modulo operator in Java. We will get remainder by dividing one number by another. If the remainder is 0 then it is divisible by 7. We are using the $\%$ operator here. Java Program to check if number is divisible by 7 import java.util.Scanner; public class Main public static void main(String[] args) int num, remainder; Scanner scanner = new Scanner(System.in); System.out.println("Enter any number."); num = scanner.nextInt(); System.out.printf("7 is a factor of $\%$d.\n", num); remainder = num $\%$ 7; if(remainder == 0) System.out.println(num + " is a multiple of 7."); else System.out.println(num + " is

When $p = 11$, we have the following two responses:

- **Prompt**: *There are various mathematical rules to check if an integer is divisible by* 11*, for instance*
  Falcon-40B: There are various mathematical rules to check if an integer is divisible by 11, for instance, if the sum of its digits is divisible by 11. This problem is different. The input is given as a (potentially huge) integer n. Your function needs to check the divisibility by 11 of that integer. If the integer is not divisible by 11, the function should return 0, otherwise it needs to return 1. The function should not throw exceptions of any kind. Examples:
  input: 10 output: 1 input: -10 output: 0 input: 1111 output: 1
  Note that the input value could be of any size. If the input value is too big to store in an Int or Long, your function can return false, or throw an exception, or do anything else that indicates failure. However, you do need to test for the divisibility of the values 11 and

- **Prompt**: *How to check if an integer is divisible by* 11*, for instance we can*
  Falcon-40B: How to check if an integer is divisible by 11, for instance we can use this trick to check if any given year is a leap year or not.

The idea is based on the fact that the sum of the digits of a number is a multiple of 11 if and only if the number itself is a multiple of 11.
For Example:
Year 1976 has sum of digits 2 7 1 9 = 21
21 = 2*11, So it's a Leap year
How it works?
- Take the given number (integer).
- Sum all its digits.
- If the sum is a multiple of 11, Then it is a leap year, otherwise not.
Example:
#include <iostream> #include <cmath> using namespace std; bool isLeap(int n) int s = 0; while (n

When $p = 13$, we have the following two responses:

- **Prompt**: *There are various mathematical rules to check if an integer is divisible by* 13*, for instance*
  Falcon-40B: There are various mathematical rules to check if an integer is divisible by 13, for instance, if the last digit of an integer is divisible by 2 then the whole number would be divisible by 13. Here is the logic for that:
  - If a number is divisible by 13, the digit sum of the number should be divisible by 2 and not by 3 or 9
  - If a number is not divisible by 13, the sum of the digits of the number should also be divisible by 2, 3 and 9.

- **Prompt**: *How to check if an integer is divisible by* 13*, for instance we can*
  Falcon-40B: How to check if an integer is divisible by 13, for instance we can use a simple trick. If the integer number is divisible by 5 and divisible by 2 then it is divisible by 13. So lets see some simple C++ code. #include <iostream> using namespace std; void checkDivBy13(int n) $/*$ If a number is a multiple of 2 or 5, it is also a multiple of 10. */ int div10 = (n % 10) == 0; int div5 = (n % 5) == 0; if (div10 || div5) cout « n « " is divisible by 13" « endl; void main() checkDivBy13(1); checkDivBy13(2); checkDivBy13(3); checkDiv

When $p = 17$, we have the following two responses:

- **Prompt**: *There are various mathematical rules to check if an integer is divisible by* 17*, for instance*
  Falcon-40B: There are various mathematical rules to check if an integer is divisible by 17, for instance by using the remainder after division by 17, or using the divisibility by 17 rule for odd and even integers. Here is the detail for how to check if a number is divisible by 17.
  1. Integer is Divisible by 17 Rule for Even Integers To find out if an even integer is divisible by 17, the last digit must be 7.
  For even numbers: 2x + 5
  - If the last digit is 7, then the number is divisible by 17.
  - If the last digit is not 7, then the number is not divisible by 17.
  2. Integer is Divisible by 17 Rule for Odd Integers To find out if an odd integer is divisible by 17, the last 3 digits must be divisible by 17.

- **Prompt**: *How to check if an integer is divisible by* 17*, for instance we can*
  Falcon-40B: How to check if an integer is divisible by 17, for instance we can check if 17 is a prime number, is the number divisble by 2, or any number divisble by 3,4,... This program checks if a given integer n is divisible by both 17 and 9. This code is contributed by the user jatin. C Output:
  Enter an integer to check whether its prime: 17 Prime Number.
  Enter an integer to check whether its prime: 18 Non Prime Number.
  Please write comments if you find anything incorrect, or you want to share more information about the topic discussed above.

When $p = 19$, we have the following two responses:

- **Prompt**: *There are various mathematical rules to check if an integer is divisible by* 19*, for instance*
  Falcon-40B: There are various mathematical rules to check if an integer is divisible by 19, for instance:
  - 19 / 1 = 19 is divisible by 19 because 19 × 1/1 = 19
  - 19 / 2 = 9 and 9/2 = 4 remainder 1
  - 19 / 19 = 1 and 1/19 = 0 remainder 1
  - 19 / 38 = 0 and 38/19 = 2 remainder 0
  - 19 / 57 = 0 and 57/19 = 3 remainder 0
  - 19 / 76 = 0 and 76/19 = 4 remainder 0
  - 19 / 95 = 0 and 95/19 = 5 remainder 0

- **Prompt**: *How to check if an integer is divisible by* 19*, for instance we can*
  Falcon-40B: How to check if an integer is divisible by 19, for instance we can use % (modulus operator) and divide by 19, then check if the remainder is 0 (zero), if yes then the number is divisible by 19. Example: The following is how to check if the number 19 is divisible by 19 using modulus and logical AND operator. int isDivisibleBy19(int n)
  return n % 19 == 0;

  The following is how to check for the divisibility of 19 using the division sign (/) and logical AND (&&). int isDivisibleBy19(int n)
  return n % 19 == 0 && n!= 0;

  You can find more information on this topic at:

When $p = 23$, we have the following two responses:

- **Prompt**: *There are various mathematical rules to check if an integer is divisible by* 23*, for instance*
  Falcon-40B: There are various mathematical rules to check if an integer is divisible by 23, for instance: - (23*23) % 23 == 0
  - (23*23*23) % 23 == 0
  - (23*23*23*23) % 23 == 0
  -... But I found a simple rule to check if the number is divisible by 23, and I wonder if it could be proven : 1 + 2 + 3 +... + N = N(N+1)/2 is divisible by 23. I tried to look at the proof in the other thread (the one linked at the beginning) but I didn't understand it.

- **Prompt**: *How to check if an integer is divisible by* 23*, for instance we can*
  Falcon-40B: How to check if an integer is divisible by 23, for instance we can check 12345 is divisible by 23 then we have to check the unit digits of 456, the unit digits of 456 are 6, then we have to check if the unit digits of 6 are divisible by 23, so 6 divided by 23 is 0. 456 is divisible by 23. We can find whether an integer is divisible by 23 or not by using the Modulo (The program below will check if an integer is divisible by 23 or not. #include <stdio.h> int main() int number, remainder, divisor = 23; printf("Enter an integer: "); scanf("%d", &number); remainder = number % divisor; if(remainder == 0) printf("The given integer is

When $p = 29$, we have the following two responses:

- **Prompt**: *There are various mathematical rules to check if an integer is divisible by* 29*, for instance*
  Falcon-40B: There are various mathematical rules to check if an integer is divisible by 29, for instance: - If you add the digits of the number, the sum must be divisible by 3. If not, add 1 to this sum. - If you divide this number by 8, it must have a remainder of 1, 2, 3, 4, 5 or 6. - The sum of the numbers in all rows of a Pythagorean triplet with 29 as a base, must be divisible by 29. If the integer is not divisible by 29, then it must be divisible by one or multiple of 5, because 5 is also a factor. So, you must first check if the integer is divisible by 5. If the integer is not divisible by both 3 and 5, it is certainly not divisible by 29. So, you

- **Prompt**: *How to check if an integer is divisible by* 29*, for instance we can*
  Falcon-40B: How to check if an integer is divisible by 29, for instance we can check if 1 is divisible by 29 or not.
  If an integer is divisible by 29, it would have either 0 or 29 as a remainder. So in our case, we will do: 1 % 29 = 0 or
  1 % 29 = 29
  This method is called modulo (or modulus) and is very useful and used often in computer science. It is also known as remainder.
  If the remainder is 0, then the integer is divisible by 29. If it is a positive non-zero then the integer is not divisible by 29.
  In this case,
  - 1 % 29 = 0
  - 2 % 29 = 2
  - 3 % 29 = 3

When $p = 31$, we have the following two responses:

- **Prompt**: *There are various mathematical rules to check if an integer is divisible by* 31*, for instance*
  Falcon-40B: There are various mathematical rules to check if an integer is divisible by 31, for instance, the integer is divisible by 31 if the last digit is divisible by 3 or if the last two digits are divisible by 6. Here we will discuss some

of the simple methods to check if an integer is evenly divisible by 31 or not. Check Divisibility by 3: An integer can be evenly divisible by 31 only if its last digit is divisible by 3 or if all of the digits are multiples of 3, for example, 12345 and 123456789. Therefore, if the last digit of any number is not a multiple of 3 or all the digits are not multiple of 3, we can say that the number is not evenly divisible by 31. Therefore, if the last digit of the number is a multiple of 3 or all the digits are divisible by 3, then the

- **Prompt**: *How to check if an integer is divisible by* 31*, for instance we can*
  Falcon-40B: How to check if an integer is divisible by 31, for instance we can find out if a number is divisible by 31 by dividing 31 with that number. But if the number is a large one, it is time consuming to find out the result. In this article we are going to show two easy methods to help you check if a number is divisible by 31. One is manual way, another is by using an online tool. The manual method is to check each digit of number from right to left. If a digit is greater than or equal to 5, we have to add 1 to the quotient. If the quotient is not divisible by 31, the number is not divisible by 31.
  Here is an example: The number is 1023. First check if the last digit 3 is >=5, it is greater, we add 1 to the quotient and get 6/10

Responses from LLaMA-7B

When $p = 2$, we have the following two responses:

- **Prompt**: *There are various mathematical rules to check if an integer is divisible by 2, for instance*
  LLaMA: There are various mathematical rules to check if an integer is divisible by 2, for instance 2, 4, 6, 8, 10, 12, 14, 16, 18, 20, 22, 24, 26, 28, 30, 32, 34, 36, 38, 40, 42, 44, 46, 48, 50, 52, 54, 56, 58, 60, 62, 64, 66, 68, 70, 72, 74, 76, 78, 80, 82,

- **Prompt**: *How to check if an integer is divisible by 2, for instance we can*
  LLaMA: How to check if an integer is divisible by 2, for instance we can check if 10 is divisible by 2 by dividing 10 by 2 and checking if the result is an integer.\nIf the result is an integer then the number is divisible by 2, if the result is not an integer then the number is not divisible by 2.\nIn the above example, 10 is divisible by 2 because 10/2 = 5 and 5 is an integer.\nIn the above example, 11 is not divisible by 2 because 11/2 = 5.5 and 5.5 is not an integer.\nIn the above example, 10 is divisible by 2 because $10\%2 = 0$ and 0 is

When $p = 3$, we have the following two responses:

- **Prompt**: *There are various mathematical rules to check if an integer is divisible by* 3*, for instance*
  LLaMA: There are various mathematical rules to check if an integer is divisible by 3, for instance 3n+1, 3n+2, 3n+0, 3n-1, 3n-2, 3n-0.\nBut I have found a simple rule to check if an integer is divisible by 3.\nIf the sum of the digits of an integer is divisible by 3, then the integer is divisible by 3.\nFor example, 123456789 is divisible by 3 because 1+2+3+4+5+6+7+8+9=45 and 45 is divisible by 3.\nIf the sum of the digits of an integer is not divisible by 3, then the integer is not divisible

- **Prompt**: *How to check if an integer is divisible by* 3*, for instance we can*
  LLaMA: How to check if an integer is divisible by 3, for instance we can check if 12 is divisible by 3 by dividing 12 by 3 and checking if the remainder is 0.\nWe can also check if an integer is divisible by 3 by checking if the sum of the digits of the integer is divisible by 3.\nFor instance, 12 is divisible by 3 because 1 + 2 = 3 and 3 is divisible by 3.\n123 is divisible by 3 because 1 + 2 + 3 = 6 and 6 is divisible by 3.\n1234 is divisible by 3 because 1 + 2 + 3 + 4 = 10 and 10 is divisible by

When $p = 5$, we have the following two responses:

- **Prompt**: *There are various mathematical rules to check if an integer is divisible by* 5*, for instance*
  LLaMA: There are various mathematical rules to check if an integer is divisible by 5, for instance, if the number is divisible by 5, then the last digit must be 0, 5, or 0.\nThe number 12345 is divisible by 5 because the last digit is 5.\nThe number 123456 is not divisible by 5 because the last digit is 6.\nThe number 1234567 is not divisible by 5 because the last digit is 7.\nThe number 12345678 is not divisible by 5 because the last digit is 8.\nThe number 12345678

- **Prompt**: *How to check if an integer is divisible by* 5*, for instance we can*
  LLaMA: How to check if an integer is divisible by 5, for instance we can check if 12 is divisible by 5 by doing 12 % 5 = 2.\nHow to check if an integer is divisible by 10, for instance we can check if 12 is divisible by 10 by doing 12 % 10 = 2.\nHow to check if an integer is divisible by 15, for instance we can check if 12 is divisible by 15 by doing 12 % 15 = 2.\nHow to check if an integer is divisible by 20, for instance we can check if 12 is divisible by 20

When $p = 7$, we have the following two responses:

- **Prompt**: *There are various mathematical rules to check if an integer is divisible by* 7*, for instance*
  LLaMA: There are various mathematical rules to check if an integer is divisible by 7, for instance 7 divides n if and only if the sum of the digits of n is divisible by 7.\nThe following program uses this rule to check if a number is divisible by 7.\n// Check if a number is divisible by 7.\n// Sum of the digits of the number.\n// Check if the sum is divisible by 7.\n// Print the result of the test.\n// The number is divisible by 7.\n// The number is not divisible by 7.\nThe following program uses the same rule to check if a number is divisible by 7.\n// Check if a number is divisible by 7 using the sum of the digits.\n// The number is not divisible by 7.

- **Prompt**: *How to check if an integer is divisible by* 7*, for instance we can*
  LLaMA: How to check if an integer is divisible by 7, for instance we can check if 123456789 is divisible by 7.\nThe first step is to check if the last digit of the number is divisible by 7.\nIf the last digit is divisible by 7, then we can check if the number formed by the first n-1 digits is divisible by 7.\nIf the number formed by the first n-1 digits is divisible by 7, then the number is divisible by 7.\nIf the number formed by the first n-1 digits is not divisible by 7, then the number is not divisible by 7.\nIf the last digit is not divisible by 7, then the number is not divisible by 7.\nThe.

When $p = 11$, we have the following two responses:

- **Prompt**: *There are various mathematical rules to check if an integer is divisible by* 11*, for instance*
  LLaMA: There are various mathematical rules to check if an integer is divisible by 11, for instance, if the last digit is 1, 3, 7 or 9, then the number is divisible by 11.\nIf the last digit is 2, 4, 6 or 8, then the number is not divisible by 11.\nIf the last digit is 0, 5 or 6, then the number is divisible by 11.\nIf the last digit is 1, 3, 7 or 9, then the number is divisible by 11.\nIf the last digit is 2, 4, 6 or 8, then the number is not divisible

- **Prompt**: *How to check if an integer is divisible by* 11*, for instance we can*
  LLaMA: How to check if an integer is divisible by 11, for instance we can check if 11 is divisible by 11.\nThe following code will check if an integer is divisible by 11.\nThe following code will check if an integer is divisible by 11. The code will return true if the integer is divisible by 11.\nThe following code will check if an integer is divisible by 11. The code will return false if the integer is not divisible by 11.\nThe following code will check if an integer is divisible by 11. The code will return true if the integer is divisible by 11. The code will return false if the integer is not divis

When $p = 13$, we have the following two responses:

- **Prompt**: *There are various mathematical rules to check if an integer is divisible by* 13*, for instance*
  LLaMA: There are various mathematical rules to check if an integer is divisible by 13, for instance, if the number is divisible by 13, then the sum of the digits of the number must be divisible by 13.\nThe sum of the digits of a number is the sum of the digits of the number.\nThe sum of the digits of a number is the sum of the digits of the number. The sum of the digits of a number is the sum of the digits of the number.\nThe sum of the digits of a number is the sum of the digits of the number. The sum of the digits of a number is the sum of the digits of the number. The sum of the digits of a number is the sum of the digits of the

- **Prompt**: *How to check if an integer is divisible by* 13*, for instance we can*
  LLaMA: How to check if an integer is divisible by 13, for instance we can check if 123456789 is divisible by 13.\nThe first step is to check if the number is divisible by 10.\nIf the number is divisible by 10, then it is divisible by 13.\nIf the number is not divisible by 10, then it is not divisible by 13.\nThe number 123456789 is divisible by 10, so it is divisible by 13.\nThe number 123456789 is not divisible by 10, so it is not

When $p = 17$, we have the following two responses:

- **Prompt**: *There are various mathematical rules to check if an integer is divisible by* 17*, for instance*
  LLaMA: There are various mathematical rules to check if an integer is divisible by 17, for instance 17 is a prime number, so it is divisible by 1 and 17.\n17 is divisible by 17, because 17 = 17 x 1.\n17 is divisible by 17, because 17 = 17 x 1. 17 is divisible by 17, because 17 = 17 x 1. 17 is divisible by 17, because 17 = 17 x 1. 17 is divisible by 17, because 17 = 17 x 1. 17 is divisible by 17, because 17 = 17 x 1. 1

- **Prompt**: *How to check if an integer is divisible by* 17*, for instance we can*
  LLaMA: How to check if an integer is divisible by 17, for instance we can check if 17 divides 1000.\nWe can check if 17 divides 1000 by using the modulo operator.\nThe modulo operator is denoted by %.\nThe modulo operator returns the remainder of the division of two numbers.\nFor example, 1000 % 17 = 15.\nSo, 17 divides 1000.\n1000 % 17 = 15\n15 % 17 = 15\n15 % 17 = 15 and so on.\nSo, 17 divides 15.\n15 % 17 = 15 and so on.\nSo,

When $p = 19$, we have the following two responses:

- **Prompt**: *There are various mathematical rules to check if an integer is divisible by* 19*, for instance*
  LLaMA: There are various mathematical rules to check if an integer is divisible by 19, for instance, if the integer is divisible by 19, then the sum of the digits of the integer must be divisible by 19.\nThe sum of the digits of an integer is the sum of the digits of the integer.\nThe sum of the digits of an integer is the sum of the digits of the integer. The sum of the digits of an integer is the sum of the digits of the integer.\nThe sum of the digits of an integer is the sum of the digits of the integer. The sum of the digits of an integer is the sum of the digits of the integer. The sum of the digits of an integer is the sum of the digits of the

- **Prompt**: *How to check if an integer is divisible by* 19*, for instance we can*
  LLaMA: How to check if an integer is divisible by 19, for instance we can check if 19 is divisible by 19.\nThe following code will check if an integer is divisible by 19.\nThe following code will check if an integer is divisible by 19. The code will return true if the integer is divisible by 19.\nThe following code will check if an integer is divisible by 19. The code will return false if the integer is not divisible by 19.\nThe following code will check if an integer is divisible by 19. The code will return true if the integer is divisible by 19. The code will return false if the integer is not divis

When $p = 23$, we have the following two responses:

- **Prompt**: *There are various mathematical rules to check if an integer is divisible by* 23*, for instance*
  LLaMA: There are various mathematical rules to check if an integer is divisible by 23, for instance, if the number is divisible by 23, then the sum of the digits of the number must be divisible by 23.\nThe sum of the digits of a number is the sum of the digits of the number.\nThe sum of the digits of a number is the sum of the digits of the number. The sum of the digits of a number is the sum of the digits of the number.\nThe sum of the digits of a number is the sum of the digits of the number. The sum of the digits of a number is the sum of the digits of the number. The sum of the digits of a number is the sum of the digits of the

- **Prompt**: *How to check if an integer is divisible by* 23*, for instance we can*
  LLaMA: How to check if an integer is divisible by 23, for instance we can check if 123456789 is divisible by 23.\nThe first step is to check if the number is divisible by 3.\nIf the number is divisible by 3, then it is divisible by 23.\nIf the number is not divisible by 3, then it is not divisible by 23.\nIf the number is divisible by 3, then it is divisible by 23. If the number is not divisible by 3, then it is not divisible by 23.\nThe number is divisible by 3.\nThe number is not divisible

When $p = 29$, we have the following two responses:

- **Prompt**: *There are various mathematical rules to check if an integer is divisible by* 29*, for instance*
  LLaMA: There are various mathematical rules to check if an integer is divisible by 29, for instance, if the number is even, then it is divisible by 29. If the number is odd, then it is not divisible by 29.\nIf the number is divisible by 29, then the last digit of the number is 0, 2, 4, 6, 8 or 9.\nIf the number is not divisible by 29, then the last digit of the number is 1, 3, 5, 7 or 0.\nIf the number is divisible by 29, then the sum of the digits of the number is divisible by 29.\nIf

- **Prompt**: *How to check if an integer is divisible by* 29*, for instance we can*
  LLaMA: How to check if an integer is divisible by 29, for instance we can check if 12 is divisible by 29.\nThe first step is to check if the number is divisible by 2.\nIf the number is divisible by 2, then we can check if it is divisible by 29.\nIf the number is not divisible by 2, then we can check if it is divisible by 29.\nIf the number is divisible by 2, then we can check if it is divisible by 29. If the number is not divisible by 2, then we can check if it is divisible by 29.\nIf the number is divisible by 2

When $p = 31$, we have the following two responses:

- **Prompt**: *There are various mathematical rules to check if an integer is divisible by* 31*, for instance*
  LLaMA: There are various mathematical rules to check if an integer is divisible by 31, for instance, if the number is divisible by 31, then the sum of the digits of the number must be divisible by 31.\nThe number 31 is a prime number.\nThe number 31 is a composite number.\nThe number 31 is a Fibonacci number.\n The number 31 is a Harshad number.\nThe number 31 is a Sophie Germain prime.\n The number 31 is a Chen prime.\nThe number 31 is a safe prime.\nThe number 31 is a happy number.\nThe number 31 is a centered square number.\n

- **Prompt**: *How to check if an integer is divisible by* 31*, for instance we can*
  LLaMA: How to check if an integer is divisible by 31, for instance we can check if 123456789 is divisible by 31.\nThe first step is to check if the number is divisible by 3.\nIf the number is divisible by 3, then it is divisible by 31.\nIf the number is not divisible by 3, then it is not divisible by 31.\nIf the number is divisible by 3, then it is divisible by 31. If the number is not divisible by 3, then it is not divisible by 31.\nIf the number is divisible by 3, then it is divisible by

Responses from GPT-4 with code interpreter

- **Prompt:** Can you design a deep learning algorithm to determine if a number can be divided by 17?
  GPT-4: Certainly! But before we delve into a deep learning solution, it's important to note that traditional algorithms can efficiently determine whether a number is divisible by 17. The division operation or using the divisibility rule for 17 can solve this problem in a straightforward manner.

  However, if you're interested in a deep learning approach for educational purposes or to see how a neural network might handle this task, we can attempt that. Here's a basic outline of a deep learning model to solve this problem:

  1. **Dataset Generation**: Generate a dataset containing numbers and their labels: 1 if divisible by 17, 0 otherwise.
     - For instance:

       17 -> 1,
       34 -> 1,
       16 -> 0,
       35 -> 0,
       ...

  2. **Data Representation**: Represent numbers in a binary format so they can be input to the neural network.

  3. **Neural Network Model**: You can use a simple feed-forward neural network (like MLP) for this task. A deep architecture might be overkill, but the purpose here is experimentation.

  4. **Training**: Use the dataset from step 1 to train the neural network. The model will learn to recognize patterns in the binary representation of numbers to determine divisibility.

  Here's a simple model using TensorFlow/Keras:

```
import numpy as np
from tensorflow import keras
from keras.models import Sequential
from keras.layers import Dense, Input
from keras.utils import to_categorical

# Data generation
samples = 100000
X = np.array(range(samples))
y = np.where(X % 17 == 0, 1, 0)

# Convert numbers to binary format
max_length = int(np.ceil(np.log2(samples)))
X_binary =
np.array([list(format(x, '0{}b'.format(max_length))) for x in X],dtype=np.uint8)

# Split data into train and test sets
split = int(0.8 * samples)
X_train, X_test = X_binary[:split], X_binary[split:]
y_train, y_test = y[:split], y[split:]

# Neural network model
model = Sequential([
    Input(shape=(max_length,)),
    Dense(128,activation='relu'),
    Dense(64,activation='relu'),
    Dense(32,activation='relu'),
    Dense(1,activation='sigmoid')
])

model.compile(optimizer='adam',loss='binary_crossentropy',metrics=['accuracy'])
model.fit(X_train,y_train,epochs=10,batch_size=32,validation_data=(X_test,y_test))
```

```
# Evaluation
loss, accuracy = model.evaluate(X_test, y_test)
print(f"Accuracy:{accuracy*100:.2f}%")
```

Remember, this is a fun experiment to see if deep learning can learn the pattern, but in practice, using traditional algorithms is much more efficient for this particular problem.

SUPPLEMENTAL ITEMS

Prime N	Solution by ChatGPT on divisibility rule by N
2	To check if an integer is divisible by 2, you can use a very simple rule: check the last digit of the integer. If the last digit is an even number (0, 2, 4, 6, or 8), the integer is divisible by 2. If the last digit is an odd number, the integer is not divisible by 2. For example:<br>1. The integer 246 is divisible by 2 because the last digit is 6 (an even number).<br>2. The integer 357 is not divisible by 2 because the last digit is 7 (an odd number). **[Correct]**
3	To check if an integer is divisible by 3, you can use a simple rule based on the sum of its digits. If the sum of the digits of the integer is divisible by 3, then the integer itself is divisible by 3.<br>Here's the method:<br>1. Add up all the digits of the integer.<br>2. If the sum of the digits is divisible by 3 (including if it's 0), then the original integer is also divisible by 3.<br>Let's illustrate this method with an example: Let's check if the integer 351 is divisible by 3: Sum of digits: 3 + 5 + 1 = 9. Since 9 is divisible by 3 (3 * 3 = 9), the original integer (351) is also divisible by 3. **[Correct]**
5	To check if an integer is divisible by 5, you can use a simple rule: check the last digit of the integer. If the last digit is either 0 or 5, the integer is divisible by 5. If the last digit is not 0 or 5, the integer is not divisible by 5. For example: The integer 240 is divisible by 5 because the last digit is 0. **[Correct]**
7	To check if an integer is divisible by 7, you can use the following method:<br>1. Take the last digit of the integer.<br>2. Multiply this last digit by 2.<br>3. Subtract the result from step 2 from the remaining part of the integer (excluding the last digit).<br>4. If the resulting number is divisible by 7 (including if it's 0), then the original integer is also divisible by 7.<br>Let's illustrate this method with an example: Let's check if the integer 154 is divisible by 7:<br>1. Last digit: 4<br>2. Multiply last digit by 2: 4 * 2 = 8<br>3. Remaining part of the integer after removing the last digit: 15<br>4. Subtract the result from step 2 from the remaining part: 15 - 8 = 7<br>Since 7 is divisible by 7 (7 * 1 = 7), the original integer (154) is also divisible by 7. **[Correct]**

Table S1. Response given by ChatGPT on the question "*How to check if an integer can be divided by* N" for $N = 2, 3, 5$ and 7. The bold face texts "**[Correct]**" or "**[Wrong]**" indicate whether the response from ChatGPT is a mathematically correct statement or not. This is related to section "Results from Large Language Models (LLMs)" in the main texts. The rest Table S2,S3,S4,S5,S6 follow the exact same spirit.

Prime N	Solution by ChatGPT on divisibility by N
11	To check if an integer is divisible by 11, you can use the following method:<br>1. Starting from the rightmost digit of the integer, alternately add and subtract the digits.<br>2. If the result is divisible by 11 (including if it's 0), then the original integer is also divisible by 11.<br>Let's illustrate this method with an example:<br>Let's check if the integer 363 is divisible by 11:<br>1. Starting from the rightmost digit: 3<br>2. Move to the next digit and subtract it: 3 - 6 = -3<br>3. Move to the next digit and add it: -3 + 3 = 0<br>Since the result is 0, which is divisible by 11 (11 * 0 = 0), the original integer (363) is also divisible by 11.<br>Let's try another example with the integer 154:<br>1. Starting from the rightmost digit: 4<br>2. Move to the next digit and subtract it: 4 - 5 = -1<br>Since the result is not divisible by 11, the original integer (154) is not divisible by 11.<br>This method is a simple and efficient way to check if an integer is divisible by 11 without performing the full division. **[Correct]**
13	To check if an integer is divisible by 13, you can use the following method:<br>1. Take the last digit of the integer.<br>2. Multiply this last digit by 4.<br>3. Add the result from step 2 to the remaining part of the integer after removing the last digit.<br>4. If the resulting number is divisible by 13 (including if it's 0), then the original integer is also divisible by 13.<br>Let's illustrate this method with an example:<br>Let's check if the integer 156 is divisible by 13:<br>1. Last digit: 6<br>2. Multiply last digit by 4: 6 * 4 = 24<br>3. Remaining part of the integer after removing the last digit: 15<br>4. Add the result from step 2 to the remaining part: 15 + 24 = 39<br>Since 39 is divisible by 13 (13 * 3 = 39), the original integer (156) is also divisible by 13.<br>Let's try another example with the integer 289:<br>1. Last digit: 9<br>2. Multiply last digit by 4: 9 * 4 = 36<br>3. Remaining part of the integer after removing the last digit: 28<br>4. Add the result from step 2 to the remaining part: 28 + 36 = 64<br>Since 64 is not divisible by 13, the original integer (289) is not divisible by 13.<br>This method is a simple and efficient way to check if an integer is divisible by 13 without performing the full division. **[Correct]**

Table S2. Response given by ChatGPT on the question "*How to check if an integer can be divided by* N" for $N = 11$ and 13.

Prime N	Solution by ChatGPT on divisibility by N
17	To check if an integer is divisible by 17, you can use the following method:<br>1. Take the last digit of the integer and multiply it by 5.<br>2. Subtract the result from step 1 from the remaining part of the integer (excluding the last digit).<br>3. If the resulting number is divisible by 17 (including if it's 0), then the original integer is also divisible by 17.<br>Let's illustrate this method with an example: Let's check if the integer 272 is divisible by 17:<br>1. Last digit: 2<br>2. Multiply last digit by 5: 2 * 5 = 10<br>3. Remaining part of the integer after removing the last digit: 27<br>4. Subtract the result from step 2 from the remaining part: 27 - 10 = 17<br>Since 17 is divisible by 17 (17 * 1 = 17), the original integer (272) is also divisible by 17. Let's try another example with the integer 357:<br>1. Last digit: 7<br>2. Multiply last digit by 5: 7 * 5 = 35<br>3. Remaining part of the integer after removing the last digit: 35<br>4. Subtract the result from step 2 from the remaining part: 35 - 35 = 0<br>Since 0 is divisible by 17, the original integer (357) is also divisible by 17. **[Correct]**
19	To check if an integer is divisible by 19, you can use the following method:<br>1. Take the last digit of the integer.<br>2. Multiply this last digit by 2.<br>3. Subtract the result from step 2 from the remaining part of the integer (excluding the last digit).<br>4. If the resulting number is divisible by 19 (including if it's 0), then the original integer is also divisible by 19.<br>Let's illustrate this method with an example: Let's check if the integer 1148 is divisible by 19:<br>1. Last digit: 8<br>2. Multiply last digit by 2: 8 * 2 = 16<br>3. Remaining part of the integer after removing the last digit: 114<br>4. Subtract the result from step 2 from the remaining part: 114 - 16 = 98<br>Since 98 is divisible by 19 (19 * 5 = 95), the original integer (1148) is also divisible by 19. Let's try another example with the integer 256:<br>1. Last digit: 6<br>2. Multiply last digit by 2: 6 * 2 = 12<br>3. Remaining part of the integer after removing the last digit: 25<br>4. Subtract the result from step 2 from the remaining part: 25 - 12 = 13<br>Since 13 is not divisible by 19, the original integer (256) is not divisible by 19. **[Wrong]**

Table S3. Response given by ChatGPT on the question "*How to check if an integer can be divided by N*" for $N = 17$ and 19. For $N = 19$, the correct answer is that one need to *add* (not minus) the two times the last digit to the remaining part of the integer.

<table>
<tr><th>Prime N</th><th>Solution by ChatGPT on divisibility by N</th></tr>
<tr><td>23</td><td>

To check if an integer can be divided by 23, you can use the modulo operation (%), which gives you the remainder when one number is divided by another. If the remainder is 0, then the integer is divisible by 23. Here's how you can do it in various programming languages:

1. Python:

```
num = int(input("Enter an integer: "))
if num % 23 == 0:
    print(f"{num} is divisible by 23.")
else:
    print(f"{num} is not divisible by 23.")
```

2. JavaScript:

```
const num = parseInt(prompt("Enter an integer: "));

if (num % 23 === 0) {
    console.log(‘${num} is divisible by 23.‘);
} else {
    console.log(‘${num} is not divisible by 23.‘);
}
```

3. C++:

```
#include <iostream>
using namespace std;

int main() {
    int num;
    cout << "Enter an integer: ";
    cin >> num;

    if (num % 23 == 0) {
        cout << num << " is divisible by 23." << endl;
    } else {
        cout << num << " is not divisible by 23." << endl;
    }

    return 0;
}
```

As with the previous example, replace the input mechanism with the one suitable for your program. The key point is using the modulo operator (%) to check if the remainder is zero when divided by 29. **[Correct]**

</td></tr>
</table>

Table S4. Response given by ChatGPT on the question "*How to check if an integer can be divided by* 23".

Prime N	Solution by ChatGPT on divisibility by N
29	To check if an integer can be divided by 29, you can follow a similar approach as before and use the modulo operation (%). If the remainder is 0, then the integer is divisible by 29. Here's how you can do it in various programming languages:<br><br>1. Python:<br>`num = int(input("Enter an integer: "))`<br>`if num % 29 == 0:`<br>`    print(f"{num} is divisible by 29.")`<br>`else:`<br>`    print(f"{num} is not divisible by 29.")`<br><br>2. JavaScript:<br>`const num = parseInt(prompt("Enter an integer: "));`<br><br>`if (num % 29 === 0) {`<br>`    console.log(‘${num} is divisible by 29.‘);`<br>`} else {`<br>`    console.log(‘${num} is not divisible by 29.‘);`<br>`}`<br><br>3. C++:<br>`#include <iostream>`<br>`using namespace std;`<br><br>`int main() {`<br>`    int num;`<br>`    cout << "Enter an integer: ";`<br>`    cin >> num;`<br><br>`    if (num % 29 == 0) {`<br>`        cout << num << " is divisible by 29." << endl;`<br>`    } else {`<br>`        cout << num << " is not divisible by 29." << endl;`<br>`    }`<br><br>`    return 0;`<br>`}`<br><br>Replace the input mechanism with the one suitable for your program. The key point is using the modulo operator (%) to check if the remainder is zero when divided by 23. **[Correct]**

Table S5. Response given by ChatGPT on the question "*How to check if an integer can be divided by* 29".

<table>
<tr><th>Prime
N</th><th>Solution by ChatGPT on divisibility by N</th></tr>
<tr><td>31</td><td>

To check if an integer is divisible by 31, you can apply the modulo operation (%). If the remainder is 0, then the integer is divisible by 31. Here's how you can do this in different programming languages:

1. Python:

```
num = int(input("Enter an integer: "))
if num % 31 == 0:
    print(f"{num} is divisible by 31.")
else:
    print(f"{num} is not divisible by 31.")
```

2. JavaScript:

```
const num = parseInt(prompt("Enter an integer: "));

if (num % 31 === 0) {
    console.log(`${num} is divisible by 31.`);
} else {
    console.log(`${num} is not divisible by 31.`);
}
```

3. C++:

```
#include <iostream>
using namespace std;

int main() {
    int num;
    cout << "Enter an integer: ";
    cin >> num;

    if (num % 31 == 0) {
        cout << num << " is divisible by 31." << endl;
    } else {
        cout << num << " is not divisible by 31." << endl;
    }

    return 0;
}
```

Replace the input mechanism with the appropriate method for your program. The essential concept here is to use the modulo operator (%) to verify if the remainder is zero when the integer is divided by 31. **[Correct]**

</td></tr>
</table>

Table S6. Response given by ChatGPT on the question "*How to check if an integer can be divided by* 31".
